# Herbal Neurotherapeutics for Cognitive Disorders: Integrative Mechanisms Linking Neurotransmitter Systems, Neurodegeneration, and the Gut-Brain Axis

**DOI:** 10.3390/nu18111796

**Published:** 2026-06-02

**Authors:** Muntajin Rahman, Khadija Akter, Amama Rani, Moon Nyeo Park, Bonglee Kim

**Affiliations:** 1 Department of Pathology, College of Korean Medicine, Kyung Hee University, Seoul 02453, Republic of Korea; muntajinrahman899@gmail.com (M.R.); santaafrin02@gmail.com (K.A.); amama.rani@yahoo.com (A.R.); mnpark@khu.ac.kr (M.N.P.); 2 Department of Pharmacy, Northern University Bangladesh, Dakshinkhan, Dhaka 1230, Bangladesh; 3 Department of Microbiology, Quaid-i-Azam University, Islamabad 45320, Pakistan; 4 Korean Medicine-Based Drug Repositioning Cancer Research Center, College of Korean Medicine, Kyung Hee University, Seoul 02447, Republic of Korea

**Keywords:** Alzheimer’s diseases, acetylcholinesterase inhibitors, herbal compounds, neurotransmitter pathways, herbal medicine, neurological disorders

## Abstract

Cognitive disorders, including Alzheimer’s disease, Parkinson’s disease, schizophrenia, depression, and vascular dementia, are associated with dysregulation of neurotransmitter systems, including acetylcholine, dopamine, serotonin, glutamate, and γ-aminobutyric acid (GABA). These disorders are increasingly recognized as multifactorial conditions involving oxidative stress, neuroinflammation, mitochondrial dysfunction, synaptic impairment, blood–brain barrier disruption, metabolic imbalance, and gut–brain axis dysregulation. Current pharmacological therapies may provide symptomatic relief; however, their clinical benefits are often limited and associated with adverse effects. Herbal medicines have gained increasing attention as potential complementary approaches for cognitive support and neuroprotection. Preclinical evidence and emerging clinical studies suggest that herbal bioactive compounds may exert neuroprotective effects through antioxidants, anti-inflammatory, and neurotransmitter-modulating mechanisms. Medicinal herbs such as *Bacopa monnieri*, *Withania somnifera*, *Ginkgo biloba*, *Glycyrrhiza glabra*, *Moringa oleifera*, and ginseng have shown potential cognitive benefits in experimental models and selected human studies. Advanced delivery systems, including nanoparticles and phytosomes, may further improve the bioavailability and brain-targeting efficiency of herbal compounds. However, current clinical evidence remains heterogeneous and limited by insufficient standardization, small sample sizes, and short study durations. Further large-scale clinical studies and standardized safety assessments are essential before herbal neurotherapeutics can be widely applied in cognitive and neurological disorders.

## 1. Introduction

Concerns have arisen due to the increasing longevity of the elderly population, as many individuals now spend a substantial portion of their lives coping with age-related illnesses. Pathological cognitive decline is one of the most challenging conditions regarding personal impact and economic cost. Approximately one-third of individuals in the United States aged 85 years and above are affected by Alzheimer’s disease (AD), which remains a leading cause of pathological cognitive decline [1,2]. As global life expectancy increases, the incidence of age-associated neurological disorders is expected to rise substantially [3,4]. Cognitive impairment in the elderly is characterized by memory loss, learning challenges, and a decline in focus. This includes dementia and minor impairments that are not clinically evident [5]. These impairments may result from stroke, vascular dysfunction, neurodegeneration, or metabolic disturbances [6]. Individuals with cognitive impairment often experience reduced quality of life and increased risk of dementia and premature mortality [7,8].

The burden of dementia is increasing worldwide, currently affecting nearly 55 million people, with projections reaching 132 million cases by 2050 [9]. Reported prevalence estimates vary across Asian populations depending on diagnostic criteria and study design. For example, the prevalence of mild cognitive impairment (MCI) was 12.5% among older Singaporean Chinese adults, whereas cognitive impairment prevalence was 21.48% among older adults in Hunan Province, China [10,11]. A preliminary meta-analysis of community-dwelling older adults reported an overall pooled prevalence of 17.3%, although diagnostic criteria varied among included studies [12].

A preliminary meta-analysis of community-dwelling older adults reported an overall pooled prevalence of 17.3%, although diagnostic criteria varied among included studies [12].

Several biological mechanisms contribute to cognitive decline, including oxidative stress, mitochondrial dysfunction, intracellular calcium dysregulation, neuroinflammation, and neurotransmitter imbalance [13]. While amyloid-β accumulation has long been considered central to AD pathology, increasing evidence suggests that amyloid burden alone does not fully explain disease severity or progression [14,15,16]. Other mechanisms such as tau hyperphosphorylation, synaptic dysfunction, cholinergic deficits, mitochondrial dysfunction, and chronic neuroinflammation are also critically involved [17,18,19,20].

Enhancement of cognitive functioning is defined as amplification of fundamental mental capacities through improved internal and external information processing systems [21]. Cognitive enhancement can be achieved pharmacologically through cognitive enhancers [22], or non-pharmacologically through lifestyle-related factors such as regular physical activity, mental stimulation, adequate sleep, and balanced nutrition [23]. These non-pharmacological strategies may support general brain health and cognitive resilience; however, they are only briefly mentioned here as the present review primarily focuses on herbal neurotherapeutic approaches for cognitive disorders and their underlying mechanisms.

Emerging evidence also highlights the importance of the microbiota–gut–brain axis in cognitive health and neurodegenerative disorders. Gut microbiota can influence the synthesis and metabolism of key neurotransmitters such as serotonin, dopamine, and γ-aminobutyric acid (GABA), thereby modulating brain function and behavior [24,25,26]. Dysbiosis of the gut microbiome may promote systemic inflammation and increase intestinal permeability, allowing pro-inflammatory mediators such as lipopolysaccharides to enter circulation and subsequently impair blood–brain barrier integrity and central nervous system homeostasis [27,28]. Furthermore, microbial metabolites, particularly short-chain fatty acids (SCFAs) such as butyrate, propionate, and acetate, play a crucial role in regulating neuroinflammation, oxidative stress, epigenetic modulation, and synaptic plasticity [29,30,31]. Alterations in gut microbial composition have been increasingly associated with neurodegenerative disorders such as Alzheimer’s disease and Parkinson’s disease, as well as age-related cognitive decline [32,33].

Pharmacological strategies for cognitive impairment remain limited in efficacy and are frequently associated with adverse effects. Consequently, increasing attention has focused on herbal medicines and plant-derived bioactive compounds as multi-target therapeutic approaches capable of modulating oxidative stress, inflammation, neurotransmission, and neuronal survival.

The novelty of this review lies in its integrative framework linking neurotransmitter systems, neurodegenerative pathology, herbal neurotherapeutics, and the gut–brain axis within a single comprehensive model. Unlike previous reviews that address these topics separately, this article highlights how herbal interventions may simultaneously regulate central neurotransmission, peripheral metabolic pathways, neuroinflammation, and gut microbiota interactions to support cognitive health. This review therefore aims to summarize the mechanistic roles of herbal medicines in cognitive disorders, with particular emphasis on Alzheimer’s disease, Parkinson’s disease, depression-related cognitive dysfunction, and emerging gut–brain interactions.

## 2. Methods

The present study is a narrative review aimed at synthesizing current knowledge regarding the potential role of herbal neurotherapeutics in cognitive and neurological disorders, including Alzheimer’s disease, Parkinson’s disease, schizophrenia, depression, and vascular dementia. To maintain a structured approach while preserving the narrative scope of the review, a comprehensive literature search strategy was applied across different sections of the manuscript. A literature search was conducted using electronic databases including PubMed and Google Scholar. References were managed using EndNote X9 (Clarivate Analytics, Philadelphia, PA, USA). The search strategy utilized combinations of keywords and Medical Subject Headings (MeSH) terms, including “herbal medicine”, “cognitive impairment”, “Alzheimer’s disease”, “oxidative stress”, “neuroinflammation”, “gut–brain axis”, “nanoparticles”, and the names of medicinal herbs such as *Bacopa monnieri*, *Withania somnifera*, *Ginkgo biloba*, *Panax ginseng*, *Glycyrrhiza glabra*, and *Moringa oleifera*.

The literature search included peer-reviewed articles published in English. No strict publication restriction was applied in order to include both classical foundational studies and recent advances relevant to herbal neurotherapeutics and cognitive disorders. Both preclinical and clinical studies were considered; however, greater emphasis was placed on randomized controlled trials, systematic reviews, meta-analyses, and clinically relevant human studies where available. Studies discussing mechanisms of action, therapeutic effects, safety concerns, herb–drug interactions, hepatotoxicity, formulation strategies, and translational limitations were included to provide a balanced overview of the field. Where available, studies reporting neutral, conflicting, or safety-related findings were also considered. Furthermore, manual screening of reference lists from relevant review articles and clinical studies was performed to identify additional pertinent publications not captured during the initial database search.

## 3. Neurotransmitter Interaction and Cognitive Functions

Neurotransmitters are chemical messengers that regulate communication between neurons and play essential roles in cognition, memory, emotion, learning, and behavior [34]. Proper neurotransmitter balance is critical for maintaining normal brain function, synaptic plasticity, and neuronal survival. Dysregulation of neurotransmitter systems contributes to the development of several neuropsychiatric and neurodegenerative disorders, including Alzheimer’s disease (AD), Parkinson’s disease (PD), schizophrenia, depression, and vascular dementia [35,36]. Major neurotransmitters involved in cognitive regulation include acetylcholine, dopamine, glutamate, serotonin, and γ-aminobutyric acid (GABA) [37]. Alterations in these signaling pathways may impair neuronal communication and contribute to progressive cognitive dysfunction [38]. Therefore, understanding neurotransmitter-associated mechanisms remains important for the development of pharmacological and herbal therapeutic strategies targeting cognitive impairment and neurodegeneration.

### Neurotransmitters and Central Neurocognitive Abnormalities

Neurotransmitter imbalance is closely associated with cognitive impairment and central nervous system disorders [39,40]. Disruption of cholinergic, dopaminergic, glutamatergic, serotonergic, and GABAergic signaling pathways contributes to synaptic dysfunction, impaired neuronal plasticity, and progressive neurodegeneration [41,42,43]. Different neurotransmitter systems are implicated in distinct neurological and psychiatric disorders. Cholinergic dysfunction is strongly associated with memory impairment in AD, whereas dopaminergic degeneration contributes to motor and cognitive dysfunction in PD. Similarly, glutamatergic and GABAergic dysregulation are involved in schizophrenia, depression, and vascular cognitive impairment [43]. Because neurotransmitter alterations are central to disease pathogenesis, these pathways represent important therapeutic targets for both conventional pharmacological interventions and emerging herbal neurotherapeutics.

## 4. Disease-Related Cognitive Impairment

### 4.1. Cognitive Impairment in Degenerative Brain Disease

Alzheimer’s disease (AD) is the most common form of dementia and is strongly associated with ageing and genetic susceptibility. It is characterized by progressive degeneration of hippocampal and cortical neurons, leading to memory loss, executive dysfunction, and behavioral impairment [44,45]. The core issue involves neuronal dysfunction and the destruction of synapses in the hippocampus, cortex, and subcortical regions. This degeneration leads to significant atrophy in the afflicted areas, causing memory loss, mood swings, problems with executive function, trouble acquiring new knowledge, and the inability to conduct activities of daily living. This disorder is expected to affect 115.4 million people worldwide by 2050 [46] and represents one of the leading causes of death globally [47].

Mild cognitive impairment (MCI) represents an early clinical stage of AD, with approximately 10% of patients progressing annually to AD [48]. The pathological hallmark of AD involves amyloid-β (Aβ) deposition, tau hyperphosphorylation, synaptic dysfunction, and neuroinflammation. These processes converge to disrupt neuronal communication and network integrity, particularly in memory-associated brain regions. Importantly, AD pathogenesis is now understood as a multifactorial process in which protein aggregation interacts with metabolic stress, inflammatory signaling, and cellular dysfunction rather than a single causal pathway.

#### 4.1.1. Etiology of Alzheimer’s Disease

**Figure 1:**
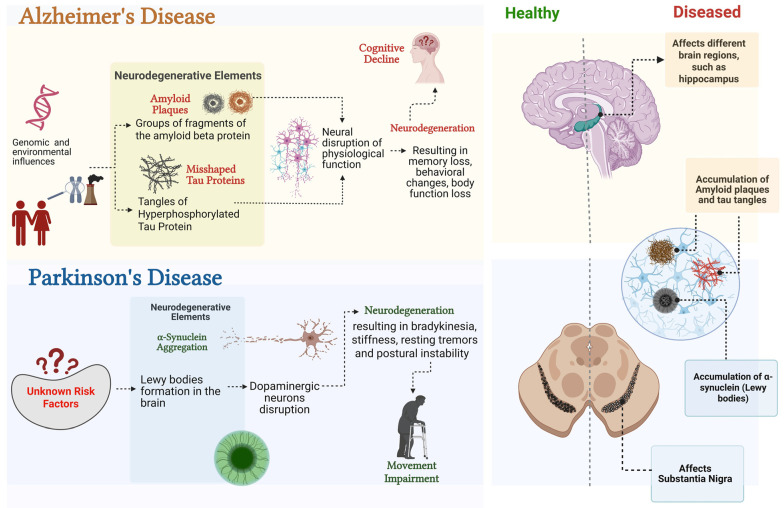
Degenerative features and affected brain regions in Alzheimer’s disease (AD) and Parkinson’s disease (PD). In AD, the accumulation of amyloid-β plaques and hyperphosphorylated tau tangles leads to neuronal disruption in the hippocampus and other cortical regions, resulting in cognitive decline, memory impairment, and behavioral abnormalities. Both genetic and environmental factors contribute to AD pathogenesis. In PD, aggregation of α-synuclein and Lewy bodies in the substantia nigra causes dopaminergic neuronal loss and characteristic motor symptoms. This schematic figure was recreated by the authors using BioRender based on published literature [54,57,58].

Aging remains the strongest risk factor for AD and is associated with progressive structural and functional brain changes, including synaptic loss, ventricular enlargement, and reduced brain volume. These changes are accompanied by metabolic and vascular alterations that contribute to increased vulnerability to neurodegeneration [49,50,51,52,53]. The classical amyloid cascade hypothesis describes Aβ accumulation as a central driver of disease progression, accompanied by tau pathology, synaptic failure, and neuronal loss. Aβ is generated through sequential cleavage of amyloid precursor protein (APP) by β- and γ-secretases, leading to oligomer formation that exerts neurotoxic effects on synapses [54,55,56]. As shown in Figure 1, these pathological features collectively contribute to progressive neuronal dysfunction and cognitive decline in AD.

Beyond these classical mechanisms, growing evidence supports a broader pathogenic network in AD. Oxidative stress, mitochondrial dysfunction, and neuroinflammatory activation are closely interconnected and collectively contribute to neuronal injury and Aβ accumulation [59,60]. Excessive ROS production, impaired mitochondrial energy metabolism, and glial activation amplify synaptic dysfunction and blood–brain barrier (BBB) disruption, thereby accelerating disease progression [61,62], Moreover, APP metabolism and secretase activity are influenced by oxidative membrane damage, further linking metabolic stress to amyloid pathology. Although the temporal sequence of these events remains debated, it is widely accepted that AD results from a dynamic interaction between protein aggregation and cellular stress pathways rather than a linear cascade [50,63].

In addition to amyloid-β accumulation and tau hyperphosphorylation, Alzheimer’s disease is now recognized as a multifactorial disorder involving several emerging pathogenic mechanisms. Ferroptosis, an iron-dependent form of regulated cell death characterized by lipid peroxidation, has been increasingly implicated in neuronal loss in Alzheimer’s disease [64,65]. Impairment of autophagic and lysosomal pathways further contributes to the accumulation of misfolded proteins and defective cellular clearance mechanisms [66,67]. Neurovascular dysfunction, including blood–brain barrier disruption and reduced cerebral blood flow, also plays a critical role in disease progression [68,69]. In addition, insulin resistance within the central nervous system, often described as “type 3 diabetes,” has been associated with impaired glucose metabolism and cognitive decline [70,71]. Furthermore, epigenetic regulation, including DNA methylation, histone modifications, and non-coding RNA-mediated mechanisms, has emerged as an important modulator of gene expression in Alzheimer’s pathology [72,73]. Collectively, these mechanisms highlight the complex and heterogeneous nature of Alzheimer’s disease beyond the classical amyloid cascade hypothesis.

#### 4.1.2. Acetylcholine and Alzheimer’s Disease

The cholinergic hypothesis of AD emerged in the 1970s following evidence of reduced choline acetyltransferase (ChAT) activity in the neocortex, indicating impaired acetylcholine (ACh) synthesis [74]. ACh deficiency is one of the most consistent neurochemical alterations in AD and is closely linked to cognitive decline.

Degeneration of basal forebrain cholinergic neurons, particularly in the nucleus basalis of Meynert, leads to reduced cholinergic projections to the cortex and hippocampus. This disruption contributes to impaired synaptic signaling and progressive cognitive dysfunction [75,76]. Acetylcholinesterase (AChE) regulates synaptic ACh levels by enzymatic degradation. Increased AChE activity reduces cholinergic transmission and worsens cognitive deficits. Therefore, AChE dysfunction is directly associated with disease progression [77]. Although amyloid-β and tau-targeted therapies have shown limited clinical success, cholinergic dysfunction remains a validated therapeutic target in AD. Current symptomatic treatments aim to enhance cholinergic signaling and improve cognitive performance in mild to moderate stages of the disease [78].

#### 4.1.3. Cholinergic System-Targeting Drug in Alzheimer’s Disease

The cholinergic system plays a central role in learning, memory, and cortical network activity. Acetylcholine is synthesized by choline acetyltransferase using choline, acetyl-CoA, and ATP as substrates [79]. It acts as a key modulator of synaptic transmission and neuronal communication. In the central nervous system, cholinergic signaling regulates synaptic plasticity and supports cognitive processing. Reduced cholinergic tone is strongly associated with memory impairment and neurodegeneration in AD [80].

Acetylcholine is rapidly degraded by acetylcholinesterase (AChE). Therefore, inhibition of AChE increases synaptic ACh availability and enhances cholinergic transmission. This mechanism forms the basis of current symptomatic therapies for AD [81]. Cholinesterase inhibitors, including donepezil, rivastigmine, galantamine, and tacrine, are widely used in clinical practice. These agents may provide modest improvement in cognitive and behavioral symptoms; however, they do not prevent disease progression [82].

Overall, cholinergic enhancement remains one of the most established pharmacological strategies for symptomatic management of AD. Current therapeutic approaches primarily focus on symptomatic relief and slowing disease progression. Clinically approved pharmacological treatments include cholinesterase inhibitors such as donepezil, rivastigmine, and galantamine, which improve cholinergic neurotransmission and may provide modest benefits in cognition and daily functioning [83,84]. Memantine, an *N*-methyl-D-aspartate (NMDA) receptor antagonist, is commonly prescribed for moderate-to-severe AD and may help reduce excitotoxic neuronal injury [85].

More recently, anti-amyloid monoclonal antibodies targeting amyloid-β pathology have emerged as potential disease-modifying therapies in selected patient populations, although concerns regarding cost, accessibility, imaging abnormalities, and long-term efficacy remain under investigation [86,87]. In addition to pharmacological interventions, non-pharmacological approaches including cognitive rehabilitation, physical activity, sleep optimization, vascular risk reduction, and caregiver-centered support also play important roles in comprehensive AD management [88]. Although herbal neurotherapeutics have shown potential neuroprotective and cognitive-supportive effects in experimental and selected clinical studies, the current evidence remains heterogeneous and insufficient to replace established therapies. Therefore, further large-scale, standardized clinical investigations are required to validate their long-term efficacy and safety [89].

#### 4.1.4. Acetylcholinesterase Inhibitors

Cholinesterase inhibitors represent the primary symptomatic pharmacological treatment for AD. These agents do not alter disease progression, but they improve cognition and behavioral symptoms [90]. This effect is achieved through enhancement of cholinergic neurotransmission via inhibition of AChE and, in some cases, butyrylcholinesterase (BuChE) [91,92]. Cholinergic dysfunction in AD is closely associated with memory impairment and attentional deficits, making this pathway a key symptomatic therapeutic target [93]. Clinically approved AChE inhibitors include tacrine, donepezil, rivastigmine, and galantamine [90,94,95,96]. These agents provide modest symptomatic benefit in mild to moderate AD, mainly improving memory, attention, and daily functioning, but do not prevent neurodegeneration [97,98].

Donepezil is a widely used first-line therapy due to its favorable tolerability and once-daily dosing [99]. Rivastigmine inhibits both AChE and BuChE and is also used in PD and dementia [100]. Galantamine additionally acts as a nicotinic receptor modulator, potentially influencing broader neurotransmitter systems beyond cholinergic signaling [101]. Tacrine, the first approved AChE inhibitor, is no longer used clinically due to hepatotoxicity [102]. Despite pharmacological differences, all AChE inhibitors share a common mechanism of enhancing cholinergic transmission, resulting in temporary cognitive stabilization and symptomatic relief [77]. However, their limited disease-modifying effect highlights the need for multi-target strategies, including herbal neurotherapeutics with broader neuroprotective and anti-inflammatory actions.

### 4.2. Cognitive Impairment in Parkinson’s Disease

Cognitive impairment is one of the most important non-motor symptoms of Parkinson’s disease (PD) and a major determinant of disease progression. It occurs significantly more frequently in PD patients than in the general population, with an estimated increase of up to six-fold [103]. Even in early stages, cognitive decline negatively affects daily functioning and quality of life. It also contributes to substantial economic and caregiving burden. Parkinson’s disease is a progressive neurodegenerative disorder characterized by the accumulation of misfolded α-synuclein, which forms Lewy bodies and Lewy neurites within neurons (Figure 1) [57]. These pathological aggregates are closely associated with widespread neurotransmitter dysfunction and neuronal loss across multiple brain regions.

One of the most significant non-motor symptoms of PD is dementia, which results from widespread neurotransmitter disruptions and the degeneration of cortical neurons across various brain regions. It frequently includes deficits in speech, memory, and visuospatial abilities in addition to executive dysfunction. The prevalence of this impairment is rising among PD patients, especially those with recent diagnoses [104]. The intricate cause of cognitive impairment in PD likely involves the progressive deterioration of multiple brain networks [58]. As illustrated in Figure 1, these changes arise from α-synuclein aggregation, mitochondrial dysfunction, oxidative stress, and excitotoxicity.

Dopaminergic degeneration in the substantia nigra pars compacta is a hallmark of PD and leads to motor symptoms, followed by cognitive decline and dementia. The loss of dopaminergic neurons is strongly associated with α-synuclein aggregation and progressive neurodegeneration. In addition, reduced dopamine levels contribute to impaired cortical and subcortical network function [57,105].

#### 4.2.1. Etiology of Parkinson’s Disease

The etiology of Parkinson’s disease is multifactorial and involves interacting molecular and cellular mechanisms. The most widely accepted pathological contributors include α-synuclein aggregation, mitochondrial dysfunction, oxidative stress, and excitotoxicity [106]. The accumulation of α-synuclein follows a progressive pattern described by Braak staging. Early pathology begins in the dorsal motor nucleus of the vagus nerve and olfactory structures, and gradually spreads to the brainstem and neocortex [107].

The precise mechanism of α-synuclein misfolding remains unclear; however, an imbalance between protein production and clearance is considered central. Genetic alterations such as SNCA duplication or triplication further accelerate aggregation [108].

Moreover, the etiology of Parkinson’s disease is directly linked to oxidative stress and mitochondrial dysfunction (Figure 1).

Mitochondrial dysfunction plays a key role in disease progression by reducing ATP production and increasing reactive oxygen species (ROS). This contributes to neuronal injury and cell death. Mutations in genes such as SNCA and LRRK2, as well as mitochondrial DNA abnormalities, impair mitochondrial dynamics including fission and fusion processes [109]. Oxidative stress results from an imbalance between ROS production and antioxidant defense systems, leading to cellular dysfunction. Additionally, α-synuclein aggregates further disrupt mitochondrial integrity by promoting cytochrome c release and membrane depolarization [110,111].

Excitotoxicity also contributes to neurodegeneration in PD. Excess glutamate activity overstimulates NMDA receptors, leading to calcium overload and neuronal damage. Abnormal glutamate signaling in the substantia nigra is associated with progressive dopaminergic loss [112]. Several phytochemicals have been investigated for their neuroprotective potential in PD. Curcumin, resveratrol, and quercetin exhibit antioxidant and anti-inflammatory properties that may reduce oxidative stress and mitochondrial dysfunction [113]. Mucuna pruriens contains natural L-DOPA and may support dopaminergic signaling [114]. Ginkgo biloba may enhance cerebral blood flow and provide neuroprotection through antioxidant mechanisms [115].

#### 4.2.2. Dopamine and Parkinson’s Disease

Cognitive dysfunction in Parkinson’s disease is closely linked to dopaminergic deficits. Dopamine depletion in the substantia nigra pars compacta disrupts cortico-striatal circuits involved in memory, attention, and executive function [116]. In addition to dopamine loss, glutamatergic overactivity contributes to excitotoxic neuronal injury. Overactivation of NMDA receptors increase intracellular calcium influx and promotes oxidative stress, leading to neuronal dysfunction. α-Synuclein accumulation further exacerbates glutamate dysregulation and mitochondrial impairment [117]. Neurons in the substantia nigra are particularly vulnerable due to their high metabolic demand and susceptibility to oxidative stress. Plant-derived compounds may help modulate these pathological processes. Curcumin and resveratrol reduce oxidative stress and inhibit neuroinflammation [89]. Mucuna pruriens supports dopamine availability through its L-DOPA content, while green tea polyphenols may protect neurons by reducing excitotoxicity and oxidative damage [118].

#### 4.2.3. Current Therapeutic Strategies in Parkinson’s Disease

The current clinical management of Parkinson’s disease (PD) primarily focuses on improving motor and non-motor symptoms through dopaminergic therapies. Levodopa remains the most effective and widely used treatment for symptomatic control, particularly for bradykinesia and rigidity [119]. Plant-derived compounds may help modulate these pathological processes. Curcumin and resveratrol reduce oxidative stress and inhibit neuroinflammation [89]. Mucuna pruriens supports dopamine availability through its L-DOPA content, while green tea polyphenols may protect neurons by reducing excitotoxicity and oxidative damage [118]. Additional pharmacological approaches include monoamine oxidase-B (MAO-B) inhibitors and catechol-O-methyltransferase (COMT) inhibitors, which help prolong dopamine activity and reduce motor fluctuations [120]. Dopamine agonists and anticholinergic agents may also be used in selected patients depending on disease stage and symptom severity [121].

Despite their clinical benefits, these therapies may be associated with complications such as dyskinesia, hallucinations, sleep disturbances, and reduced efficacy over time [122]. Consequently, comprehensive PD management also incorporates non-pharmacological interventions including regular exercise, physical rehabilitation, speech and occupational therapy, sleep optimization, cognitive support, and caregiver-centered strategies to improve mobility, functional independence, and quality of life [123].

In addition to conventional therapies, increasing attention has been directed toward herbal compounds and phytochemicals with potential neuroprotective properties. Experimental studies suggest that compounds such as curcumin, resveratrol, ginsenosides, and green tea polyphenols may exert antioxidant, anti-inflammatory, and mitochondrial-protective effects relevant to PD pathology [124]. Mucuna pruriens, a natural source of L-DOPA, has also been investigated for its potential supportive role in dopaminergic regulation [125]. However, despite these promising experimental findings, the current clinical evidence remains limited and heterogeneous. Therefore, herbal neurotherapeutics should currently be considered complementary rather than replacement therapies, and further large-scale, standardized clinical studies are required to establish their long-term efficacy and safety [126].

#### 4.2.4. Dopaminergic Strategies for the Treatment of Parkinson’s Disease

At present, PD remains incurable, as no available treatment can halt or even slow its progression, despite significant efforts from various research organizations aimed at [127,128]. Nonetheless, a variety of dopaminergic and nondopaminergic therapies for treating both motor and non-motor symptoms of PD can significantly enhance the quality of life for patients over many years [129,130,131]. Dopaminergic agents, in particular, constitute the cornerstone of motor symptom management. Table 1 presents a summary of FDA-approved pharmacological treatments for both PD and Alzheimer’s disease (AD), including their mechanisms of action and therapeutic targets.

### 4.3. Serotonin and Cognitive Impairments

Serotonin (5-hydroxytryptamine, 5-HT) is a key neurotransmitter involved in neurodevelopment and the regulation of cognitive and emotional processes [156]. The serotonergic system plays an important role in learning and memory, mainly through its interactions with the cholinergic, glutamatergic, dopaminergic, and GABAergic systems [157]. These interactions support higher brain functions, particularly within the septohippocampal complex and the nucleus basalis magnocellularis–frontal cortex network. Serotonin is synthesized from the amino acid tryptophan and is widely distributed in serotonergic neurons, enterochromaffin cells, and blood platelets [158]. It regulates diverse central nervous system functions through serotonin receptors (5-HT receptors), which are classified into seven subfamilies (5-HT1–5-HT7) [159]. Most of these receptors belong to the G protein-coupled receptor family, except 5-HT3 receptors, which function as ligand-gated ion channels [160]. Alterations in serotonergic signaling have been implicated in several neuropsychiatric and neurodegenerative disorders, including depression, anxiety, schizophrenia, Alzheimer’s disease, and age-related cognitive decline [161]. Serotonergic dysfunction is increasingly recognized as a contributor to cognitive impairment. Therefore, targeting serotonin pathways has become an important therapeutic strategy for cognitive and mood-related disorders [162].

Selective serotonin reuptake inhibitors (SSRIs) remain the first-line pharmacological treatment for depression [163].

Common SSRIs include citalopram, escitalopram, fluoxetine, fluvoxamine, paroxetine, and sertraline. Although they share a common mechanism of serotonin reuptake inhibition, they differ in pharmacokinetic properties and side effect profiles, which guide clinical selection. Evidence suggests that SSRIs may also exert beneficial effects on certain cognitive domains, including psychomotor performance, executive function, and memory in patients with depression [164,165,166].

Alongside conventional pharmacotherapy, increasing attention has been given to plant-derived compounds that may modulate serotonergic signaling. Phytochemicals such as hyperforin (from *Hypericum perforatum*), crocin (from *Crocus sativus*), and curcumin have demonstrated potential antidepressant-like and neurocognitive effects through modulation of serotonin pathways [167]. These findings suggest that herbal compounds may serve as adjunct strategies for improving serotonergic balance and cognitive outcomes [168].

### 4.4. Cognitive Impairments in Schizophrenia

Schizophrenia is a prevalent and debilitating mental disorder characterized by psychotic symptoms, negative symptoms, and cognitive impairments, including deficits in executive function and working memory [169].

It is commonly classified into three symptom domains: cognitive deficits, affective flattening, reduced emotional expression (alogia), and positive symptoms such as delusions and perceptual disturbances [170,171]. Although cognitive dysfunction has long been recognized in schizophrenia, recent studies increasingly identify it as a major contributor to functional impairment [172].

#### 4.4.1. Etiology of Schizophrenia

Neuroimaging studies show that cognitive deficits in schizophrenia are associated with reduced cortical thickness [173,174]. Sex differences have been reported, with stronger effects in women [175]. Additional structural and functional abnormalities include enlarged ventricles, reduced cerebellar volume, impaired basal ganglia function, and loss of dendritic spines in the dorsolateral prefrontal cortex [176,177]. These changes may reflect disruption of cortico-cerebellar-thalamic-cortical circuits and reduced prefrontal metabolic activity [178]. Stress-related hormonal changes also contribute. Elevated cortisol can cross the blood–brain barrier and affect the amygdala, hippocampus, and prefrontal cortex [179,180]. Increased cortisol is linked to reduced BDNF expression, hippocampal atrophy, and poorer cognitive performance [181,182].

Prolactin dysregulation is also associated with cognitive decline, although interpretation is complicated by antipsychotic effects [183,184]. Neurotransmitter dysregulation, particularly involving glutamate, GABA, and dopamine pathways, contributes significantly to cognitive impairment in schizophrenia [185,186]. Glutamatergic dysfunction in the dorsolateral prefrontal cortex has been associated with impaired working memory and abnormal cortical signaling [187,188]. In addition, NMDA receptor hypofunction, altered kynurenine metabolism, and neuroinflammatory processes may further contribute to excitatory–inhibitory imbalance and cognitive dysfunction [189,190,191,192]. Genetic susceptibility factors associated with neurotransmission and synaptic regulation may also contribute to schizophrenia-related cognitive dysfunction [193].

#### 4.4.2. Glutamate Hypothesis of Schizophrenia and Its Implication for the Treatment

Glutamate is the most abundant excitatory neurotransmitter in the brain. Over the last 25 years, pharmacological, neuroimaging, and genetic studies have highlighted glutamatergic dysregulation in the pathophysiology of schizophrenia, with recent clinical trials reporting promising therapeutic outcomes. Glutamatergic dysregulation is increasingly recognized as an important mechanism underlying cognitive dysfunction in schizophrenia [194,195]. In particular, NMDA receptor hypofunction may disrupt excitatory–inhibitory balance and impair cortical signaling involved in cognition and working memory [196]. However, current evidence suggests that schizophrenia-related glutamatergic abnormalities involve complex dysregulation rather than simple glutamate reduction alone [197,198]. Overall, current evidence suggests that schizophrenia-related glutamatergic dysfunction reflects an imbalance in excitatory neurotransmission involving both NMDA receptor hypofunction and altered non-NMDA receptor activity. From a therapeutic perspective, several phytochemicals and herbal compounds with glutamatergic modulatory and neuroprotective properties, including flavonoids and alkaloids, have been explored as potential adjunctive approaches for supporting excitatory–inhibitory balance in schizophrenia [199,200].

### 4.5. Cognitive Impairment in Depression

#### 4.5.1. Gamma-Aminobutyric Acid (GABA) and Cognitive Function in Depression

In addition to schizophrenia, major depressive disorder is also strongly associated with alterations in inhibitory neurotransmission, particularly involving the GABAergic system. In addition to schizophrenia, major depressive disorder is also associated with alterations in inhibitory neurotransmission, particularly involving the GABAergic system. GABA is the principal inhibitory neurotransmitter in the brain and plays an important role in regulating neuronal excitability, hippocampal activity, learning, and cognitive processing [201,202]. Reduced GABAergic signaling and impaired inhibitory balance have been associated with cognitive dysfunction, stress-related pathology, and depressive symptoms [203,204]. These findings suggest that GABAergic dysfunction may contribute to cognitive impairment observed in depression and related affective disorders. It is interesting to note that GABAergic abnormalities associated with MDD generally appear to impact neurons that express SST. As a result, one of the most promising endophenotypes for therapeutic targets in depression is thought to have low GABA levels [205]. While schizophrenia is primarily associated with glutamatergic and dopaminergic dysfunction, similar inhibitory neurotransmitter alterations are also observed in affective disorders such as depression.

#### 4.5.2. GABAergic Hypothesis in Depression and Its Implications for Treatment

Increasing evidence suggests that GABAergic dysfunction plays an important role in the pathophysiology of major depressive disorder [206,207]. GABA, the principal inhibitory neurotransmitter in the central nervous system, regulates neuronal excitability, stress responses, emotional processing, and cognitive function. Alterations in GABAergic neurotransmission have been associated with impaired brain plasticity, memory deficits, and mood disturbances observed in depression [204,205,208]. These functions are largely dependent on GABAergic neurons [209]. Reduced GABA levels have been reported in the plasma, cerebrospinal fluid, and cortical tissues of patients with depression, particularly in treatment-resistant cases [210]. In addition, dysregulation of glutamatergic and GABAergic signaling contributes to excitatory–inhibitory imbalance, which is increasingly recognized as a key mechanism underlying depressive disorders [211,212,213]. GABAergic dysfunction may also promote hyperactivity of the hypothalamic–pituitary–adrenal (HPA) axis through impaired inhibition of corticotropin-releasing factor signaling, thereby contributing to elevated cortisol levels and stress-related pathology [214]. Current antidepressant strategies, including selective serotonin reuptake inhibitors and ketamine, may partially exert their therapeutic effects through modulation of glutamatergic and GABAergic signaling pathways [205,215].

### 4.6. Vascular Dementia and Glutamatergic Dysfunction

Beyond psychiatric disorders such as schizophrenia and depression, glutamatergic dysregulation also contributes to neurodegenerative conditions, including vascular dementia. Cerebral ischemia and chronic hypoperfusion can promote excessive glutamate release, leading to excitotoxic neuronal injury, synaptic dysfunction, and cognitive decline [216,217]. Disturbances in intracellular calcium homeostasis may further amplify neuronal damage under ischemic conditions [218]. Pharmacological agents targeting calcium signaling, including nimodipine, have demonstrated potential neuroprotective effects in vascular dementia and cerebrovascular injury [219]. For instance, nimodipine, an L-type VGCC channel antagonist with FDA approval, is used to treat and prevent brain damage following aneurysmal subarachnoid hemorrhage [219]. Previous research has indicated that nimodipine is a useful treatment for VD. In addition to inhibiting Ca^2+^ inward flow, insulin, 4,1-benzothiazepines, and CX3CR1107 also have neuroprotective properties. When taken as drugs, they might help relieve VD [220,221,222].

Given the multifactorial mechanisms involved in vascular cognitive impairment, increasing attention has been directed toward herbal medicines with potential neuroprotective properties. Several medicinal plants and phytochemicals possess antioxidant, anti-inflammatory, cholinesterase-inhibitory, and neuroprotective activities that may help support neuronal function and cognitive health [223,224]. In addition, certain herbal formulations may influence multiple pathological pathways involved in neurodegeneration, suggesting possible complementary roles in vascular cognitive disorders. However, further large-scale clinical studies and standardized evaluations are required to establish their long-term efficacy and safety.

### 4.7. Emerging Integrative Mechanisms Linking Cognitive Impairment, Neurodegeneration, and the Gut–Brain Axis

In addition to classical mechanisms such as neurotransmitter imbalance, oxidative stress, and neuroinflammation, several emerging pathways contribute to cognitive impairment across neuropsychiatric and neurodegenerative disorders. Gut microbiota dysbiosis has been increasingly associated with cognitive dysfunction in Alzheimer’s disease, Parkinson’s disease, schizophrenia, and major depressive disorder. Altered microbial composition may influence brain function through immune activation, impaired intestinal barrier integrity, systemic inflammation, and changes in neurotransmitter and metabolite production, thereby contributing to gut–brain axis dysfunction and synaptic impairment.

Microbial metabolites, including short-chain fatty acids, bile acid derivatives, and tryptophan metabolites, regulate microglial activation, blood–brain barrier integrity, synaptic plasticity, and serotonergic signaling. In parallel, neurovascular dysfunction, impaired metabolic signaling, and mitochondrial abnormalities may further contribute to reduced cerebral blood flow, neuronal energy deficits, tau hyperphosphorylation, and cognitive decline across multiple disorders.

At the cellular level, impaired autophagy and lysosomal clearance contribute to the accumulation of damaged proteins, dysfunctional mitochondria, and oxidative stress, which are commonly observed in neurodegenerative disorders. Emerging evidence also implicates ferroptosis and epigenetic dysregulation, including altered DNA methylation, histone modification, and microRNA signaling, in the progression of cognitive impairment and neuroinflammatory responses.

Importantly, these pathways are interconnected rather than independent. Gut microbiota dysbiosis may influence metabolic signalling, immune activation, and epigenetic regulation. Vascular dysfunction and mitochondrial impairment further exacerbate neuroinflammation and neurotransmitter imbalance. Together, these interactions support a systems-level model of cognitive decline across disorders. Several phytochemicals and herbal bioactives including curcumin, resveratrol, ginsenosides, bacosides, and epigallocatechin gallate, have demonstrated multi-target biological activities involving oxidative stress, inflammation, metabolism, and gut microbiota regulation. Therefore, these compounds may represent potential supportive strategies for maintaining gut–brain–immune–metabolic homeostasis in cognitive disorders.

Targeting these integrated mechanisms may provide a broad strategy for reducing cognitive decline across psychiatric and neurodegenerative diseases. A summary of these mechanisms and their associated herbal targets is provided in Table 2.

## 5. Herbal Medicine in Promoting Cognitive Health and Memory Resilience: Mechanisms

**Figure 2:**
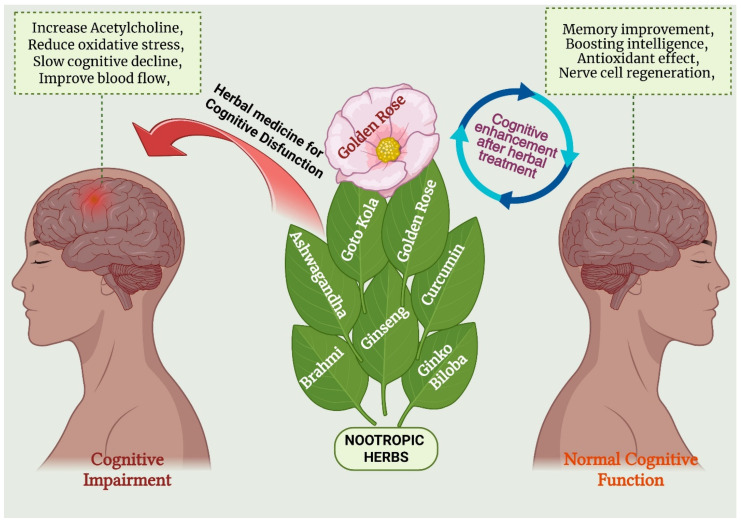
Overview of the therapeutic effects of nootropic herbs on cognitive health. These herbs enhance memory, cognition, and overall brain function by increasing neurotransmitter levels, particularly acetylcholine, through inhibition of acetylcholinesterase (AChE) [253]. They also improve cerebral blood circulation, support neuronal signaling, and protect against oxidative stress. Many natural nootropics are derived from common dietary plants and are widely used globally, with nearly 80% of the world’s population relying on herbal medicines for general healthcare [254,255,256]. The red arrow indicates the application of herbal interventions for cognitive impairment, the blue circular arrows represent cognitive enhancement and recovery following herbal treatment, the green leaves symbolize nootropic herbal medicines, and the green dashed lines indicate the association between the listed biological effects and the corresponding cognitive states.

Herbal medicine has been used worldwide for centuries to improve impaired memory and related disorders [244,245]. Evidence suggests that specific medicinal plants possess cognition-enhancing properties mediated by bioactive phytochemicals with neuroprotective potential [245,246]. The growing international demand for plant-based therapeutics is partly attributed to the perception that natural phytoconstituents are associated with lower toxicity and fewer adverse effects compared with many synthetic compounds [247]. Numerous medicinal plants used in traditional Chinese, Japanese, Korean, African, American, and European medicine have been reported to alleviate memory impairment and cognitive dysfunction. The overall therapeutic effects of nootropic herbs on cognitive health are illustrated in Figure 2.

Plants with neuroactive properties are designated as botanical neuroenhancers, whereas their active phytochemicals are identified as neuroactive phytochemicals [248]. Administration of natural nootropics may support neuronal resilience against toxic insults and age-related cognitive decline. Natural nootropics may support cognitive function through modulation of neurotransmitter systems, enhancement of cerebral circulation, and maintenance of neuronal integrity. Some phytochemicals exhibit vasodilatory properties that promote cerebral blood flow and oxygen delivery to neuronal tissues [249]. These effects may support neuronal activity, and cognitive processing [248]. Commercially available natural nootropics exert their effects through multiple neuropharmacological mechanisms. These compounds can modulate neurotransmitter concentrations and signaling pathways, including increased dopamine release, enhancement of cholinergic neurotransmission, modulation of AMPA receptor activity, stimulation of phosphatidylinositol turnover, and activation of phospholipase A2 pathways [250]. Certain naturally occurring nootropics also function as positive allosteric modulators of glutamatergic or cholinergic receptors [251]. Collectively, these mechanisms may support synaptic transmission, neuronal communication, and processes involved in learning and memory formation [252].

Medicinal herbs may also inhibit acetylcholinesterase (AChE) activity, thereby increasing acetylcholine availability in the brain and supporting cholinergic neurotransmission [250]. In addition, several phytochemicals exhibit antioxidant and anti-inflammatory properties that may reduce oxidative stress and neuroinflammatory responses associated with neurodegenerative disorders.

### 5.1. Herbal Medicines in Cognitive Enhancement and Disorder Management

Since ancient times, herbal remedies have been used to support cognitive function and promote brain health. Emerging evidence suggests that certain herbal compounds may have potential supportive roles in neurodegenerative disorders such as Alzheimer’s disease and Parkinson’s disease. Some herbal extracts, including *Ginkgo biloba*, *Panax ginseng*, and *Bacopa monnieri*, have been investigated for their possible neuroprotective properties and their ability to support memory, attention, synaptic plasticity, and cerebral circulation [257,258]. In addition, phytochemicals such as curcumin, ashwagandha, and *Centella asiatica* have shown antioxidant and anti-inflammatory activities in experimental and limited clinical studies, suggesting possible supportive effects against neurodegenerative processes [259]. However, despite encouraging preclinical findings, the clinical evidence remains heterogeneous, and further well-designed large-scale trials are necessary to confirm their long-term efficacy and safety in cognitive disorders. Recent phytochemical profiling studies, including HPLC-ESI-QTOF-MS/MS analyses of saffron corms, continue to identify diverse bioactive constituents with potential pharmacological relevance [260].

### 5.2. Mechanism of Action of Herbal Medicine in Disorder Management

**Figure 3:**
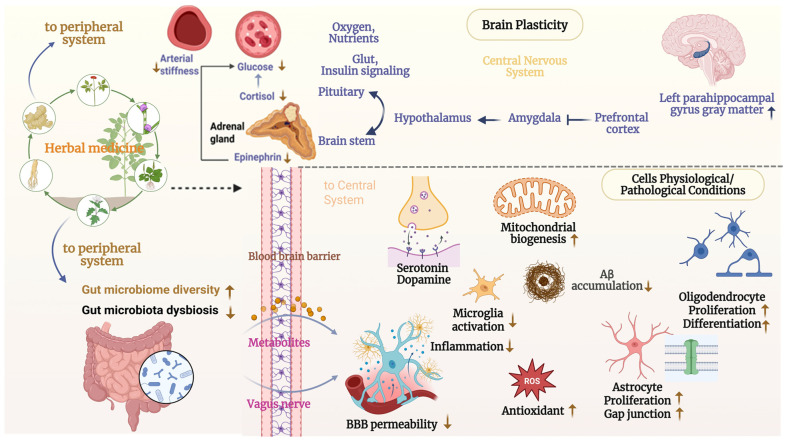
Proposed mechanisms through which herbal medicines exert neuroprotective and cognitive-enhancing effects via peripheral, central, and gut–brain pathways. Herbal extracts modulate peripheral physiology by reducing arterial stiffness, lowering glucose and stress-related hormones (cortisol, epinephrine), and improving systemic metabolic balance. These changes enhance vascular function and oxygen-nutrient delivery to the brain. Herbal compounds also cross the blood–brain barrier (BBB) or act through metabolites to influence central neurotransmission, increasing serotonin and dopamine levels, enhancing mitochondrial biogenesis, reducing Aβ accumulation, modulating oligodendrocyte activity, and decreasing microglial activation and neuroinflammation. Gut–brain axis modulation is mediated through increased microbial diversity, reduced dysbiosis, metabolite signaling, and vagus-nerve pathways, collectively reducing inflammation, oxidative stress, and BBB permeability. These integrated molecular and cellular interactions may support cognitive function and neuroprotection while potentially influencing fatigue, mood, and neuroinflammatory responses [267,268,269,270].

Herbal medicine exerts therapeutic effects by targeting key biological pathways, including neurotransmitter regulation, enhancement of neurogenesis, reduction in neuroinflammation, and antioxidant protection. These processes collectively contribute to neuronal survival, synaptic transmission, and brain plasticity. While herbal medicines act through multiple mechanisms, their neuroprotective effects are increasingly associated with the modulation of the peripheral system, the central nervous system, and the gut–brain axis [261,262]. Herbal medicines may influence neuroplasticity and higher-order brain functions by modulating neuronal activity, glial cell function, peripheral hormone secretion, and gut microbial composition (Figure 3) [263]. While many herbs are traditionally used to manage pathological conditions, they are also frequently consumed by healthy individuals, especially in Asian countries, to enhance cognitive performance, improve physical vitality, and delay age-related cognitive decline. Understanding how these herbal interventions affect brain health during the normal aging process is therefore important. Through actions on interconnected biological systems, herbal medicines enhance neurotransmitter signaling, regulate stress-related hormones, improve metabolic balance, and promote beneficial gut microbiota changes, collectively supporting cognitive resilience and healthy brain aging.

Herbal medicines also operate through the gut–brain–microbiota axis, a pathway increasingly recognized as essential in neurodegenerative disorders such as AD. After oral consumption, herbal compounds are transformed and absorbed in the gut, where they interact with intestinal microbiota. This interaction alters microbial composition, enhances beneficial bacteria, and reduces dysbiosis, leading to increased production of short-chain fatty acids (SCFAs) and decreased inflammatory cytokines [264]. These gut-mediated changes strengthen intestinal barrier function and improve central nervous system homeostasis. Given the strong association between gut microbiota dysregulation and AD, these secondary effects may partially contribute to the proposed neuroprotective properties of certain herbal medicines. Several herbs and plant formulations have shown the ability to regulate gut microbial populations, thereby influencing cognitive outcomes. Numerous plant formulations have demonstrated the capacity to control gut microbial populations, which in turn affects cognitive function. A classified summary of herbal and natural substances that have been shown to improve memory and cognition is given in Table 3, together with information on their bioactive components, medicinal applications, and modes of action. Figure 3 summarizes the integrated peripheral, central, and gut-mediated mechanisms through which herbal medicines support cognitive function and neuroprotection.

Through modulation of gut microbiota composition and inflammatory signaling pathways, certain herbal compounds may help influence neuroinflammatory responses, β-amyloid pathology, and neuronal homeostasis via the microbiota–gut–brain axis [265]. Regulation of inflammatory signaling pathways such as NF-κB and MAPK remains central to neuroprotection. Supporting evidence from related cellular models has shown that nonthermal plasma jet suppresses inflammatory responses through modulation of the NF-κB and MAPK pathways in human coronavirus 229E-infected lung cells [266]. However, these mechanisms remain incompletely understood and require further clinical validation.

**Table 3:** Nutrients, phytochemicals, medicinal plants, and clinically validated herbal interventions associated with cognitive enhancement and neuroprotection.

Category/Intervation	Source/Bioactive Chemical constituents	Family/Type	Therapeutic Properties	Mechanism of Action	Ref.
**A. Nutrients and Endogenous Compounds**					
Amino acids and proteins	L-carnitine, L-cysteine, L-glutamine, L-phenylalanine, L-tryptophan, L-tyrosine	Amino acid	Neurotransmission, brain metabolism, fatigue reduction, neuroprotection	↑ Synaptic plasticity, neurotransmission, memory formation, neuroprotection	[271,272]
Glucose	Fruits, vegetables, and Honey	saccharides	Neuronal energy metabolism	↑ Glycosylation, metabolic sensing, neuronal activity	[273]
Iron	Legumes, Nuts and seeds, dried fruits, red meats	Mineral	Oxidative stress regulation	ROS balance, ferroptosis modulation	[274,275]
Omega-3 Fatty Acids	A-linolenic acid (ALA), Docosahexaenoic acid (DHA)	Fatty acid	Neurodevelopment, anti-inflammatory effects	↑ Neurogenesis, synaptic plasticity, ↓ neuroinflammation	[276]
Vitamins	B1, B2, B3, Folic acid, Choline	Vitamins	Neuroprotection, cognition	↓ oxidative stress, ↑ mitochondrial function, Aβ clearance	[277,278]
Hormones	DHEA, pregnenolone, vasopressin	Endogenous hormones	Cognitive regulation, stress response	Neuroendocrine modulation via receptor signaling	[279,280]
**B. Isolated Phytochemicals.**					
Antixidants	Flavonoids, anthocyanins, phenolics, tannins	Polyphenols	Neuroprotection	↓ Oxidative stress, anti-inflammatory effects	[281,282,283,284]
DMAE and cholinergic compounds	DMAE, ALCAR, phosphatidylserine, L-theanine	Amino alcohols	Cognitive enhancement	↑ Cholinergic signaling, immune modulation	[285,286]
Caffeine compounds	Caffeine, polyphenols	Alkaloid	Cognitive stimulation	↑ Dopamine release, synaptic activity	[287,288]
Phospholipid derivatives	Phosphatidylcholine, phosphatidylserine	Lipids	Brain membrane function	↓ Microglial activation, signaling regulation	[289,290]
**C. Whole Herbal Extracts/Medicinal Plants.**					
Amla (*Emblica officinalis*)	Vitamin C, flavonoids	Phyllanthaceae	Antioxidant, neuroprotective	↑ Acetylcholine, ↓ Oxidative stress	[291,292]
*Bacopa monnieri* (*Brahmi*)	Bacosides	Plantaginaceae	Memory enhancement	↑ Acetylcholine synthesis, ↓ Aβ aggregation	[293,294]
*Curcuma longa*	Curcuminoids	Zingiberaceae	Neuroprotection	↑ BDNF, ↓ neuroinflammation	[295,296,297,298,299]
*Ginkgo biloba*	Flavonoids, bilobalide	Ginkgoaceae	Cognitive enhancement	↑ Mitochondrial function, ↓ Aβ	[300,301]
*Ginseng* (*Panax ginseng*)	Ginsenosides	Araliaceae	Neuroprotection	↑ Synaptic plasticity, ↓ AChE	[302]
*Ginger* (*Zingiber officinale*)	Gingerols	Zingiberaceae	Anti-inflammatory	↓ oxidative stress	[303,304,305]
*Gotu kola* (*Centella asiatica*)	Triterpenoids	Apiaceae	Memory enhancement	↓ NF-κB, ↑ neuroprotection genes	[306,307,308]
*Green tea* (*Camellia sinensis*)	EGCG	Theaceae	Neuroprotection	↓ oxidative stress	[298,309,310]
*Guduchi* (*Tinospora cordifolia*)	Alkaloids	Menispermaceae	Immunomodulation	↑ Neurotransmitters	[311,312,313]
*Moringa oleifera*	Polyphenols	Moringaceae	Neuroprotection	↓ ROS, ↑ AChE inhibition	[314,315,316]
*Magnolia officinalis*	Magnolol, honokiol	Magnoliaceae	Anti-stress	↓ apoptosis, ↑ cholinergic activity	[317,318]
*Ashwagandha* (*Withania somnifera*)	Withanolides	Solanaceae	Cognitive enhancement	↓ oxidative stress, ↓ Aβ aggregation	[319,320]
**D. Clinically Validated Interventions (Human Trials).**					
Ashwagandha	Withanolides	RCT	Improve memory, attention	↓ stress, ↑ synaptic plasticity	[321,322]
Bacopa monnieri	Bacosides	Clinical trial	Improve cognition	↑ cholinergic function	[323]
Ginkgo biloba (EGb 761)	Flavonoids, terpenes	RCTs	Improve memory	↑ cerebral blood flow	[324,325]
Spearmint extract	Polyphenols	RCT	Improve working memory	Neuromodulation	[326,327]
Diosgenin yam extract	Diosgenin	Human study	Cognitive improvement	Neuroplasticity enhancement	[328,329]

Abbreviation: ↓, downregulation; ↑, upregulation.

To improve clarity and scientific organization, Table 3 has been reorganized into four distinct categories: (1) nutrients and endogenous compounds, (2) isolated phytochemicals, (3) whole herbal extracts/plants, and (4) clinically validated interventions based on human studies.

#### 5.2.1. Mitochondrial Dysfunction and Cellular Energetics in Neurodegeneration

Mitochondrial dysfunction is a central mechanism underlying neurodegeneration and cognitive decline in AD [80]. Neurons require a continuous supply of ATP to maintain synaptic transmission, calcium homeostasis, membrane excitability, and cellular survival. Impaired oxidative phosphorylation reduces ATP production, disrupts neuronal metabolism, and contributes to synaptic dysfunction, oxidative stress, and neuronal death [80,330]. Excessive production of ROS from damaged mitochondria further promotes lipid, protein, and DNA damage, thereby aggravating β-amyloid toxicity and neurodegeneration. In AD, mitochondrial abnormalities such as electron transport chain impairment, mitochondrial DNA damage, defective calcium buffering, and elevated oxidative stress are strongly associated with progressive cognitive decline [331,332]. Mitochondrial homeostasis is maintained through coordinated regulation of mitochondrial biogenesis, energy metabolism, and quality-control pathways. The AMPK/SIRT1/PGC-1α signaling axis plays an important role in this process. AMPK functions as a cellular energy sensor during metabolic stress, whereas SIRT1 activates PGC-1α through deacetylation. Activated PGC-1α promotes mitochondrial biogenesis, oxidative phosphorylation, and antioxidant defense mechanisms [333]. Dysregulation of these pathways contributes to impaired mitochondrial function in neurodegenerative disorders.

Mitophagy, the selective removal of damaged mitochondria, is another essential mechanism for maintaining neuronal homeostasis. Defects in mitophagy-related pathways, including PINK1/Parkin signaling, result in the accumulation of dysfunctional mitochondria and increased oxidative stress within neurons [334,335]. Several herbal compounds discussed in this review have demonstrated beneficial effects on mitochondrial function and cellular energetics. Resveratrol activates the SIRT1/AMPK/PGC-1α pathway, thereby promoting mitochondrial biogenesis, improving ATP production, and reducing oxidative stress [336]. Curcumin suppresses mitochondrial ROS generation, stabilizes mitochondrial membrane potential, and attenuates oxidative damage and neuroinflammation [337]. Curcumin has also demonstrated regulatory effects on intracellular stress-response pathways beyond neurological models. For example, curcumin-based combination treatment has been reported to induce apoptosis through mitochondrial-mediated intrinsic signaling and ATR/ATM/p53-dependent pathways, highlighting its ability to modulate redox-sensitive molecular networks involved in cellular survival and degeneration [338]. These mechanistic findings further support the broader therapeutic potential of curcumin as a multitarget phytochemical with possible neuroprotective relevance. Similarly, evidence from non-neuronal cellular models has shown that Atractylodes lancea exerts anticancer activity by regulating ROS-mediated apoptotic pathways, further emphasizing the importance of redox homeostasis and oxidative stress modulation in cellular survival and degeneration [339]. Ginseng and its ginsenosides enhance mitochondrial energy metabolism, preserve electron transport chain activity, and strengthen antioxidant defenses in neuronal tissues. Similarly, Bacopa monnieri has been reported to protect mitochondrial integrity, reduce lipid peroxidation and ROS accumulation, and improve endogenous antioxidant status, thereby supporting neuronal survival and cognitive function [340,341]. Collectively, these findings indicate that modulation of mitochondrial dysfunction and cellular energetics represents an important mechanism through which herbal medicines may attenuate neurodegenerative progression and preserve cognitive health.

#### 5.2.2. Epigenetic and Gene-Regulatory Mechanisms of Herbal Medicines in Neurodegeneration

Recent studies suggest that epigenetic dysregulation contributes significantly to the progression of neurodegenerative disorders, including AD. Epigenetic mechanisms such as histone acetylation and deacetylation, DNA methylation, and microRNA (miRNA) regulation influence synaptic plasticity, neuronal survival, memory formation, and neuroinflammation [342]. Increased histone deacetylase (HDAC) activity has been associated with cognitive impairment and enhanced neuroinflammatory responses, whereas phytochemicals such as curcumin and resveratrol may promote neuroprotective gene expression through modulation of histone acetylation pathways [343,344]. Abnormal DNA methylation patterns have also been linked to β-amyloid accumulation and neuronal dysfunction in AD [345]. MicroRNAs, including miR-124, miR-132, miR-146a, and miR-155, are important regulators of neuroinflammation and synaptic signaling [346]. Dysregulation of these miRNAs contributes to microglial activation, oxidative stress, and cognitive decline in neurodegenerative disorders [347].

The Nrf2/Keap1 signaling pathway also plays a major role in cellular antioxidant defense through the regulation of oxidative stress-responsive genes. Several herbal compounds, including curcumin, resveratrol, ginsenosides, and *Bacopa monnieri*, have demonstrated the ability to activate Nrf2 signaling, suppress oxidative stress, and reduce neuroinflammation [341,348,349]. SIRT1-mediated neuroprotection is increasingly recognized as an important therapeutic mechanism in aging and neurodegeneration. SIRT1 regulates histone deacetylation, mitochondrial biogenesis, inflammatory signaling, and neuronal survival pathways. Activation of SIRT1 has been associated with reduced neuronal apoptosis, improved synaptic plasticity, and attenuation of β-amyloid-induced toxicity [350,351,352]. Resveratrol, in particular, has shown neuroprotective effects through activation of SIRT1 signaling and reduction in oxidative and inflammatory damage [353]. Epigenetic modulation and gene-regulatory processes collectively represent promising therapeutic avenues through which herbal medicines may counteract neurodegenerative progression and promote brain health.

#### 5.2.3. Herbal Medicines as Modulators of Gut Microbiota and Their Impact on Brain Function

Herbal medicines can indirectly influence brain function by modulating gut microbiota composition and gut–brain communication pathways. Several phytochemicals, including curcumin, resveratrol, ginsenosides, and *Bacopa monnieri*, act as natural prebiotic-like compounds that promote beneficial bacteria while suppressing harmful microbial populations. These effects help maintain gut homeostasis, metabolic balance, and gut–brain axis function [261]. Short-chain fatty acids (SCFAs), particularly butyrate, acetate, and propionate, are produced through microbial fermentation of herbal polysaccharides and dietary compounds. These metabolites exert neuroprotective effects by reducing neuroinflammation, strengthening BBB integrity, regulating microglial activation, and supporting neuronal energy metabolism. Butyrate has also been associated with the regulation of gene expression involved in synaptic plasticity and cognitive function [354].

Herbal-induced alterations in gut microbiota may additionally influence vagus nerve signaling, an important bidirectional communication pathway between the gut and the brain. Through this pathway, gut-derived signals can modulate neuroimmune responses, stress signaling, and neuronal activity. Tryptophan metabolism is another major gut–brain pathway influenced by intestinal microbiota. Microbial regulation of serotonin biosynthesis and kynurenine pathway metabolites may affect mood, cognition, and neuroinflammatory processes. By restoring microbial balance, herbal medicines may help improve serotonergic signaling and cognitive health [355]. Gut dysbiosis can also increase intestinal permeability, leaky gut, allowing translocation of lipopolysaccharides and other inflammatory endotoxins into systemic circulation. This process promotes systemic inflammation and neuroinflammatory responses associated with cognitive decline. Herbal medicines may help restore intestinal barrier integrity, reduce endotoxin translocation, and attenuate inflammation-mediated neuronal dysfunction [356]. The following Table 4 summarizes representative herbal compounds and their modulatory effects on gut microbiota composition and associated neuroprotective mechanisms via the gut–brain axis.

#### 5.2.4. Proteostasis and Autophagy-Regulating Mechanisms of Herbal Medicines in Neurodegeneration

Protein homeostasis (proteostasis) is essential for maintaining neuronal integrity and normal cellular function. Disruption of proteostasis contributes to the accumulation of misfolded and toxic proteins, including amyloid-β (Aβ) plaques and hyperphosphorylated tau aggregates, which are characteristic features of neurodegenerative disorders [367]. Cellular protein quality control is primarily regulated by the autophagy–lysosomal pathway and the ubiquitin–proteasome system, both of which are responsible for the degradation and clearance of damaged proteins and dysfunctional cellular components [368,369]. Impairment of these systems promotes defective protein clearance, oxidative stress, synaptic dysfunction, and progressive neuronal degeneration. While the UPS mainly degrades ubiquitinated proteins, autophagy plays a critical role in the lysosomal removal of aggregated proteins [370]. Dysregulation of these pathways has been strongly associated with AD pathology and cognitive decline.

The mammalian target of rapamycin (mTOR) signaling pathway is a major regulator of autophagy and cellular metabolism. Hyperactivation of mTOR suppresses autophagic activity and contributes to the accumulation of Aβ and tau pathology in neurodegenerative diseases [371]. Several herbal compounds have demonstrated neuroprotective effects through modulation of autophagy-related signaling pathways [367]. Resveratrol and curcumin have been reported to enhance autophagy and promote clearance of toxic protein aggregates through inhibition of mTOR signaling and activation of AMPK-related pathways [372]. Ashwagandha also exhibits neuroprotective effects by reducing oxidative stress, enhancing protein clearance mechanisms, and decreasing neuronal apoptosis. In addition, maca has shown antioxidant and neuroprotective properties associated with the regulation of cellular stress responses and maintenance of neuronal homeostasis [373]. All of these results point to the possibility that herbal remedies may slow the course of neurodegenerative diseases and maintain cognitive function by modifying proteostasis and autophagy-related pathways. It is becoming increasingly acknowledged that maintaining proteostasis and restoring autophagic flux are effective treatment approaches for slowing the progression of cognitive disorders [374]. Therefore, herbal compounds capable of regulating autophagy-related pathways and enhancing protein clearance mechanisms may offer significant potential for maintaining neuronal integrity during neurodegenerative aging.

#### 5.2.5. Synaptic Plasticity and Neurotrophic Mechanisms of Herbal Medicines

Synaptic plasticity is essential for learning, memory formation, and cognitive flexibility. Impairment of synaptic plasticity is strongly associated with cognitive disorders and neurodegenerative diseases [375]. Neurotrophic signaling pathways, particularly brain-derived neurotrophic factor (BDNF) and its receptor tropomyosin receptor kinase B (TrkB), play important roles in neuronal survival, synaptic transmission, and long-term potentiation (LTP) [376]. Activation of BDNF/TrkB signaling stimulates pathways involved in neuronal growth, synaptic strengthening, and memory consolidation [377]. The cAMP response element-binding protein (CREB) is another important transcription factor involved in neuronal plasticity and memory-related gene expression. Reduced BDNF and CREB activity has been associated with synaptic dysfunction, impaired LTP, and cognitive decline in neurodegenerative disorders [377,378]. Several herbal compounds have demonstrated neuroprotective effects through modulation of neurotrophic and synaptic signaling pathways. Herbal extracts have been reported to enhance BDNF expression, activate CREB signaling, and improve synaptic plasticity in experimental models [379]. In addition, phytochemicals may promote synaptogenesis, preserve dendritic spine density, and strengthen neuronal connectivity, thereby supporting cognition and memory function [380]. Supporting evidence from related cellular models has demonstrated activation of p38/FOXO1 and PI3K/AKT signaling pathways, further emphasizing the broader role of these conserved pathways in cellular survival, regeneration, and neuroprotective regulation [381]. Enhancement of synaptic architecture and restoration of LTP are increasingly recognized as important therapeutic strategies for delaying cognitive decline [382]. Therefore, phytochemicals capable of modulating neurotrophic signaling and synaptic remodeling pathways may offer multi-target therapeutic potential for preserving neuronal resilience, memory function, and healthy brain aging [383].

#### 5.2.6. Blood–Brain Barrier Permeability and CNS Bioavailability of Phytochemicals

The ability of herbal phytochemicals to penetrate the BBB and attain adequate CNS bioavailability is crucial to their therapeutic effectiveness in neurodegenerative diseases. The BBB is a highly selective neurovascular interface composed of endothelial cells, astrocytes, pericytes, and tight junction proteins that regulate molecular transport into the brain [384]. BBB dysfunction has been associated with increased neuroinflammation, oxidative stress, impaired amyloid-β clearance, and neuronal degeneration in AD and related disorders. Several phytochemicals, including curcumin, ginsenosides, bacosides, epigallocatechin gallate (EGCG), and resveratrol, have demonstrated the ability to cross the BBB through passive diffusion, carrier-mediated transport, or lipid-associated mechanisms [385,386]. In addition to their neuroprotective effects, these compounds may help preserve BBB integrity by modulating tight junction proteins such as occludin, claudin-5, and zonula occludens-1 (ZO-1), thereby reducing endothelial dysfunction and neuroinflammatory signalling [387,388]. Curcumin and resveratrol have been reported to attenuate BBB disruption through antioxidant and anti-inflammatory mechanisms, whereas ginsenosides and bacosides may improve cerebral circulation and neuronal signalling. EGCG has also shown protective effects against amyloid-induced vascular injury and endothelial dysfunction [389].

Despite their therapeutic potential, many phytochemicals exhibit poor oral bioavailability and limited BBB permeability due to rapid metabolism and low solubility [390]. To overcome these limitations, advanced drug-delivery systems such as nanoparticles, liposomes, nanoemulsions, and polymer-based carriers have been increasingly explored to enhance CNS targeting and improve pharmacokinetic stability [391]. In experimental models, nanoformulated phytochemicals demonstrated improved BBB penetration, prolonged circulation time, and enhanced neuroprotective efficacy [392]. These nanotechnology-assisted approaches may therefore improve the translational potential of herbal neurotherapeutics for cognitive and neurodegenerative disorders.

### 5.3. Herbal Medicine for the Prevention and Treatment of Alzheimer’s Disease

The recent failure of medications aimed at tau deposits raises questions about comprehension of the intricate biology of AD [393]. This emphasizes the necessity to investigate additional pathophysiological factors, including autophagic dysfunction, chronic neuroinflammation, oxidative damage, metal ion dyshomeostasis, excitotoxic neurotransmission, gut microbiota imbalance, endoplasmic reticulum stress, altered cholesterol metabolism, metabolic regulatory dysfunction of insulin or glucose, and pathogenic infections, that contribute to AD [394,395]. Despite their prolonged heritage of therapeutic practice and apparent safety and effectiveness, herbs and herbal medicines have sadly received little scientific research [396]. Traditional medical practices propose a variety of plants and their components to improve neurocognitive performance and relieve auxiliary signs of AD, such as depression, amnesia, and poor cognition. The recommendation for plant-based amalgamation usually depends on how complicated the problem is. The reasoning behind this is that the herb’s bioactive components may both operate in concert with one another and modify the actions of other components derived from the same or different plant species [396]. The reasons for selecting bioactive botanicals are: (a) their enduring role in folk medicine for treating memory dysfunction, including AD, (b) assessment of plant-derived nutraceuticals composition from these plants with potential therapeutic benefits for AD, (c) the evaluation of their neuropharmacological activities, and (d) laboratory and patient-based studies provide converging evidence for their neuroprotective and memory-enhancing properties. Herbal medicine has emerged as a promising complementary approach for the management of Alzheimer’s disease. Certain phytochemicals derived from medicinal plants may improve cognitive function through antioxidant, anti-inflammatory, and neuroprotective mechanisms. Plants rich in antioxidants like flavonoids, beta-carotene, vitamin C, and vitamin E may help mitigate neurodegenerative symptoms by addressing oxidative stress, which is scientifically linked to the advancement of Alzheimer’s pathology [397,398].

### 5.4. Clinical Applications of Plant-Based Interventions for Alzheimer’s Management

Although the precise biochemical mechanisms underlying AD remain incompletely understood, it is widely accepted that the disorder is multifactorial and that aging is the strongest risk factor. Despite more than fifty therapeutic candidates successfully completing phase II clinical trials over the past decade, none have progressed to phase III trials [399]. Several herbal medicines and botanical extracts have been clinically evaluated in humans for their potential cognitive benefits relevant to AD and mild cognitive impairment (MCI).

Clinical studies investigating diosgenin-rich yam extract demonstrated that oral supplementation produced measurable cognitive improvements in healthy adults aged 20–81 years. Notably, older participants (≥47 years old) showed significant enhancement in the Repeatable Battery for the Assessment of Neuropsychological Status (RBANS) total score, marking the first evidence that diosgenin-containing extracts can improve human cognitive performance [400]. Similarly, an ethanolic extract of spearmint (*Mentha spicata* L.) was evaluated in a randomized, double-blind, placebo-controlled trial involving adults with age-associated memory impairment. Across 90 days of supplementation, participants receiving 900 mg/day exhibited significant improvements in working memory quality and spatial working memory compared with placebo, as assessed using the cognitive drug research system. Additional benefits included improved vigor-activity scores and enhanced subjective sleep quality, suggesting broader neuro-modulatory effects beyond cognitive enhancement [325].

A randomized placebo-controlled trial examining *Salicornia europaea* L. ethanol extract (PM-EE) further supported the cognitive benefits of herbal interventions. After accounting for study withdrawals and compliance issues, data from 53 participants revealed that PM-EE supplementation resulted in greater improvements in cognitive performance than placebo in individuals with memory impairment but without dementia, demonstrating both safety and clinical potential [401]. Clinical evaluation of Ashwagandha (*Withania somnifera* (L.) Dunal) in patients seeking treatment for MCI also yielded promising outcomes. In a prospective, randomized, double-blind, placebo-controlled pilot trial involving 50 participants, Ashwagandha root extract significantly enhanced attention, executive function, immediate and general memory, visuospatial skills, and information-processing speed. These findings indicate that Ashwagandha may offer cognitive support for individuals with MCI while presenting minimal risk [402].

Finally, the cognitive effects of *Zanthoxylum armatum DC*. The purified compound was assessed using a double-blind, parallel-group, placebo-controlled design evaluating both acute and chronic responses. Across assessments conducted pre-dose and at 1, 3, and 5 h post-dose on day 1, as well as after 56 days of supplementation, the hydroxy-α-sanshool-rich–rich extract produced consistent enhancements in cognitive task performance, primarily reflected in improved cognitive processing speed. In an optional cerebral blood flow sub-study, the extract significantly improved CBF and reduced frontal-cortex hemodynamic responses during cognitively demanding tasks, suggesting increased neural efficiency and improved cerebrovascular function [403].

### 5.5. Limitations of Clinical or Preclinical Trial

Although herbal medicines have shown significant promise in modulating cognitive functions and slowing neurodegenerative processes, several key limitations remain in both clinical and preclinical studies. Preclinical research has demonstrated that numerous phytochemicals, including phenolic compounds, flavonoids, terpenoids, and alkaloids, possess neuroprotective potential through their antioxidant activity, anti-inflammatory effects, preservation of mitochondrial function, and modulation of neurotransmitter systems [126,267]. However, the translation of these findings into clinical effectiveness remains challenging. Major issues include small sample sizes in clinical trials, short study durations, variability in herbal preparation methods, lack of standardized dosages, and inconsistencies in outcome measures. These limitations restrict the reproducibility, reliability, and generalizability of current findings and highlight the need for well-designed trials and long-term follow-up assessments [404].

**Figure 4:**
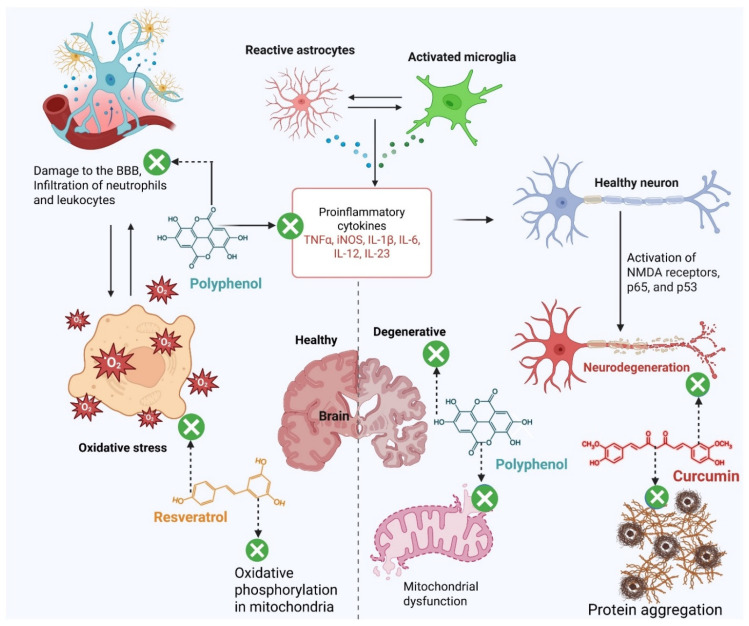
Herbal bioactive compounds counteract major neurodegenerative mechanisms. Neurodegenerative diseases involve interconnected mechanisms including chronic neuroinflammation, oxidative stress, mitochondrial dysfunction, β-amyloid/tau aggregation, synaptic damage, and progressive blood–brain barrier (BBB) disruption. The figure illustrates how three major phytochemicals, such as curcumin, resveratrol, and polyphenols, modulate these pathways to reduce neuronal damage [389,410]. Despite these promising mechanisms, clinical translation remains limited by bioavailability challenges, variability in formulations, and insufficient high-quality clinical evidence [408].

The interconnected mechanisms underlying neurodegeneration, including oxidative stress, mitochondrial dysfunction, neuroinflammation, disruptions in the BBB, synaptic deterioration, and β-amyloid/tau aggregation, are summarized in Figure 4 [405]. This figure also illustrates how key phytochemicals such as curcumin, resveratrol, and polyphenols counteract these pathogenic pathways through antioxidant activity, suppression of inflammatory cascades, inhibition of Aβ/tau aggregation, enhancement of mitochondrial function, and stabilization of neuronal integrity [406,407]. These mechanistic insights provide a strong rationale for exploring herbal compounds in the management of cognitive decline; however, the clinical translation of these mechanisms remains limited due to poor bioavailability, extensive first-pass metabolism, rapid clearance, and inconsistent therapeutic concentrations in the central nervous system [408,409].

Beyond these mechanistic limitations, substantial heterogeneity exists in clinical studies regarding patient demographics, cognitive assessment tools, and treatment durations [262]. Many herbal interventions are studied as multi-component formulations, making it difficult to attribute benefits to specific bioactive molecules [411]. In addition, variations in extraction methods, plant part selection, cultivation conditions, and phytochemical composition contribute to inconsistencies between studies and reduce reproducibility [412]. Several studies also lack placebo controls or double-blind designs, limiting the strength and reliability of the clinical evidence [413]. Pharmacokinetic limitations remain another major challenge in translating herbal compounds into clinical applications. Bioactive constituents such as curcumin, ginsenosides, bacosides, and resveratrol often exhibit poor solubility, low gastrointestinal absorption, rapid metabolism, and limited BBB penetration, resulting in reduced bioavailability and inconsistent therapeutic efficacy [414]. Nanotechnology-based delivery systems, including liposomes, phytosomes, nano-emulsions, and polymeric nanoparticles, have shown promise in enhancing bioavailability, improving BBB penetration, and increasing therapeutic effectiveness, although these approaches remain underrepresented in clinical trials [415]. In addition to methodological limitations, safety concerns, herb–drug interactions, and standardization issues remain major barriers to the clinical translation of herbal medicines.

#### 5.5.1. Herb–Drug Interactions and Standardization Challenges

Safety, herb–drug interactions, and standardization issues also require careful consideration. Herbal compounds may interact with medications commonly prescribed to elderly patients, including cholinesterase inhibitors, antidepressants, anticoagulants, and antiplatelet agents [416]. For example, Ginkgo biloba has antiplatelet activity that may increase bleeding risk when administered alongside aspirin or warfarin [417]. Several phytochemicals may also modulate cytochrome P450 enzymes, including CYP3A4, CYP2D6, and CYP2C9, thereby altering drug metabolism, therapeutic activity, and toxicity profiles [418]. Such interactions may increase the risk of adverse effects, particularly in patients with multiple comorbidities and polypharmacy [419].

Another major challenge is the lack of standardization and quality control in herbal preparations. Variability in cultivation conditions, harvesting methods, extraction procedures, and concentrations of bioactive constituents may lead to inconsistent therapeutic outcomes and reduced reproducibility across clinical studies. Furthermore, concerns have been raised regarding hepatotoxicity, microbiological contamination, pesticide residues, heavy metal accumulation, and synthetic drug adulteration in certain herbal products [420]. Therefore, standardized formulations, rigorous quality control measures, and comprehensive safety evaluations are essential to improve the clinical reliability and regulatory acceptance of herbal medicines. Future studies should incorporate pharmacokinetic, toxicological, and herb–drug interaction assessments to support the safe integration of herbal therapies into evidence-based management strategies for neurodegenerative diseases. These challenges highlight the importance of rigorous pharmacovigilance systems, standardized manufacturing procedures, toxicological evaluation, and herb–drug interaction studies to improve the clinical reliability and translational applicability of herbal therapies.

Despite these limitations, increasing scientific evidence supports the therapeutic potential of medicinal plants and phytochemicals in modulating neuroinflammation, oxidative stress, mitochondrial dysfunction, and amyloid/tau pathology associated with AD [421,422,423]. Several plant-derived metabolites have already progressed to commercialization and clinical use [424]. The following sections summarize selected medicinal plants and herbal compounds investigated for their neuroprotective and cognitive-enhancing effects in AD.

#### 5.5.2. Ashwagandha (*Withania somnifera*)

*Withania somnifera* has demonstrated neuroprotective potential in several neurodegenerative disorders, including AD, PD, and Huntington’s disease [425]. Its therapeutic effects are associated with modulation of oxidative stress markers such as glutathione (GSH), catalase, lipid peroxidation, and superoxide dismutase (SOD), together with promotion of axonal and dendritic regeneration [426]. Withaferin-A, one of the major active components of Ashwagandha, has shown promise in AD. It reduces the aggregation of β-amyloid and tau proteins, helps regulate heat shock protein production, and suppresses oxidative and pro-inflammatory pathways [427]. Experimental studies further demonstrated that Ashwagandha improves spatial memory, enhances synaptic plasticity markers, and reduces phosphorylated NF-κB levels, indicating anti-inflammatory activity [428]. Clinical evidence suggests that supplementation with 500–600 mg/day for 8–12 weeks may improve memory, executive function, sustained attention, and processing speed in individuals with mild cognitive impairment and early-stage dementia [429].

#### 5.5.3. Brahmi (*Bacopa monnieri*)

*Bacopa monnieri* is widely recognized for its nootropic and memory-enhancing properties [430]. Its neuroprotective activity is mainly attributed to bacosides, alkaloids, and other secondary metabolites that support synaptic activity, neurotransmission, and neuronal repair [431]. Experimental studies indicate that Brahmi reduces oxidative stress, enhances cholinergic activity, and improves learning and memory performance [432]. Clinical investigations have shown that *Bacopa monnieri* supplementation improves cognitive performance, including executive function, attention, delayed recall, verbal fluency, and visuospatial processing [433]. In addition, treatment with Brahmi extract reduced oxidative stress markers such as hydroperoxides, malondialdehyde, and ROS while restoring cholinergic enzyme activity, suggesting potential protective effects against neurodegeneration [434].

#### 5.5.4. Cat’s Claw (*Uncaria tomentosa*)

*Uncaria tomentosa* has attracted attention as a potential therapeutic candidate for AD due to its anti-inflammatory and neuroprotective activities [435]. Polyphenolic compounds, particularly proanthocyanidin B2, have demonstrated the ability to reduce Aβ plaque burden, gliosis, astrocytosis, and neuroinflammation in transgenic AD mouse models while improving short-term memory performance [435]. Additional evidence suggests that cat’s claw extracts inhibit oxidative stress and modulate pathways associated with neurodegeneration. Although preclinical findings are promising, further clinical studies are required to validate its efficacy and long-term safety in AD management [436].

#### 5.5.5. *Moringa olifera*

*Moringa oleifera* exhibits significant antioxidant and neuroprotective properties relevant to AD management [437,438]. Experimental studies indicate that its extracts reduce reactive oxygen species production, protect neuronal tissue from oxidative damage, and enhance cholinergic signaling through inhibition of acetylcholinesterase activity [439]. Recent studies also suggest that *M. oleifera* phytochemicals modulate AD-related mechanisms involving neuroinflammation, tau pathology, and β-amyloid accumulation [440]. Glucomoringin, one of its major bioactive compounds, has demonstrated anti-inflammatory and neuroprotective activity in experimental models, although additional clinical studies are required to confirm its therapeutic efficacy and long-term safety [441].

#### 5.5.6. *Ginseng*

*Ginseng* contains several neuroactive constituents, including ginsenosides, polysaccharides, and gintonin, which contribute to its cognitive-enhancing and neuroprotective properties [442,443]. Experimental studies have demonstrated that ginseng extracts reduce neuroinflammation, improve cholinergic signaling, and enhance synaptic plasticity in neurodegenerative disease models [444]. Clinical evidence suggests that short-term and long-term supplementation with ginseng extract may improve working memory and cognitive performance in healthy individuals and patients with early AD [442,443]. However, further well-designed clinical studies are necessary to determine optimal dosage, formulation standardization, treatment duration, and long-term safety [445].

#### 5.5.7. *Ginkgo biloba*

*Ginkgo biloba* contains several bioactive compounds, particularly flavonoids and terpene trilactones, which contribute to its neuroprotective and cognition-enhancing properties [301]. The standardized extract EGb 761 has been extensively investigated for improving cerebral blood flow, reducing oxidative stress, decreasing amyloid-β accumulation, and enhancing neuronal metabolism [446,447,448]. Clinical studies in patients with AD and dementia demonstrated modest improvements in cognitive performance, including MMSE, ADAS-Cog, and CGIC scores, following EGb 761 supplementation [449]. However, additional large-scale and long-term clinical trials are required to confirm its therapeutic efficacy, optimal dosage, and long-term safety in neurodegenerative disorders [450].

#### 5.5.8. *Glycyrrhiza glabra*

*Glycyrrhiza glabra* (licorice) contains several bioactive compounds, including glycyrrhizin, liquiritigenin, licochalcone A, and flavonoids, which exhibit antioxidant, anti-inflammatory, and neuroprotective activities [451,452]. Experimental studies demonstrated that licorice extracts improve learning and memory by enhancing cholinergic neurotransmission, reducing oxidative stress, and suppressing amyloid-β aggregation [453]. Additional studies reported that Glycyrrhiza inflata extracts reduced mitochondrial dysfunction and oxidative damage while improving neuronal survival [454]. These findings suggest that Glycyrrhiza species may provide neuroprotective benefits in AD through modulation of oxidative stress and neuroinflammatory pathways.

#### 5.5.9. Curcuma Plants

*Curcuma longa* and its major active compound curcumin have attracted substantial interest because of their antioxidant, anti-inflammatory, and anti-amyloidogenic properties [455]. Experimental studies demonstrated that curcumin reduces oxidative stress, suppresses neuroinflammation, inhibits acetylcholinesterase activity, and protects against neuronal degeneration in multiple AD-related models [456,457,458,459]. Curcumin has also been shown to decrease amyloid-β aggregation and improve cognitive performance in animal studies [460]. Despite promising neuroprotective effects, its clinical application remains limited due to poor bioavailability and rapid metabolism.

#### 5.5.10. *Pistachio vera*

*Pistacia vera* contains several neuroprotective phytochemicals, including flavonoids, resveratrol, lutein, anthocyanins, and unsaturated fatty acids [461]. Oleic acid and linolenic acid present in pistachio have been associated with antioxidant activity, neuroprotection, and improved neuronal membrane function [462,463,464]. β-sitosterol, one of its major bioactive compounds, demonstrated protective effects against cognitive and motor impairments induced by neurotoxic agents [465]. These findings suggest that Pistacia vera may contribute to cognitive protection through antioxidant and anti-inflammatory mechanisms.

#### 5.5.11. *Phyllanthus acidus*

*Phyllanthus acidus* possesses polyphenolic compounds with antioxidant and cholinesterase inhibitory activities relevant to AD management [466]. Experimental studies demonstrated that extracts from Phyllanthus species inhibit AChE and BuChE, thereby enhancing cholinergic neurotransmission [466]. The plant also exhibits antioxidant and anti-inflammatory properties that may help reduce neurodegenerative damage associated with AD [467]. However, evidence supporting its clinical efficacy remains limited, and additional mechanistic and human studies are necessary.

#### 5.5.12. Emerging Herbal Candidates for Cognitive Protection

*Melissa officinalis* has demonstrated anxiolytic, antioxidant, and cholinergic modulatory activities that may improve memory and cognitive performance [468]. Clinical studies suggest that lemon balm extract may reduce agitation and cognitive decline in patients with mild to moderate AD through regulation of cholinergic neurotransmission and oxidative stress pathways [469]. *Salvia officinalis* and *Rosmarinus officinalis* have also shown promising neuroprotective effects due to their rich polyphenolic and terpenoid content. Experimental and clinical studies indicate that these herbs exhibit antioxidant, anti-inflammatory, and acetylcholinesterase inhibitory activities, which may improve working memory, attention, and cognitive processing speed while reducing neuronal oxidative damage [470]. Hericium erinaceus (lion’s mane mushroom) has attracted increasing attention because of its ability to stimulate nerve growth factor synthesis and support neuronal regeneration. Experimental and clinical evidence suggests that supplementation with H. erinaceus may enhance neuroplasticity, reduce neuroinflammation, and improve cognitive function [471,472]. *Crocus sativus* (saffron) has also demonstrated therapeutic potential in AD through antioxidant, anti-inflammatory, and anti-amyloidogenic mechanisms [473]. Clinical studies reported that saffron supplementation improved cognitive performance and exhibited efficacy comparable to conventional anti-Alzheimer’s medications, with fewer adverse effects [474]. Collectively, these emerging herbal candidates further support the therapeutic potential of plant-derived compounds in neurodegenerative disorders through multi-target modulation of oxidative stress, neuroinflammation, synaptic plasticity, and neurotransmitter regulation. The cognitive and metabolic effects, therapeutic dosages, and proposed mechanisms of selected traditional medicinal extracts reported in clinical and experimental studies are summarized in Table 5.

## 6. Conventional Herbal Medicine Treatment for Moderate Cognitive Impairment and Early-Stage AD Based on Syndrome Differentiation

Herbal medicine has demonstrated promising therapeutic potential in improving cognitive function in patients with AD and MCI. Several studies have reported improvements in widely used cognitive assessment tools, including the Mini-Mental State Examination (MMSE), ADAS-Cog, and Montreal Cognitive Assessment (MoCA), following herbal medicine interventions [484]. Herbal therapies have also shown benefits in MCI, which is considered a prodromal stage preceding dementia and currently lacks fully effective pharmacological treatment options [485]. However, considerable variability exists among clinical studies due to differences in herbal formulations, treatment duration, and syndrome differentiation (SD)-based therapeutic approaches used in TCM. Therefore, understanding SD is important for interpreting the rationale, personalization, and clinical outcomes of herbal medicine interventions in cognitive impairment and early-stage AD.

### 6.1. Overview of Syndrome Differentiation in Cognitive Impairment and AD

Syndrome differentiation (SD), also known as pattern identification, is a core diagnostic principle in TCM that classifies patients according to characteristic symptom patterns, functional disturbances, and underlying pathophysiological imbalances rather than relying solely on a single disease diagnosis [486]. In cognitive impairment and AD, different SD patterns, including kidney deficiency, phlegm obstruction, blood stasis, qi stagnation, and heart–spleen deficiency, are believed to reflect distinct pathological and clinical states associated with disease progression [487]. Unlike conventional approaches that apply a uniform treatment strategy, SD-guided therapy aims to personalize herbal interventions according to the patient’s specific syndrome pattern and symptom presentation. This individualized approach may support multi-target therapeutic modulation involving neuroinflammation, oxidative stress, neurotransmitter imbalance, and cerebral circulation. Therefore, SD-based herbal medicine represents a personalized therapeutic framework that may improve clinical management strategies for mild cognitive impairment (MCI) and early-stage AD.

The efficacy of herbal medicine prescriptions has been evaluated in patients with MCI according to SD patterns such as hepatic yang hyperactivity, renal insufficiency, and qi deficiency [488]. Previous studies in elderly patients with MCI reported deficiency syndromes involving the kidney system, spleen qi, and heart blood, together with excess syndromes including phlegm accumulation, blood stasis, and liver qi stagnation [489]. Combined deficiency–excess patterns were also frequently observed. Clinical findings further suggest that SD-guided herbal therapy may provide greater therapeutic benefits during moderate stages of AD than in severe stages, particularly in patients with cardiac qi deficiency and depletion of renal essence, as reflected by improved MMSE and activities of daily living scores [490]. These findings support the potential role of SD-based herbal medicine as a personalized and multi-target therapeutic strategy for cognitive impairment and early-stage AD. However, additional large-scale clinical studies, longitudinal investigations, and real-world data analyses are still required to validate SD classifications and optimize individualized treatment strategies during disease progression.

### 6.2. Finding Transcriptomic Peripheral Blood Indicators and Creating Herbal Medicine Treatments for Every Stage of Cognitive Decline

Patients with AD exhibit a strong blood–brain association, where peripheral blood transcriptomic profiles reflect systemic and neuropathological alterations associated with disease progression [491]. Studies have shown that AD and MCI share several pathological mechanisms, including dysregulated immune responses, impaired energy metabolism, oxidative stress, and altered cell survival signaling [492]. Transcriptomic analyses further demonstrated progressive molecular changes during the transition from prodromal MCI to moderate and severe AD, including altered oxidative phosphorylation, ribosomal activity, and decreased ABCB1 expression, a biomarker associated with AD progression [488]. Comparative transcriptomic studies revealed that many molecular pathways altered in MCI are also dysregulated in AD dementia, supporting the concept that MCI represents an early transitional stage of neurodegeneration [493]. However, some molecular signatures differ according to disease stage. Mid-stage AD has been associated with reduced pathways related to ionic balance, cytoskeletal organization, and signal transmission, whereas inflammatory and neuronal activity pathways are increased [494]. Gene set enrichment analyses also identified alterations in synaptic and mitochondrial functions as important early pathogenic events during AD progression [495].

Recent investigations have identified several transcriptomic biomarkers associated with disease conversion and progression. Reduced interferon signaling and lower STAT1 expression were associated with increased risk of conversion from subjective cognitive decline to MCI [496,497]. Similarly, altered MS4A6A expression patterns suggest that MCI may represent a compensatory transitional phase before the development of advanced AD pathology [497]. Machine-learning approaches integrating transcriptomic, biochemical, and genetic markers such as EEF2 and RPL7 have also demonstrated promising predictive accuracy for identifying individuals at high risk of MCI and AD progression [498]. Notably, blood transcriptomic signatures predicted progression from MCI to AD with more than 70% accuracy, outperforming some radiological predictors [499]. Collectively, these findings suggest that peripheral blood transcriptomics may provide valuable biomarkers for early diagnosis, patient stratification, and personalized herbal therapeutic strategies in AD [500].

### 6.3. Finding Biomarkers for Neuroimaging and Creating a Natural Medicine Treatment for Cognitive Impairment

AD progression is associated with structural and functional brain disorganization that leads to impaired neuronal connectivity and cognitive decline [501]. Functional connectivity within the default mode network has emerged as a potential neuroimaging biomarker for AD [502]. Studies reported that prolonged Bushen capsule therapy improved connectivity in the right precuneus and other regions of the default mode network, which correlated with improvements in neuropsychological assessments [503]. Additional established neuroimaging biomarkers include FDG-PET measurements of cerebral glucose metabolism, beta-amyloid deposition, hippocampal and cortical atrophy detected by MRI, and alterations in regional cerebral blood flow (rCBF) [504,505]. Herbal interventions such as toki-shakuyaku-san significantly improved rCBF in the posterior cingulate region and enhanced positional awareness in patients with MCI and AD [506]. Similarly, Chotosan administration improved MMSE scores and reduced P300 latency, suggesting beneficial effects on cognitive processing and neuroelectrical activity [507]. Other traditional herbal formulations containing astragalus, ginseng, Pueraria, and related medicinal plants also demonstrated improvements in MMSE performance and electrophysiological parameters [508]. Furthermore, Korean red ginseng has been associated with enhanced frontal lobe activity and increased relative alpha power in AD patients [509]. Functional MRI studies of compound congrongyizhi capsule also demonstrated altered activity in the posterior cingulate, inferior frontal cortex, and lingual cortex following treatment [510].

Although neuropsychological assessments remain clinically important, neuroimaging approaches provide objective and non-invasive measurements of neural activity and treatment response. Therefore, integrating neuroimaging biomarkers into herbal medicine research may improve evaluation of therapeutic efficacy and support the development of personalized interventions for cognitive impairment and AD.

### 6.4. Gut Microbiota, SD Phenotypes, and Personalized Herbal Therapy

Recent studies have highlighted the important role of the gut–brain axis in the progression of AD and MCI. Alterations in gut microbiota composition have been associated with neuroinflammation, oxidative stress, amyloid accumulation, and cognitive decline through immune signaling and microbial metabolite production [511]. Short-chain fatty acids (SCFAs), including acetate, propionate, and butyrate, are important microbial metabolites that help regulate intestinal barrier integrity, neuroimmune responses, and inflammatory signaling pathways relevant to neurodegeneration [354]. Emerging evidence suggests that specific SD patterns may also be associated with distinct microbiota profiles and metabolic alterations. For example, SD patterns involving phlegm accumulation, qi stagnation, and spleen deficiency have been linked to intestinal dysbiosis and altered inflammatory responses [512]. These findings support the possibility that microbiota-associated SD phenotypes may reflect different biological and pathophysiological states in patients with cognitive impairment.

Within this framework, herbal medicines may exert therapeutic effects partly through modulation of the gut microbiota and gut–brain signaling pathways. Several herbal compounds and polyherbal formulations have been shown to restore microbial balance, reduce systemic inflammation, and improve SCFA production [513]. Therefore, integrating SD classification with microbiome profiling may contribute to the development of personalized and microbiome-responsive herbal therapies for AD and MCI. Future studies combining microbiome analysis, metabolomics, and clinical SD evaluation may improve patient stratification and optimize individualized treatment strategies in neurodegenerative disorders.

### 6.5. Improvements in Formulation and Delivery

Recent advances in formulation science and targeted delivery systems have therefore been developed to improve the therapeutic efficacy of herbal bioactive compounds in cognitive disorders. Nanotechnology-based delivery systems, including nanoparticles and liposomes, have shown significant promise in enhancing BBB penetration and improving neuroprotective effects. Evidence from nanotechnology-based applications in other disease models, including lung cancer, has similarly demonstrated that nanoparticle-mediated systems improve targeted drug delivery efficiency and diagnostic precision, further supporting the broader translational potential of nanomedicine for neurological disorders [514]. Nano-formulated compounds such as curcumin and *Ginkgo biloba* extracts demonstrated enhanced cognitive benefits and neuroprotection, while liposomal formulations of *Centella asiatica* improved stability and brain-targeted delivery [515]. Recent advancements in bioactive natural products combined with nanoparticle-mediated delivery systems have further demonstrated improved compound stability, bioavailability, and therapeutic targeting [516]. Another important strategy involves increasing the bioavailability of herbal compounds using phytosomes and nanoemulsions. Phytosome-based *Bacopa monnieri* formulations showed improved brain uptake, whereas nanoemulsified ashwagandha oil enhanced the delivery of lipophilic bioactive compounds and improved cognitive outcomes [515]. However, despite encouraging preclinical findings, the majority of this delivery systems remain insufficiently validated in large-scale human clinical studies, and their long-term therapeutic efficacy and safety profiles are not yet fully established.

Sustained and controlled release systems have also improved the stability and therapeutic delivery of herbal compounds. Hydrogels containing curcumin enabled prolonged and targeted release of bioactive molecules, thereby enhancing neuroprotective and anti-inflammatory effects [517]. Recent developments in stimulus-responsive hydrogels using dynamic boronic acid ester bonds further improved curcumin bioavailability and controlled-release properties [518].

Nevertheless, several important challenges remain unresolved, including manufacturing consistency, regulatory approval, long-term biocompatibility, potential nanotoxicity, hepatic accumulation, and altered herb–drug interactions resulting from enhanced systemic exposure [519,520,521]. Therefore, although advanced delivery systems may improve herbal compound delivery and bioavailability, further rigorous clinical evaluation and standardized safety assessments are required before their widespread clinical application in cognitive and neurological disorders can be fully established [189,519,520,522,523].

### 6.6. Regulatory and Quality Control Challenges in SD-Based Herbal Neurotherapeutics

Despite the growing therapeutic potential of herbal medicines in neurodegenerative disorders, several regulatory and quality-control challenges continue to limit the translational applicability of SD-based herbal formulations [524]. One major concern is batch-to-batch variability, as the phytochemical composition of medicinal plants can be significantly influenced by geographical origin, cultivation conditions, harvesting time, processing procedures, and storage conditions [525]. These variations are particularly problematic in polyherbal and syndrome differentiation (SD)-guided formulations, where complex interactions among multiple bioactive constituents complicate standardization, reproducibility, and therapeutic consistency.

Accurate authentication of medicinal plants is also essential to prevent species misidentification, substitution, contamination, and adulteration. Advanced analytical approaches, including chromatographic fingerprinting, metabolomic profiling, and DNA barcoding, are increasingly used to ensure botanical authenticity and product quality [526]. Pharmacokinetic variability represents another major challenge, as the absorption, metabolism, and bioavailability of herbal compounds may differ among individuals depending on age, disease condition, gut microbiota composition, and genetic background [527]. In addition, inconsistencies in herbal composition, extraction protocols, dosage regimens, and patient stratification contribute to reproducibility limitations in SD-guided clinical trials, thereby reducing translational credibility and regulatory acceptance. Therefore, rigorous quality-control systems, standardized manufacturing procedures, and harmonized clinical evaluation strategies are essential to improve the safety, reproducibility, and therapeutic reliability of herbal neurotherapeutics [528]. Future integration of omics technologies and globally standardized regulatory frameworks may further enhance the clinical translation and evidence-based application of herbal medicines for neurodegenerative diseases.

### 6.7. Safety Considerations and Herb–Drug Interactions in AD Patients

Safety concerns and herb–drug interactions remain major clinical considerations in elderly patients with AD, particularly because of polypharmacy and long-term medication use [529]. Although herbal medicines demonstrate promising neuroprotective effects in neurodegenerative disorders, several widely used herbal compounds, including ginseng, curcumin, licorice, and *Ginkgo biloba*, can interact with prescription medications through pharmacodynamic and pharmacokinetic mechanisms [530]. *Foeniculum vulgare* has likewise been recognized for its diverse phytochemical constituents and documented safety and herb–drug interaction profile, further emphasizing the need for careful clinical evaluation of herbal medicines in elderly AD patients [284]. For example, *Ginkgo biloba* has been associated with an increased risk of bleeding when co-administered with anticoagulants or antiplatelet agents such as aspirin and warfarin [531]. *Ginseng* may influence blood glucose regulation and alter the therapeutic effects of psychotropic medications, whereas excessive licorice consumption has been linked to hypertension, hypokalemia, and corticosteroid-like adverse effects [532]. In addition, curcumin and several herbal phytochemicals can modulate cytochrome P450 (CYP450) enzymes, thereby affecting the metabolism, bioavailability, and toxicity of cholinesterase inhibitors, antidepressants, antipsychotics, and other centrally acting medications [418]. Long-term or high-dose use of certain herbal supplements may also increase the risk of hepatotoxicity, gastrointestinal complications, and metabolic disturbances in susceptible individuals. These concerns are particularly important in elderly patients with impaired metabolic function, multiple comorbidities, and complex medication regimens. Therefore, the safe integration of herbal medicines into neurodegenerative disease management requires rigorous pharmacovigilance, standardized dosing strategies, careful clinical monitoring, and evidence-based safety evaluation. Future clinical studies should prioritize long-term safety assessment, herb–drug interaction profiling, and individualized risk evaluation in elderly neurodegenerative populations. Integrating pharmacogenomics, therapeutic monitoring, and precision-based prescribing approaches may further improve the safety and clinical applicability of herbal neurotherapeutics. A structured evidence and safety grading of selected herbal neurotherapeutics is summarized in Table 6.

### 6.8. Integration of Multi-Omics and Biomarkers in Precision Medicine for Neurodegeneration Protection

Precision medicine has emerged as an important strategy in neurodegenerative diseases by emphasizing patient-specific therapeutic approaches rather than a uniform treatment model. In AD, PD, and related disorders, disease heterogeneity is increasingly recognized at molecular, cellular, and systemic levels [543]. Multi-omics approaches, particularly transcriptomics, enable the identification of patient-specific gene expression patterns associated with neuroinflammation, synaptic dysfunction, and neuronal degeneration [544]. In parallel, neuroimaging biomarkers derived from MRI and PET imaging provide valuable structural and functional information for early diagnosis and disease progression monitoring. Additionally, gut microbiome signatures have been linked to neurodegenerative pathology through modulation of immune responses, microbial metabolites, and gut–brain axis signaling [545,546,547].

Within this framework, SD and individualized herbal interventions align closely with the principles of personalized medicine. Herbal formulations may differentially regulate molecular pathways depending on the patient’s biological profile and disease characteristics. Recent advances in artificial intelligence (AI) and machine learning further support precision medicine approaches by integrating transcriptomic, neuroimaging, and microbiome-derived datasets for patient stratification and individualized therapeutic selection [548]. AI-assisted analyses may also help predict patient-specific responses to herbal bioactive compounds, thereby improving therapeutic efficacy while minimizing adverse effects. Collectively, these integrative strategies highlight the growing potential of precision medicine to support targeted and adaptive interventions for complex neurodegenerative diseases [549].

### 6.9. Challenges and Limitations

Despite the growing therapeutic interest in herbal medicines for neurological and cognitive disorders, several important challenges and limitations remain. One major concern is the lack of standardized formulations and dosing protocols, which may lead to inconsistent therapeutic outcomes. Variability in cultivation conditions, harvesting methods, extraction procedures, and phytochemical composition further complicates reproducibility and quality control across studies [550,551,552]. In addition, herbal medicine regulations differ considerably among countries, making it difficult to establish globally harmonized standards for safety, efficacy, and product quality.

The overall quality of current clinical evidence also remains variable. Many published studies involve relatively small sample sizes, short intervention durations, and limited multicenter validation [87,391]. Furthermore, several studies lack randomized, placebo-controlled, or double-blinded designs, thereby reducing the strength and reproducibility of the findings [553]. Many SD-guided herbal studies are also region-specific, particularly within East Asian healthcare systems, which may limit their international generalizability. In addition, the absence of internationally standardized SD criteria contributes to inconsistencies in patient classification, herbal prescriptions, and therapeutic outcomes [554].

Safety concerns also remain important, particularly in elderly patients with polypharmacy. Herbal medicines may interact with conventional medications and produce adverse effects, especially when used at high doses or for prolonged periods [555]. Moreover, the blood–brain barrier may limit the ability of certain herbal compounds to effectively reach the central nervous system, thereby reducing therapeutic efficacy in neurological disorders [556]. Publication bias may further influence the interpretation of the available evidence, as studies reporting positive outcomes are more likely to be published than negative or inconclusive findings. Therefore, additional large-scale, multicenter, randomized, placebo-controlled clinical trials with standardized formulations and harmonized SD criteria are required to improve scientific rigor, reproducibility, and translational credibility in herbal neurotherapeutics [557].

### 6.10. Safety Concerns and Herb-Induced Liver Injury

Despite increasing interest in herbal neurotherapeutics for cognitive and neurological disorders, important safety concerns remain inadequately addressed. Although herbal medicines are often perceived as natural and therefore inherently safe, accumulating clinical evidence indicates that herbal products may contribute to herb-induced liver injury (HILI), particularly in elderly and poly-medicated individuals [558]. HILI has emerged as a growing global clinical challenge due to increased consumption of herbal supplements, variable product quality, and difficulties associated with causality assessment [559]. The clinical presentation of HILI is highly heterogeneous and may range from asymptomatic elevation of liver enzymes to severe hepatocellular injury, cholestatic liver injury, mixed-pattern hepatotoxicity, and, in rare cases, acute liver failure [558]. Several contributing factors have been associated with HILI development, including prolonged herbal consumption, excessive dosages, contamination with heavy metals or pesticides, product adulteration, inconsistent manufacturing processes, and variability in herbal composition. Multi-ingredient herbal formulations may further complicate identification of hepatotoxic components and increase the risk of unpredictable adverse reactions.

Drug-herb interactions represent another major concern in patients with cognitive disorders, who are commonly treated with multiple medications including cholinesterase inhibitors, antidepressants, antipsychotics, dopaminergic agents, and cardiovascular therapies [560]. Certain herbal compounds may influence cytochrome P450 enzymes, alter hepatic metabolism, or potentiate pharmacological effects, potentially leading to increased toxicity or altered therapeutic responses [561]. Older adults with neurodegenerative disorders may be particularly vulnerable because of age-related physiological decline, frailty, polypharmacy, impaired hepatic function, and reduced ability to accurately report herbal supplement intake [562]. Recent reports of skullcap-associated hepatotoxicity and other herbal-related liver injury cases further highlight the need for cautious clinical evaluation and rigorous pharmacovigilance. In addition, the absence of standardized formulations and regulatory inconsistencies across countries may contribute to variability in safety profiles and therapeutic outcomes [560,563]. Although advanced delivery systems such as nanoparticles and phytosomes may improve the bioavailability of herbal bioactive compounds, enhanced systemic exposure may also increase hepatic burden and potential toxicity risk [523]. Therefore, careful causality assessment, routine liver biochemistry monitoring in high-risk populations, standardized quality control, and large-scale clinical safety evaluations are essential before herbal neurotherapeutics can be more broadly integrated into the clinical management of cognitive and neurological disorders.

### 6.11. Future Directions

To ensure consistent therapeutic outcomes, future research should focus on developing standardized doses and formulations. Strong quality control procedures must be put in place to ensure the dependability and security of herbal medications [564]. More thorough, large-scale clinical research must be carried out to confirm the effectiveness and safety of herbal remedies. Evidence will be stronger if randomized, double-blind, placebo-controlled trial designs are used [565]. The goal of future studies should be to identify and assess novel herbal substances that may have positive effects on neurological and cognitive health. Working together with traditional healers and ethnobotanists might yield important information on new medicinal substances [562]. The bioavailability and effectiveness of herbal medications might be improved by using cutting-edge technologies like nanotechnology and bioinformatics, particularly when it comes to blood–brain barrier bridging [566]. Encouraging multidisciplinary research can result in novel herbal medicine techniques. Comprehensive approaches to treating neurological and cognitive problems can be fostered by cooperation between chemists, neurologists, pharmacologists, and other experts [126]. Strengthening the regulatory framework for herbal remedies is essential to guaranteeing their efficacy and security. Harmonising a trustworthy worldwide market for herbal medications might be facilitated by harmonizing laws across nations [567].

## 7. Conclusions

Herbal medicines represent promising complementary strategies for cognitive enhancement and neuroprotection through antioxidant, anti-inflammatory, and neurotransmitter-modulating activities. Bioactive compounds from herbs such as *Bacopa monnieri*, *Withania somnifera*, *Ginkgo biloba*, *ginseng*, *Glycyrrhiza glabra*, and *Moringa oleifera* have shown potential cognitive and neuroprotective effects in preclinical studies and selected clinical investigations. Advances in delivery systems, including nanoparticles, phytosomes, nanoemulsions, and hydrogels, may further improve the bioavailability, stability, and brain-targeting efficiency of herbal compounds.

Neurodegenerative disorders such as Alzheimer’s disease are increasingly recognized as multifactorial conditions involving oxidative stress, neuroinflammation, mitochondrial dysfunction, synaptic impairment, blood–brain barrier disruption, metabolic imbalance, and gut–brain axis dysregulation. Emerging evidence suggests that herbal medicines may modulate several of these interconnected pathological pathways simultaneously, supporting their potential as multi-target therapeutic approaches. In addition, advances in syndrome differentiation, transcriptomics, neuroimaging biomarkers, microbiome profiling, and systems biology highlight the growing potential of personalized and precision-based herbal interventions in neurodegenerative disease management. Despite these promising findings, the current evidence remains heterogeneous and limited by insufficient standardization, small-scale and region-specific studies, short intervention durations, and limited long-term clinical validation. Therefore, future research should prioritize standardized formulations, rigorous multicenter clinical trials, biomarker-guided therapeutic strategies, and stronger regulatory frameworks to improve reproducibility, safety, and translational applicability. Collectively, integrating traditional herbal medicine with modern precision medicine approaches may provide new opportunities for evidence-based neuroprotective interventions in cognitive disorders.

## Figures and Tables

**Table 1:** FDA-approved drug for AD and PD.

Brand Name	Active Ingredient	Route of Administration	Indication	Approval Year	Mechanism of Action	Target	Ref.
Aduhelm	Aducanumab-avwa	Intravenous infusion	AD	2021	↓ Amyloid-beta plaques	Monoclonal antibody	[132,133]
Belsomra	Suvorexant	Oral	AD	2014	↓ Orexin A and Orexin B	Orexin receptors (OX1R and OX2R)	[134,135]
Aricep	Donepezil hydrochloride	Oral	AD	1996	↓ Acetylcholinesterase, ↑ acetylcholine	Acetylcholinesterase enzyme	[94,136]
Razadyne	Galantamine hydrobromide	Oral	AD	2001	↓ Acetylcholinesterase, ↑ acetylcholine	Acetylcholinesterase enzyme, nicotinic acetylcholine receptors	[137]
Leqembi	Lecanemab-irmb	Intravenous (IV) infusion	AD	2023	↓ Amyloid-beta protofibrils	Monoclonal antibody	[138,139]
Namenda	Memantine hydrochloride	Oral	AD	2003	↓ Glutamate activity, NMDA receptors	NMDA (*N*-methyl-D-aspartate) receptor	[140]
Exelon	Rivastigmine tartrate	Oral, Transdermal patch	AD	2000	↓ Acetylcholinesterase, butyrylcholinesterase	Acetylcholinesterase enzyme, Butyrylcholinesterase enzyme	[94,141]
Azilect	Rasagiline	Oral	PD	2006	↓ Monoamine Oxidase-B (MAO-B), ↑ Dopamine	Monoamine Oxidase-B (MAO-B) Enzyme	[142,143]
Apokyn	Apomorphine	Subcutaneous (injection)	PD	2004	↑ Dopamine activity	Dopamine D2 receptors	[144]
Comtan	Entacapone	Oral	PD	1999	↑ Dopamine availability	Catechol-O-methyltransferase (COMT) enzyme	[144,145]
Duopa	Levodopa and Carbidopa	Intestinal infusion	PD	2015	↑ levodopa, Dopamine	Dopaminergic neurons	[146]
Gocovri	Amantadine	Oral	PD	2017	↓ Excitatory neurotransmission	*N*-Methyl-D-Aspartate (NMDA) receptors	[147]
Inbrija	Levodopa	Inhalation	PD	2018	↑ Dopamine	Dopaminergic neurons	[148]
Kynmobi	Apomorphine	Sublingual	PD	2020	↑ Dopamine	Dopamine receptors	[149]
Mirapex ER	Pramipexole	Oral	PD	1997	↑ Dopamine	Dopamine D2 and D3 receptors	[150]
Nourianz	Istradefylline	Oral	PD	2019	↑ Dopaminergic signaling	Adenosine A2A receptors	[151]
Ongentys	Opicapone	Oral	PD	2020	↓ levodopa-metabolizing	COMT enzyme	[152]
Osmolex ER	Amantadine	Oral	PD	2018	↑ Glutamatergic neurotransmission	NMDA receptors	[153]
Rytary	Levodopa and Carbidopa	Oral	PD	2015	↓ Peripheral conversion of levodopa	Dopaminergic neurons	[154]
Sinemet	Levodopa and Carbidopa	Oral	PD	1975	↓ levodopa	Dopaminergic neurons	[155]
Xadago	Safinamide	Oral	PD	2017	↑ Glutamate release, dopaminergic activity	MAO-B and sodium channels	[137]
Zelapar	Selegiline	Oral	PD	2006	↑ Dopamine	MAO-B	[144]

Abbreviation: ↓, downregulation; ↑, upregulation.

**Table 2:** Comparative summary of disease-specific pathological mechanisms, neurotransmitter alterations, and representative herbal therapeutic targets in cognitive disorders.

Disorder	Major Pathological Mechanisms	Neurotransmitter Alterations	Representative Herbal Therapeutic Targets/Compounds	Ref.
AD	Amyloid-β accumulation Tau hyperphosphorylation Oxidative stress Neuroinflammation Synaptic loss	↓ ACh, Glutamate dysregulation	Ginkgo biloba, Bacopa monnieri, curcumin, resveratrol	[225,226,227]
PD	Dopaminergic neuronal loss α-synuclein aggregation Mitochondrial dysfunction Oxidative stress	↓ Dopamine	Curcumin, Panax ginseng, green tea polyphenols, resveratrol	[228,229,230]
Schizophrenia	NMDA receptor hypofunction Oxidative stress Neuroinflammation Synaptic dysfunction	Dopamine dysregulation, glutamate imbalance, GABA alterations	Flavonoids, alkaloids, antioxidant phytochemicals	[231,232,233]
Depression	HPA-axis dysregulation Neuroinflammation Impaired neuroplasticity Oxidative stress	↓ GABA, serotonin alterations, glutamate dysregulation	Crocus sativus, Withania somnifera, curcumin	[234,235,236]
Vascular dementia	Cerebral hypoperfusion Ischemic injury Oxidative stress Endothelial dysfunction Neuroinflammation	Glutamate excitotoxicity, cholinergic dysfunction	Antioxidant-rich herbs, ginseng, and polyphenols	[237,238,239]
Mild cognitive impairment	Early synaptic dysfunction Metabolic stress Neuroinflammation	Cholinergic decline, neurotransmitter imbalance	Bacopa monnieri, Ginkgo biloba, omega-rich botanicals	[240,241,242,243]

Abbreviation: ↓, downregulation.

**Table 4:** Modulatory effects of herbal compounds on gut microbiota and associated neuroprotective mechanisms through the gut–brain axis.

Herbal Compound	Gut Microbiota Alterations	Gut-Level Mechanism	Neuroprotective Effect	Ref.
Polyphenol-rich herbs	↑ *Lactobacillus*, ↑ *Bifidobacterium*	Prebiotic-like activity and microbial fermentation	Reduces neuroinflammation Improves cognition	[357]
Ginseng	↑ *Akkermansia muciniphila*, ↑ SCFA producers	Enhances mucosal integrity and microbial diversity	Improves BBB function and antidepressant effects	[358]
Curcumin	↑ *Lactobacillus*, ↓ Pathogenic bacteria	Modulates gut inflammation and oxidative stress	Reduce microglial activation and anti-amyloid effects	[359]
Resveratrol	↑ Bifidobacterium, ↑ Butyrate-producing bacteria	Enhances SCFA production and gut barrier integrity	Neuroprotection through anti-inflammatory signaling	[360]
Berberine	↑ *Akkermansia*, ↓ *Escherichia*–*Shigella*	Improves gut permeability and metabolic balance	Reduces neuroinflammation via decreases LPS signaling	[361]
Traditional Chinese herbal mixtures	↑ *Lactobacillus*, ↑ *Bifidobacterium*	Restores microbial balance and SCFA metabolism	Modulates serotonin signaling and mood regulation	[362]
Flavonoid rich herbs	↑ SCFA-producing bacteria	Increases butyrate and propionate production	Improves BBB integrity and neuronal survival	[363]
Ginger	↑ Beneficial *Firmicutes*	Anti-inflammatory modulation of gut microbiota	Reduces oxidative stress in the CNS	[364]
Garlic	↑ Lactobacillus, ↓ pathogenic bacteria	Antimicrobial and prebiotic effects	Neuroimmune regulation through the gut–brain axis	[365]
Herbal polysaccharides	↑ *Bifidobacterium*, ↑ *Akkermansia*	Enhances SCFA production	Vagus nerve activation and neuroprotection	[366]

Abbreviation: ↓, downregulation; ↑, upregulation.

**Table 5:** Cognitive and metabolic effects of selected traditional medicinal extracts.

Compound/Extract	Dose/Duration	Efficacy	Mechanism	Reference
Curcumin	500 mg/day for 3 months	↑ cognitive functions in older adults	↓ NF-κB pathway, ↓ neuroinflammation	[297,475]
Resveratrol	150 mg/day for 1 year	↑ memory function	↑ mitochondrial function by activating SIRT1	[476,477]
Berberine	500 mg × 3/day for 12 weeks	↓ blood lipid and glucose level, ↑ cognitive function	Activates AMPK, ↓ neuroinflammation	[478]
Quercetin	500 mg/day for 3 months	↓ blood pressure, ↑ memory function	Inhibits lipid peroxidation, antioxidant activity	[479]
*Epigallocatechin gallate*	300–800 mg/day for 3 months	↑ cholesterol metabolism, ↓ mental fatigue	↑ LDL receptor expression, ↑ neurogenesis, ↓ oxidative stress	[480]
*Polygala tenuifolia*	100–300 mg/day for 3 months	↑ memory function and attention	Enhances neuroplasticity, ↓ oxidative stress	[481]
*Cistanche tubulosa*	300–600 mg/day for 84 days	↑ learning and memory performance	↑ acetylcholine levels	[482]
*Ginkgo biloba*	120–140 mg/day for 24 weeks	Slowing cognitive decline in MCI and aging	↑ cerebral blood flow	[483]
*Bacopa monnieri*	300 mg/day for 3 months	↑ memory retention	↓ β-amyloid accumulation	[483]

Abbreviation: ↓, downregulation; ↑, upregulation.

**Table 6:** Summary of clinical evidence, safety profile, and formulation status of selected herbal neurotherapeutics in cognitive and neurological disorders.

Herb	Evidence Level	Meta-Analytical Evidence	Adverse Effects/Interactions	Hepatotoxicity/Safety Signal	Standardized Formulation	Ref.
*Bacopa monnieri*	Preclinical + clinical	Limited	Mild GI effects, minimal drug–herb interaction risk	Rare	Partially standardized extracts available	[533]
*Ginkgo biloba*	Preclinical + clinical	Yes	Headache, dizziness, bleeding risk	Rare	Standardized EGb 761 available	[534]
*Withania somnifera*	Preclinical + clinical	Limited	Drowsiness, GI upset	Reported HILI cases	Partially standardized	[535,536]
*Panax ginseng*	Preclinical + clinical	Limited	Insomnia, hypertension	Rare	standardized extracts available	[537,538]
*Centella asiatica*	Preclinical	Limited	Allergic reactions	Limited reports	Not fully standardized	[539]
Curcumin	Preclinical + clinical	Multiple meta-analysis	GI discomfort	Rare	Advanced formulations available	[540]
*Glycyrrhiza glabra*	Preclinical + clinical	Limited	Hypertension, hypokalemia	Possible hepatic interaction	Variable	[541]
*Moringa oleifera*	Mainly preclinical	Limited	GI discomfort	Limited reports	Not standardized	[542]

## Data Availability

Not applicable.

## References

[1] Hebert L.E., Weuve J., Scherr P.A., Evans D.A. (2013). Alzheimer disease in the United States (2010–2050) estimated using the 2010 census. Neurology.

[2] Qian W., Schweizer T., Munoz D., Fischer C.E. (2016). O3-04-06: Misdiagnosis of Alzheimer’s Disease: Inconsistencies Between Clinical Diagnosis and Neuropathological Confirmation. Alzheimer’s Dement..

[3] Pais R., Ruano L., P. Carvalho O., Barros H. (2020). Global cognitive impairment prevalence and incidence in community dwelling older adults—A systematic review. Geriatrics.

[4] Palmer N.P., Ortega B.T., Joshi P. (2022). Cognitive impairment in older adults: Epidemiology, diagnosis, and treatment. Psychiatr. Clin..

[5] Heo H.-M., Lee K.-H., Heo K.-H., Hwang Y.-C., Lee H.-G., Kwon S., Cho S.-Y., Park S.-U., Jung W.-S., Moon S.-K. (2025). Analysis of the relationship between brain activation and clinical indicators in amnestic mild cognitive impairment. Brain Imaging Behav..

[6] Luchsinger J.A. (2012). Type 2 diabetes and cognitive impairment: Linking mechanisms. J. Alzheimer’s Dis..

[7] Park M., Kwon D., Jung J., Han C., Jo I., Jo S. (2013). Mini-Mental Status Examination as predictors of mortality in the elderly. Acta Psychiatr. Scand..

[8] Roberts R., Knopman D.S. (2013). Classification and epidemiology of MCI. Clin. Geriatr. Med..

[9] Park K., Lee W.H., Cho E., Kong C.H., Min H.S., Kim M.S., Han J.E., Jung S.Y., Kim D.H., Ryu J.H. (2025). The effects of Cheonwangbosim-dan, a traditional herbal medicine prescription, on scopolamine-induced cognitive dysfunction in mice. J. Ethnopharmacol..

[10] Liu L.-Y., Lu Y., Shen L., Li C.-B., Yu J.-T., Yuan C.R., Ye K.X., Chao Y.X., Shen Q.-F., Mahendran R. (2022). Prevalence, risk and protective factors for mild cognitive impairment in a population-based study of Singaporean elderly. J. Psychiatr. Res..

[11] Xu T., Bu G., Yuan L., Zhou L., Yang Q., Zhu Y., Zhang S., Liu Q., Ouyang Z., Yang X. (2024). The prevalence and risk factors study of cognitive impairment: Analysis of the elderly population of Han nationality in Hunan province, China. CNS Neurosci. Ther..

[12] Pessoa R.M.P., Bomfim A.J.L., Ferreira B.L.C., Chagas M.H.N. (2019). Diagnostic criteria and prevalence of mild cognitive impairment in older adults living in the community: A systematic review and meta-analysis. Arch. Clin. Psychiatry.

[13] Sharma C., Kim S., Nam Y., Jung U.J., Kim S.R. (2021). Mitochondrial dysfunction as a driver of cognitive impairment in Alzheimer’s disease. Int. J. Mol. Sci..

[14] Jack C.R., Knopman D.S., Chételat G., Dickson D., Fagan A.M., Frisoni G.B., Jagust W., Mormino E.C., Petersen R.C., Sperling R.A. (2016). Suspected non-Alzheimer disease pathophysiology—Concept and controversy. Nat. Rev. Neurol..

[15] Dourlen P., Kilinc D., Malmanche N., Chapuis J., Lambert J.-C. (2019). The new genetic landscape of Alzheimer’s disease: From amyloid cascade to genetically driven synaptic failure hypothesis?. Acta Neuropathol..

[16] Kim J., Chakrabarty P., Hanna A., March A., Dickson D.W., Borchelt D.R., Golde T., Janus C. (2013). Normal cognition in transgenic BRI2-Aβ mice. Mol. Neurodegener..

[17] Kametani F., Hasegawa M. (2018). Reconsideration of amyloid hypothesis and tau hypothesis in Alzheimer’s disease. Front. Neurosci..

[18] Kozlov S., Afonin A., Evsyukov I., Bondarenko A. (2017). Alzheimer’s disease: As it was in the beginning. Rev. Neurosci..

[19] Chakravorty A., Jetto C.T., Manjithaya R. (2019). Dysfunctional mitochondria and mitophagy as drivers of Alzheimer’s disease pathogenesis. Front. Aging Neurosci..

[20] Albensi B.C. (2019). Dysfunction of mitochondria: Implications for Alzheimer’s disease. Int. Rev. Neurobiol..

[21] Marois A., Lafond D. (2022). Augmenting cognitive work: A review of cognitive enhancement methods and applications for operational domains. Cogn. Technol. Work.

[22] Malík M., Tlustoš P. (2022). Nootropics as cognitive enhancers: Types, dosage and side effects of smart drugs. Nutrients.

[23] Dresler M., Sandberg A., Ohla K., Bublitz C., Trenado C., Mroczko-Wąsowicz A., Kühn S., Repantis D. (2013). Non-pharmacological cognitive enhancement. Neuropharmacology.

[24] Chen M., Ruan G., Chen L., Ying S., Li G., Xu F., Xiao Z., Tan Y., Lv L., Ping Y. (2022). Neurotransmitter and intestinal interactions: Focus on the microbiota-gut-brain axis in irritable bowel syndrome. Front. Endocrinol..

[25] Hamamah S., Aghazarian A., Nazaryan A., Hajnal A., Covasa M. (2022). Role of microbiota-gut-brain axis in regulating dopaminergic signaling. Biomedicines.

[26] Dicks L.M. (2022). Gut bacteria and neurotransmitters. Microorganisms.

[27] Di Vincenzo F., Del Gaudio A., Petito V., Lopetuso L.R., Scaldaferri F. (2024). Gut microbiota, intestinal permeability, and systemic inflammation: A narrative review. Intern. Emerg. Med..

[28] Mou Y., Du Y., Zhou L., Yue J., Hu X., Liu Y., Chen S., Lin X., Zhang G., Xiao H. (2022). Gut microbiota interact with the brain through systemic chronic inflammation: Implications on neuroinflammation, neurodegeneration, and aging. Front. Immunol..

[29] Dong Y., Cui C. (2022). The role of short-chain fatty acids in central nervous system diseases. Mol. Cell. Biochem..

[30] Li C., Yao J., Yang C., Yu S., Yang Z., Wang L., Li S., He N. (2025). Gut microbiota-derived short chain fatty acids act as mediators of the gut-liver-brain axis. Metab. Brain Dis..

[31] Alpino G.d.C.Á., Pereira-Sol G.A., Dias M.d.M.e., Aguiar A.S.d., Peluzio M.d.C.G. (2024). Beneficial effects of butyrate on brain functions: A view of epigenetic. Crit. Rev. Food Sci. Nutr..

[32] Rahman Z., Dandekar M.P. (2023). Implication of paraprobiotics in age-associated gut dysbiosis and neurodegenerative diseases. NeuroMol. Med..

[33] Grant H., Anderton R., Gasson N., Lawrence B.J. (2023). The gut microbiome and cognition in Parkinson’s disease: A systematic review. Nutr. Neurosci..

[34] Kim N.Y., Gowda S.G.S., Lee S.-G., Sethi G., Ahn K.S. (2024). Cannabidiol induces ERK activation and ROS production to promote autophagy and ferroptosis in glioblastoma cells. Chem.-Biol. Interact..

[35] Yadav D., Kumar P. (2022). Restoration and targeting of aberrant neurotransmitters in Parkinson’s disease therapeutics. Neurochem. Int..

[36] Banerjee S., McCracken S., Hossain M.F., Slaughter G. (2020). Electrochemical detection of neurotransmitters. Biosensors.

[37] Jameson K.G., Olson C.A., Kazmi S.A., Hsiao E.Y. (2020). Toward understanding microbiome-neuronal signaling. Mol. Cell.

[38] Harilal S., Kumar R., Mathew G.E., Jose J., Uddin M.S., Mathew B. (2020). Neurochemicals in nervous system and exploring the chemical make-up of human brain. Principles of Neurochemistry.

[39] Xia X., Wang Y., Qin Y., Zhao S., Zheng J.C. (2022). Exosome: A novel neurotransmission modulator or non-canonical neurotransmitter?. Ageing Res. Rev..

[40] Bhat S., El-Kasaby A., Freissmuth M., Sucic S. (2021). Functional and biochemical consequences of disease variants in neurotransmitter transporters: A special emphasis on folding and trafficking deficits. Pharmacol. Ther..

[41] García-Cazorla À., Artuch R. (2020). Neurotransmitter disorders. Rosenberg’s Molecular and Genetic Basis of Neurological and Psychiatric Disease.

[42] Mastrangelo M. (2021). Epilepsy in inherited neurotransmitter disorders: Spotlights on pathophysiology and clinical management. Metab. Brain Dis..

[43] Pichai E., Lakshmanan M. (2021). Neurotransmitters and Neurotransmission. Introduction to Basics of Pharmacology and Toxicology.

[44] Talebi M., Talebi M., Samarghandian S. (2021). Association of Crocus sativus with cognitive dysfunctions and Alzheimer’s disease: A systematic review. Biointerface Res. Appl. Chem..

[45] Dinparast L., Zengin G., Bahadori M.B. (2021). Cholinesterases inhibitory activity of 1H-benzimidazole derivatives. Biointerface Res. Appl. Chem..

[46] Zhao X., Li C., Ding G., Heng Y., Li A., Wang W., Hou H., Wen J., Zhang Y. (2021). The burden of Alzheimer’s disease mortality in the United States, 1999–2018. J. Alzheimer’s Dis..

[47] Liang C.-S., Li D.-J., Yang F.-C., Tseng P.-T., Carvalho A.F., Stubbs B., Thompson T., Mueller C., Shin J.I., Radua J. (2021). Mortality rates in Alzheimer’s disease and non-Alzheimer’s dementias: A systematic review and meta-analysis. Lancet Healthy Longev..

[48] Lin S.-Y., Lin P.-C., Lin Y.-C., Lee Y.-J., Wang C.-Y., Peng S.-W., Wang P.-N. (2022). The clinical course of early and late mild cognitive impairment. Front. Neurol..

[49] Hou Y., Dan X., Babbar M., Wei Y., Hasselbalch S.G., Croteau D.L., Bohr V.A. (2019). Ageing as a risk factor for neurodegenerative disease. Nat. Rev. Neurol..

[50] Uddin M.S., Hasana S., Hossain M.F., Islam M.S., Behl T., Perveen A., Hafeez A., Ashraf G.M. (2021). Molecular genetics of early-and late-onset Alzheimer’s disease. Curr. Gene Ther..

[51] Zhang X., Tian Y., Wang Z., Ma Y., Tan L., Yu J. (2021). The epidemiology of Alzheimer’s disease modifiable risk factors and prevention. J. Prev. Alzheimers Dis..

[52] Adani G., Filippini T., Garuti C., Malavolti M., Vinceti G., Zamboni G., Tondelli M., Galli C., Costa M., Vinceti M. (2020). Environmental risk factors for early-onset Alzheimer’s dementia and frontotemporal dementia: A case-control study in northern Italy. Int. J. Environ. Res. Public Health.

[53] de Lange A.M.G., Barth C., Kaufmann T., Maximov I.I., van der Meer D., Agartz I., Westlye L.T. (2020). Women’s brain aging: Effects of sex-hormone exposure, pregnancies, and genetic risk for Alzheimer’s disease. Hum. Brain Mapp..

[54] Kapoor M., Chinnathambi S. (2023). TGF-β1 signalling in Alzheimer’s pathology and cytoskeletal reorganization: A specialized Tau perspective. J. Neuroinflamm..

[55] Urban A.S., Pavlov K.V., Kamynina A.V., Okhrimenko I.S., Arseniev A.S., Bocharov E.V. (2021). Structural studies providing insights into production and conformational behavior of amyloid-β peptide associated with Alzheimer’s disease development. Molecules.

[56] Corsi A., Bombieri C., Valenti M.T., Romanelli M.G. (2022). Tau isoforms: Gaining insight into MAPT alternative splicing. Int. J. Mol. Sci..

[57] Costa H.N., Esteves A.R., Empadinhas N., Cardoso S.M. (2023). Parkinson’s disease: A multisystem disorder. Neurosci. Bull..

[58] Fang C., Lv L., Mao S., Dong H., Liu B. (2020). Cognition deficits in Parkinson’s disease: Mechanisms and treatment. Park. Dis..

[59] Bhatia V., Sharma S. (2021). Role of mitochondrial dysfunction, oxidative stress and autophagy in progression of Alzheimer’s disease. J. Neurol. Sci..

[60] Perluigi M., Di Domenico F., Butterfield D.A. (2024). Oxidative damage in neurodegeneration: Roles in the pathogenesis and progression of Alzheimer disease. Physiol. Rev..

[61] Bai R., Guo J., Ye X.-Y., Xie Y., Xie T. (2022). Oxidative stress: The core pathogenesis and mechanism of Alzheimer’s disease. Ageing Res. Rev..

[62] Misrani A., Tabassum S., Yang L. (2021). Mitochondrial dysfunction and oxidative stress in Alzheimer’s disease. Front. Aging Neurosci..

[63] Pfundstein G., Nikonenko A.G., Sytnyk V. (2022). Amyloid precursor protein (APP) and amyloid β (Aβ) interact with cell adhesion molecules: Implications in Alzheimer’s disease and normal physiology. Front. Cell Dev. Biol..

[64] Zhang Y., Wang M., Chang W. (2022). Iron dyshomeostasis and ferroptosis in Alzheimer’s disease: Molecular mechanisms of cell death and novel therapeutic drugs and targets for AD. Front. Pharmacol..

[65] Ma H., Dong Y., Chu Y., Guo Y., Li L. (2022). The mechanisms of ferroptosis and its role in alzheimer’s disease. Front. Mol. Biosci..

[66] Reggiori F., Molinari M. (2022). ER-phagy: Mechanisms, regulation, and diseases connected to the lysosomal clearance of the endoplasmic reticulum. Physiol. Rev..

[67] Sahoo S., Padhy A.A., Kumari V., Mishra P. (2022). Role of ubiquitin–proteasome and autophagy-lysosome pathways in α-synuclein aggregate clearance. Mol. Neurobiol..

[68] Patabendige A., Janigro D. (2023). The role of the blood–brain barrier during neurological disease and infection. Biochem. Soc. Trans..

[69] Andjelkovic A.V., Situ M., Citalan-Madrid A.F., Stamatovic S.M., Xiang J., Keep R.F. (2023). Blood-brain barrier dysfunction in normal aging and neurodegeneration: Mechanisms, impact, and treatments. Stroke.

[70] Ahlawat A., Walia V., Garg M. (2025). Brain insulin resistance mediated cognitive impairment and neurodegeneration: Type-3 diabetes or Alzheimer’s Disease. Acta Neurol. Belg..

[71] González A., Calfío C., Churruca M., Maccioni R.B. (2022). Glucose metabolism and AD: Evidence for a potential diabetes type 3. Alzheimer’s Res. Ther..

[72] Alzarea S.I. (2025). Non-coding RNA-mediated gene regulation in Alzheimer’s disease pathogenesis: Molecular insights and emerging innovations. Saudi Pharm. J..

[73] Lossi L., Castagna C., Merighi A. (2024). An overview of the epigenetic modifications in the brain under normal and pathological conditions. Int. J. Mol. Sci..

[74] Mehder R.H. (2020). Neuronal Oxidative Stress and Dendritic Trimming in a Mouse Model of Late Onset Alzheimer’s Disease. Ph.D. Thesis.

[75] Huang Q., Liao C., Ge F., Ao J., Liu T. (2022). Acetylcholine bidirectionally regulates learning and memory. J. Neurorestoratology.

[76] Bekdash R.A. (2021). The cholinergic system, the adrenergic system and the neuropathology of Alzheimer’s disease. Int. J. Mol. Sci..

[77] Gunday E., Deniz F.S.S. (2026). Alzheimer’s Disease and Contemporary Therapeutic Approaches: Recent Advances in Natural Products. Drugs Drug Candidates.

[78] Madav Y., Wairkar S., Prabhakar B. (2019). Recent therapeutic strategies targeting beta amyloid and tauopathies in Alzheimer’s disease. Brain Res. Bull..

[79] Roy P. (2023). Cerebrovascular Disease Related to Hypertension: Effects of Antioxidant and Cholinergic Precursor Molecules. Ph.D. Thesis.

[80] Wang W., Zhao F., Ma X., Perry G., Zhu X. (2020). Mitochondria dysfunction in the pathogenesis of Alzheimer’s disease: Recent advances. Mol. Neurodegener..

[81] Giorgi F.S., Galgani A., Gaglione A., Ferese R., Fornai F. (2020). Effects of prolonged seizures on basal forebrain cholinergic neurons: Evidence and potential clinical relevance. Neurotox. Res..

[82] Govind N. (2020). Donepezil for dementia due to Alzheimer’s disease. Br. J. Community Nurs..

[83] Grutzendler J., Morris J.C. (2001). Cholinesterase inhibitors for Alzheimer’s disease. Drugs.

[84] Moreira N.C.d.S., Lima J.E.B.d.F., Marchiori M.F., Carvalho I., Sakamoto-Hojo E.T. (2022). Neuroprotective effects of cholinesterase inhibitors: Current scenario in therapies for Alzheimer’s disease and future perspectives. J. Alzheimer’s Dis. Rep..

[85] Yiannopoulou K.G., Papageorgiou S.G. (2013). Current and future treatments for Alzheimer’s disease. Ther. Adv. Neurol. Disord..

[86] Bayo Jimenez M.T., Rivas-García L., Sánchez-González C., Grosso G., Lipari V., Vera-Ramírez L., Battino M., Giampieri F., Quiles J.L., Forbes-Hernández T.Y. (2025). Natural Products in Alzheimer’s Disease: A Systematic Review of Clinical Trials and Underlying Molecular Mechanisms. Int. J. Mol. Sci..

[87] Nahar L., Charoensup R., Kalieva K., Habibi E., Guo M., Wang D., Kvasnica M., Onder A., Sarker S. (2025). Natural products in neurodegenerative diseases: Recent advances and future outlook. Front. Pharmacol..

[88] Alanko V., Udeh-Momoh C., Kivipelto M., Sandebring-Matton A. (2022). Mechanisms underlying non-pharmacological dementia prevention strategies: A translational perspective. J. Prev. Alzheimer’s Dis..

[89] Direito R., Barbalho S.M., Sepodes B., Figueira M.E. (2024). Plant-derived bioactive compounds: Exploring neuroprotective, metabolic, and hepatoprotective effects for health promotion and disease prevention. Pharmaceutics.

[90] Vecchio I., Sorrentino L., Paoletti A., Marra R., Arbitrio M. (2021). The state of the art on acetylcholinesterase inhibitors in the treatment of Alzheimer’s disease. J. Cent. Nerv. Syst. Dis..

[91] Cheng Y.-J., Lin C.-H., Lane H.-Y. (2021). Involvement of cholinergic, adrenergic, and glutamatergic network modulation with cognitive dysfunction in Alzheimer’s disease. Int. J. Mol. Sci..

[92] Yang Z., Zou Y., Wang L. (2023). Neurotransmitters in prevention and treatment of Alzheimer’s disease. Int. J. Mol. Sci..

[93] Begines P., Fernández-Bolaños J.G., López Ó. (2026). An updated patent review of acetylcholinesterase inhibitors for the treatment of alzheimer’s disease (2021–present). Expert Opin. Ther. Pat..

[94] Marucci G., Buccioni M., Dal Ben D., Lambertucci C., Volpini R., Amenta F. (2021). Efficacy of acetylcholinesterase inhibitors in Alzheimer’s disease. Neuropharmacology.

[95] Haake A., Nguyen K., Friedman L., Chakkamparambil B., Grossberg G.T. (2020). An update on the utility and safety of cholinesterase inhibitors for the treatment of Alzheimer’s disease. Expert Opin. Drug Saf..

[96] Akıncıoğlu H., Gülçin İ. (2020). Potent acetylcholinesterase inhibitors: Potential drugs for Alzheimer’s disease. Mini Rev. Med. Chem..

[97] d’Angremont E., Begemann M.J., Van Laar T., Sommer I.E. (2023). Cholinesterase inhibitors for treatment of psychotic symptoms in Alzheimer disease and Parkinson disease: A meta-analysis. JAMA Neurol..

[98] Wang H.J., Chinna-Meyyappan A., Feldman O.J., Lanctôt K.L. (2024). Emerging therapies for treatment of agitation, psychosis, or apathy in Alzheimer’s disease. Expert Opin. Emerg. Drugs.

[99] Kim M.G., Woo S.-H., Kim G.-W., Choi H.-K., Kim K.K., Koo B.S. (2026). Efficacy and safety of Woohwangchungsimwon in combination with donepezil for behavioral and psychological symptoms of dementia in probable Alzheimer’s disease: An assessor-blinded randomized controlled trial. J. Alzheimer’s Dis..

[100] Garcia Ribas G., Ferrer-Picón E. (2026). Tolerability of rivastigmine transdermal patch in patients with Alzheimer’s disease: A narrative review. Expert Opin. Drug Saf..

[101] Frangež R., Rouleau J., Molgó J., Žužek M.C., Benoit E., Guillou C. (2026). Novel short-acting non-depolarizing muscle relaxants derived from galantamine: Design, synthesis, and pharmacological evaluation. Biomed. Pharmacother..

[102] Shir D., Lachner C. (2026). Alzheimer’s disease: A clinical update on diagnosis and treatment. Neurol. I Neurochir. Pol..

[103] Hussein A., Guevara C.A., Del Valle P., Gupta S., Benson D.L., Huntley G.W. (2023). Non-motor symptoms of Parkinson’s disease: The neurobiology of early psychiatric and cognitive dysfunction. Neuroscientist.

[104] Eichel H.v., Heine J., Wegner F., Rogozinski S., Stiel S., Groh A., Krey L., Höglinger G.U., Klietz M. (2022). Neuropsychiatric symptoms in Parkinson’s disease patients are associated with reduced health-related quality of life and increased caregiver burden. Brain Sci..

[105] Bae Y.J., Kim J.-M., Sohn C.-H., Choi J.-H., Choi B.S., Song Y.S., Nam Y., Cho S.J., Jeon B., Kim J.H. (2021). Imaging the substantia nigra in Parkinson disease and other Parkinsonian syndromes. Radiology.

[106] Afitska K., Fucikova A., Shvadchak V.V., Yushchenko D.A. (2019). α-Synuclein aggregation at low concentrations. Biochim. Biophys. Acta (BBA)-Proteins Proteom..

[107] Pan L., Meng L., He M., Zhang Z. (2021). Tau in the pathophysiology of Parkinson’s disease. J. Mol. Neurosci..

[108] Negi S., Khurana N., Duggal N. (2024). The Misfolding Mystery: α-syn and the Pathogenesis of Parkinson’s Disease. Neurochem. Int..

[109] Nguyen M., Wong Y.C., Ysselstein D., Severino A., Krainc D. (2019). Synaptic, mitochondrial, and lysosomal dysfunction in Parkinson’s disease. Trends Neurosci..

[110] Grassi D., Howard S., Zhou M., Diaz-Perez N., Urban N.T., Guerrero-Given D., Kamasawa N., Volpicelli-Daley L.A., LoGrasso P., Lasmézas C.I. (2018). Identification of a highly neurotoxic α-synuclein species inducing mitochondrial damage and mitophagy in Parkinson’s disease. Proc. Natl. Acad. Sci. USA.

[111] Burbulla L.F., Song P., Mazzulli J.R., Zampese E., Wong Y.C., Jeon S., Santos D.P., Blanz J., Obermaier C.D., Strojny C. (2017). Dopamine oxidation mediates mitochondrial and lysosomal dysfunction in Parkinson’s disease. Science.

[112] Himmelberg M.M., West R.J., Elliott C.J., Wade A.R. (2018). Abnormal visual gain control and excitotoxicity in early-onset Parkinson’s disease Drosophila models. J. Neurophysiol..

[113] Anand David A., Arulmoli R., Parasuraman S. (2016). Overviews of biological importance of quercetin: A bioactive flavonoid. Pharmacogn. Rev..

[114] Mekhalfi M., Berteina-Raboin S. (2026). Mucuna pruriens: A Dietary Supplement with Balancing Properties That Can Limit Neurological Disorders and Associated Depressive States. Sci. Pharm..

[115] Yang G., Wang Y., Sun J., Zhang K., Liu J. (2016). *Ginkgo biloba* for mild cognitive impairment and Alzheimer’s disease: A systematic review and meta-analysis of randomized controlled trials. Curr. Top. Med. Chem..

[116] Balestrino R., Schapira A.H. (2020). Parkinson disease. Eur. J. Neurol..

[117] Kolodkin A.N., Sharma R.P., Colangelo A.M., Ignatenko A., Martorana F., Jennen D., Briedé J.J., Brady N., Barberis M., Mondeel T.D. (2020). ROS networks: Designs, aging, Parkinson’s disease and precision therapies. npj Syst. Biol. Appl..

[118] Pervin M., Unno K., Ohishi T., Tanabe H., Miyoshi N., Nakamura Y. (2018). Beneficial effects of green tea catechins on neurodegenerative diseases. Molecules.

[119] Armstrong M.J., Okun M.S. (2020). Diagnosis and treatment of Parkinson disease: A review. JAMA.

[120] Stocchi F., Antonini A., Berg D., Bergmans B., Jost W., Katzenschlager R., Kulisevsky J., Odin P., Valldeoriola F., Ray Chaudhuri K. (2022). Safinamide in the treatment pathway of Parkinson’s disease: A European Delphi consensus. npj Park. Dis..

[121] Lang A.E., Espay A.J. (2018). Disease modification in Parkinson’s disease: Current approaches, challenges, and future considerations. Mov. Disord..

[122] Vijiaratnam N., Simuni T., Bandmann O., Morris H.R., Foltynie T. (2021). Progress towards therapies for disease modification in Parkinson’s disease. Lancet Neurol..

[123] Radder D.L., Lígia Silva de Lima A., Domingos J., Keus S.H., van Nimwegen M., Bloem B.R., de Vries N.M. (2020). Physiotherapy in Parkinson’s disease: A meta-analysis of present treatment modalities. Neurorehabilit. Neural Repair.

[124] Nuzzo D. (2021). Role of natural antioxidants on neuroprotection and neuroinflammation. Antioxidants.

[125] Cilia R., Laguna J., Cassani E., Cereda E., Pozzi N.G., Isaias I.U., Contin M., Barichella M., Pezzoli G. (2017). Mucuna pruriens in Parkinson disease: A double-blind, randomized, controlled, crossover study. Neurology.

[126] Goyal R., Mittal P., Gautam R.K., Kamal M.A., Perveen A., Garg V., Alexiou A., Saboor M., Haque S., Farhana A. (2024). Natural products in the management of neurodegenerative diseases. Nutr. Metab..

[127] Cheong S.L., Federico S., Spalluto G., Klotz K.-N., Pastorin G. (2019). The current status of pharmacotherapy for the treatment of Parkinson’s disease: Transition from single-target to multitarget therapy. Drug Discov. Today.

[128] Kulisevsky J., Oliveira L., Fox S.H. (2018). Update in therapeutic strategies for Parkinson’s disease. Curr. Opin. Neurol..

[129] Mantovani E., Zucchella C., Argyriou A.A., Tamburin S. (2023). Treatment for cognitive and neuropsychiatric non-motor symptoms in Parkinson’s disease: Current evidence and future perspectives. Expert Rev. Neurother..

[130] Gonzalez-Latapi P., Bhowmick S.S., Saranza G., Fox S.H. (2020). Non-dopaminergic treatments for motor control in Parkinson’s disease: An update. CNS Drugs.

[131] Jing X.-Z., Yuan X.-Z., Luo X., Zhang S.-Y., Wang X.-P. (2023). An Update on Nondopaminergic Treatments for Motor and Non-motor symptoms of Parkinson’s disease. Curr. Neuropharmacol..

[132] Haddad H., Malone G.W., Comardelle N.J., Degueure A.E., Poliwoda S., Kaye R.J., Murnane K.S., Kaye A.M., Kaye A.D. (2022). Aduhelm, a novel anti-amyloid monoclonal antibody, for the treatment of Alzheimer’s disease: A comprehensive review. Health Psychol. Res..

[133] Dunn B., Stein P., Cavazzoni P. (2021). Approval of aducanumab for Alzheimer disease—The FDA’s perspective. JAMA Intern. Med..

[134] Uslaner J.M., Herring W.J., Coleman P.J. (2020). The discovery of suvorexant: Lessons learned that can be applied to other CNS drug development efforts. ACS Pharmacol. Transl. Sci..

[135] Varadharajan A., Davis A.D., Ghosh A., Jagtap T., Xavier A., Menon A.J., Roy D., Gandhi S., Gregor T. (2023). Guidelines for pharmacotherapy in Alzheimer’s disease–A primer on FDA-approved drugs. J. Neurosci. Rural Pract..

[136] Larkin H.D. (2022). First donepezil transdermal patch approved for Alzheimer disease. JAMA.

[137] Chopade P., Chopade N., Zhao Z., Mitragotri S., Liao R., Chandran Suja V. (2023). Alzheimer’s and Parkinson’s disease therapies in the clinic. Bioeng. Transl. Med..

[138] Kurkinen M. (2023). Lecanemab (Leqembi) is not the right drug for patients with Alzheimer’s disease. Adv. Clin. Exp. Med..

[139] Adepoju V.A., Onyezue O.I., Jamil S., Okesanya O.J., Don E. (2024). Lecanemab Unveiled: Exploring Alzheimer’s Treatment Advancements, Assessing Strengths, Limitations, and Its Therapeutic Landscape Position. Biomed. Environ. Sci..

[140] Robinson D.M., Keating G.M. (2006). Memantine: A review of its use in Alzheimer’s disease. Drugs.

[141] Nguyen K., Hoffman H., Chakkamparambil B., Grossberg G.T. (2021). Evaluation of rivastigmine in Alzheimer’s disease. Neurodegener. Dis. Manag..

[142] Kumar D., Ashraf G.M., Bilgrami A.L., Hassan M.I. (2022). Emerging therapeutic developments in neurodegenerative diseases: A clinical investigation. Drug Discov. Today.

[143] Laifenfeld D., Yanover C., Ozery-Flato M., Shaham O., Rosen-Zvi M., Lev N., Goldschmidt Y., Grossman I. (2021). Emulated clinical trials from longitudinal real-world data efficiently identify candidates for neurological disease modification: Examples from parkinson’s disease. Front. Pharmacol..

[144] Kriebel-Gasparro A. (2016). Parkinson’s disease: Update on medication management. J. Nurse Pract..

[145] Jenner P., Rocha J.-F., Ferreira J.J., Rascol O., Soares-da-Silva P. (2021). Redefining the strategy for the use of COMT inhibitors in Parkinson’s disease: The role of opicapone. Expert Rev. Neurother..

[146] Meglio M. (2023). FDA Issues Complete Response Letter for AbbVie’s 24-Hour Continuous Carbidopa/Levodopa Pump. Neurology Live.

[147] Mehta S.H., Pahwa R., Tanner C.M., Hauser R.A., Johnson R. (2021). Effects of Gocovri (amantadine) extended release capsules on non-motor symptoms in patients with Parkinson’s disease and dyskinesia. Neurol. Ther..

[148] Lipp M.M., Hickey A.J., Langer R., LeWitt P.A. (2021). A technology evaluation of CVT-301 (Inbrija): An inhalable therapy for treatment of Parkinson’s disease. Expert Opin. Drug Deliv..

[149] Agbo F., Isaacson S.H., Gil R., Chiu Y.-Y., Brantley S.J., Bhargava P., Navia B. (2021). Pharmacokinetics and comparative bioavailability of apomorphine sublingual film and subcutaneous apomorphine formulations in patients with Parkinson’s disease and “OFF” episodes: Results of a randomized, three-way crossover, open-label study. Neurol. Ther..

[150] Wilson S.M., Wurst M.G., Whatley M.F., Daniels R.N. (2020). Classics in chemical neuroscience: Pramipexole. ACS Chem. Neurosci..

[151] McFarthing K., Buff S., Rafaloff G., Dominey T., Wyse R.K., Stott S.R. (2020). Parkinson’s disease drug therapies in the clinical trial pipeline: 2020. J. Park. Dis..

[152] St. Onge E., Vanderhoof M., Miller S. (2021). Opicapone (Ongentys): A New COMT Inhibitor for the Treatment of Parkinson’s Disease. Ann. Pharmacother..

[153] Aradi S.D., Hauser R.A. (2020). Medical management and prevention of motor complications in Parkinson’s disease. Neurotherapeutics.

[154] Hauser R.A., Espay A.J., Ellenbogen A.L., Fernandez H.H., Isaacson S.H., LeWitt P.A., Ondo W.G., Pahwa R., Schwarz J., Stocchi F. (2023). IPX203 vs immediate-release carbidopa-levodopa for the treatment of motor fluctuations in Parkinson disease: The RISE-PD randomized clinical trial. JAMA Neurol..

[155] Hansen C.A., Miller D.R., Annarumma S., Rusch C.T., Ramirez-Zamora A., Khoshbouei H. (2022). Levodopa-induced dyskinesia: A historical review of Parkinson’s disease, dopamine, and modern advancements in research and treatment. J. Neurol..

[156] Cowen P.J., Browning M. (2015). What has serotonin to do with depression?. World Psychiatry.

[157] Meneses A. (2013). 5-HT systems: Emergent targets for memory formation and memory alterations. Rev. Neurosci..

[158] Badawy A.A., Dawood S., Bano S. (2023). Kynurenine pathway of tryptophan metabolism in pathophysiology and therapy of major depressive disorder. World J. Psychiatry.

[159] Artigas F. (2013). Serotonin receptors involved in antidepressant effects. Pharmacol. Ther..

[160] Hoyer D., Hannon J.P., Martin G.R. (2002). Molecular, pharmacological and functional diversity of 5-HT receptors. Pharmacol. Biochem. Behav..

[161] de Andrade Teles R.B., Diniz T.C., Costa Pinto T.C., de Oliveira Junior R.G., Gama e Silva M., de Lavor É.M., Fernandes A.W.C., de Oliveira A.P., de Almeida Ribeiro F.P.R., da Silva A.A.M. (2018). Flavonoids as therapeutic agents in Alzheimer’s and Parkinson’s diseases: A systematic review of preclinical evidences. Oxidative Med. Cell. Longev..

[162] Carhart-Harris R.L., Nutt D.J. (2017). Serotonin and brain function: A tale of two receptors. J. Psychopharmacol..

[163] Carvalho A.F., Sharma M.S., Brunoni A.R., Vieta E., Fava G.A. (2016). The safety, tolerability and risks associated with the use of newer generation antidepressant drugs: A critical review of the literature. Psychother. Psychosom..

[164] Kern D.M., Cepeda M.S., Defalco F., Etropolski M. (2020). Treatment patterns and sequences of pharmacotherapy for patients diagnosed with depression in the United States: 2014 through 2019. BMC Psychiatry.

[165] Yu Z., Zhang J., Zheng Y., Yu L. (2020). Trends in antidepressant use and expenditure in six major cities in China from 2013 to 2018. Front. Psychiatry.

[166] Baune B.T., Brignone M., Larsen K.G. (2018). A network meta-analysis comparing effects of various antidepressant classes on the digit symbol substitution test (DSST) as a measure of cognitive dysfunction in patients with major depressive disorder. Int. J. Neuropsychopharmacol..

[167] Mickymaray S. (2019). Efficacy and mechanism of traditional medicinal plants and bioactive compounds against clinically important pathogens. Antibiotics.

[168] Maia M.E., Carvalho M., Sousa Gomes C., Arruda M., Antunes de Magalhães A.J.L., Farias D. (2026). Plant-Derived Peptides with Neuroprotective Activity: Advances and Perspectives in the Prevention of Neurodegenerative Diseases. ACS Omega.

[169] McCutcheon R.A., Marques T.R., Howes O.D. (2020). Schizophrenia—An overview. JAMA Psychiatry.

[170] Carrà G., Crocamo C., Angermeyer M., Brugha T., Toumi M., Bebbington P. (2019). Positive and negative symptoms in schizophrenia: A longitudinal analysis using latent variable structural equation modelling. Schizophr. Res..

[171] Chang C.-Y., Luo D.-Z., Pei J.-C., Kuo M.-C., Hsieh Y.-C., Lai W.-S. (2021). Not just a bystander: The emerging role of astrocytes and research tools in studying cognitive dysfunctions in schizophrenia. Int. J. Mol. Sci..

[172] Ni Y., Zhang W., Sun P., Xu Y., Zhang Q. (2026). Aromatic plant-derived essential oils: Bioactive compounds and their neuroprotective functions in neurological health. J. Food Bioact..

[173] Xie T., Zhang X., Tang X., Zhang H., Yu M., Gong G., Wang X., Evans A., Zhang Z., He Y. (2019). Mapping convergent and divergent cortical thinning patterns in patients with deficit and nondeficit schizophrenia. Schizophr. Bull..

[174] Planchuelo-Gómez Á., Lubeiro A., Núñez-Novo P., Gomez-Pilar J., de Luis-García R., Del Valle P., Martín-Santiago Ó., Pérez-Escudero A., Molina V. (2020). Identificacion of MRI-based psychosis subtypes: Replication and refinement. Prog. Neuro-Psychopharmacol. Biol. Psychiatry.

[175] Madre M., Canales-Rodríguez E.J., Fuentes-Claramonte P., Alonso-Lana S., Salgado-Pineda P., Guerrero-Pedraza A., Moro N., Bosque C., Gomar J.J., Ortíz-Gil J. (2020). Structural abnormality in schizophrenia versus bipolar disorder: A whole brain cortical thickness, surface area, volume and gyrification analyses. NeuroImage Clin..

[176] Song R., Xu H., Dintica C.S., Pan K.-Y., Qi X., Buchman A.S., Bennett D.A., Xu W. (2020). Associations between cardiovascular risk, structural brain changes, and cognitive decline. J. Am. Coll. Cardiol..

[177] Smucny J., Dienel S.J., Lewis D.A., Carter C.S. (2022). Mechanisms underlying dorsolateral prefrontal cortex contributions to cognitive dysfunction in schizophrenia. Neuropsychopharmacology.

[178] Ji J.L., Diehl C., Schleifer C., Tamminga C.A., Keshavan M.S., Sweeney J.A., Clementz B.A., Hill S.K., Pearlson G., Yang G. (2019). Schizophrenia exhibits bi-directional brain-wide alterations in cortico-striato-cerebellar circuits. Cereb. Cortex.

[179] Hawiset T., Inkeaw P. (2020). Effects of Stress and Cortisol on the Brain Behavioral Functions: Mood and Memory. Srinagarind Med. J..

[180] Martínez A.L., Brea J., Rico S., De los Frailes M.T., Loza M.I. (2021). Cognitive deficit in schizophrenia: From etiology to novel treatments. Int. J. Mol. Sci..

[181] Fiocco A.J., D’Amico D., De Beaumont L., Poirier J., Lupien S. (2020). Association between BDNF polymorphism and hypothalamic-pituitary-adrenal activity in later adulthood. Gerontology.

[182] Labad J. (2019). The role of cortisol and prolactin in the pathogenesis and clinical expression of psychotic disorders. Psychoneuroendocrinology.

[183] Duc Nguyen H., Oh H., Yu B.P., Hoang N.M.H., Jo W.H., Young Chung H., Kim M.-S. (2022). Associations between Prolactin, Diabetes, and Cognitive Impairment: A literature review. Neuroendocrinology.

[184] Chien H.-Y., Chen S.-M., Li W.-C. (2023). Dopamine receptor agonists mechanism of actions on glucose lowering and their connections with prolactin actions. Front. Clin. Diabetes Healthc..

[185] Bansal V., Chatterjee I. (2021). Role of neurotransmitters in schizophrenia: A comprehensive study. Kuwait J. Sci..

[186] Teleanu R.I., Niculescu A.-G., Roza E., Vladâcenco O., Grumezescu A.M., Teleanu D.M. (2022). Neurotransmitters—Key factors in neurological and neurodegenerative disorders of the central nervous system. Int. J. Mol. Sci..

[187] Schoonover K.E., Dienel S.J., Lewis D.A. (2020). Prefrontal cortical alterations of glutamate and GABA neurotransmission in schizophrenia: Insights for rational biomarker development. Biomark. Neuropsychiatry.

[188] Shu I.-W., Granholm E.L., Singh F. (2022). Targeting frontal gamma activity with neurofeedback to improve working memory in schizophrenia. Cognitive Functioning in Schizophrenia: Leveraging the RDoC Framework.

[189] Zhang T., Liu C., Zhong N., Wang Y., Huang Y., Zhang X. (2024). Advances in the treatment of cognitive impairment in schizophrenia: Targeting NMDA receptor pathways. Int. J. Mol. Sci..

[190] Billard J.-M. (2018). Changes in serine racemase-dependent modulation of NMDA receptor: Impact on physiological and pathological brain aging. Front. Mol. Biosci..

[191] Pei J.-C., Luo D.-Z., Gau S.-S., Chang C.-Y., Lai W.-S. (2021). Directly and indirectly targeting the glycine modulatory site to modulate NMDA receptor function to address unmet medical needs of patients with schizophrenia. Front. Psychiatry.

[192] Kindler J., Lim C.K., Weickert C.S., Boerrigter D., Galletly C., Liu D., Jacobs K.R., Balzan R., Bruggemann J., O’Donnell M. (2020). Dysregulation of kynurenine metabolism is related to proinflammatory cytokines, attention, and prefrontal cortex volume in schizophrenia. Mol. Psychiatry.

[193] Carvalho C., Vieira-Coelho M.A. (2022). Cannabis induced psychosis: A systematic review on the role of genetic polymorphisms. Pharmacol. Res..

[194] Kruse A.O., Bustillo J.R. (2022). Glutamatergic dysfunction in Schizophrenia. Transl. Psychiatry.

[195] Coyle J.T., Ruzicka W.B., Balu D.T. (2020). Fifty years of research on schizophrenia: The ascendance of the glutamatergic synapse. Am. J. Psychiatry.

[196] Krystal J.H., D’Souza D.C., Mathalon D., Perry E., Belger A., Hoffman R. (2003). NMDA receptor antagonist effects, cortical glutamatergic function, and schizophrenia: Toward a paradigm shift in medication development. Psychopharmacology.

[197] McCutcheon R.A., Krystal J.H., Howes O.D. (2020). Dopamine and glutamate in schizophrenia: Biology, symptoms and treatment. World Psychiatry.

[198] Adell A. (2020). Brain NMDA receptors in schizophrenia and depression. Biomolecules.

[199] Moghaddam B., Javitt D. (2012). From revolution to evolution: The glutamate hypothesis of schizophrenia and its implication for treatment. Neuropsychopharmacology.

[200] Venkataramaiah C., Payani S., Priya B.L., Pradeepkiran J.A. (2021). Therapeutic potentiality of a new flavonoid against ketamine induced glutamatergic dysregulation in schizophrenia: In vivo and in silico approach. Biomed. Pharmacother..

[201] Shi W., Li M., Zhang T., Yang C., Zhao D., Bai J. (2024). GABA system in the prefrontal cortex involved in psychostimulant addiction. Cereb. Cortex.

[202] Chiu C.Q., Barberis A., Higley M.J. (2019). Preserving the balance: Diverse forms of long-term GABAergic synaptic plasticity. Nat. Rev. Neurosci..

[203] Jiménez-Balado J., Eich T.S. (2021). GABAergic dysfunction, neural network hyperactivity and memory impairments in human aging and Alzheimer’s disease. Semin. Cell Dev. Biol..

[204] Liwinski T., Lang U.E., Brühl A.B., Schneider E. (2023). Exploring the therapeutic potential of gamma-aminobutyric acid in stress and depressive disorders through the gut–brain axis. Biomedicines.

[205] Prévot T., Sibille E. (2021). Altered GABA-mediated information processing and cognitive dysfunctions in depression and other brain disorders. Mol. Psychiatry.

[206] Korczak M., Kurowski P., Leśniak A., Grönbladh A., Filipowska A., Bujalska-Zadrożny M. (2020). GABAB receptor intracellular signaling: Novel pathways for depressive disorder treatment?. Eur. J. Pharmacol..

[207] Spiering M.J. (2018). The discovery of GABA in the brain. J. Biol. Chem..

[208] Sanacora G., Yan Z., Popoli M. (2022). The stressed synapse 2.0: Pathophysiological mechanisms in stress-related neuropsychiatric disorders. Nat. Rev. Neurosci..

[209] Kilb W. (2021). When are depolarizing GABAergic responses excitatory?. Front. Mol. Neurosci..

[210] Rocha L., Alonso-Vanegas M., Martínez-Juárez I.E., Orozco-Suárez S., Escalante-Santiago D., Feria-Romero I.A., Zavala-Tecuapetla C., Cisneros-Franco J.M., Buentello-García R.M., Cienfuegos J. (2015). GABAergic alterations in neocortex of patients with pharmacoresistant temporal lobe epilepsy can explain the comorbidity of anxiety and depression: The potential impact of clinical factors. Front. Cell. Neurosci..

[211] Schür R.R., Draisma L.W., Wijnen J.P., Boks M.P., Koevoets M.G., Joels M., Klomp D.W., Kahn R.S., Vinkers C.H. (2016). Brain GABA levels across psychiatric disorders: A systematic literature review and meta-analysis of 1H-MRS studies. Hum. Brain Mapp..

[212] McIntyre R.S., Carvalho I.P., Lui L.M., Majeed A., Masand P.S., Gill H., Rodrigues N.B., Lipsitz O., Coles A.C., Lee Y. (2020). The effect of intravenous, intranasal, and oral ketamine in mood disorders: A meta-analysis. J. Affect. Disord..

[213] Nikolin S., Rodgers A., Schwaab A., Bahji A., Zarate C., Vazquez G., Loo C. (2023). Ketamine for the treatment of major depression: A systematic review and meta-analysis. eClinicalMedicine.

[214] Jiang Y., Peng T., Gaur U., Silva M., Little P., Chen Z., Qiu W., Zhang Y., Zheng W. (2019). Role of corticotropin releasing factor in the neuroimmune mechanisms of depression: Examination of current pharmaceutical and herbal therapies. Front. Cell. Neurosci..

[215] Marwaha S., Palmer E., Suppes T., Cons E., Young A.H., Upthegrove R. (2023). Novel and emerging treatments for major depression. Lancet.

[216] Hofmeijer J., Van Putten M.J. (2012). Ischemic cerebral damage: An appraisal of synaptic failure. Stroke.

[217] Lee S.Y., Kim J.H. (2015). Mechanisms underlying presynaptic Ca^2+^ transient and vesicular glutamate release at a CNS nerve terminal during in vitro ischaemia. J. Physiol..

[218] Molinaro P., Cataldi M., Cuomo O., Viggiano D., Pignataro G., Sirabella R., Secondo A., Boscia F., Pannaccione A., Scorziello A. (2012). Genetically modified mice as a strategy to unravel the role played by the Na^+^/Ca^2+^ exchanger in brain ischemia and in spatial learning and memory deficits. Sodium Calcium Exchange: A Growing Spectrum of Pathophysiological Implications: Proceedings of the 6th International Conference on Sodium Calcium Exchange.

[219] Carlson A.P., Hänggi D., Macdonald R.L., Shuttleworth C.W. (2020). Nimodipine reappraised: An old drug with a future. Curr. Neuropharmacol..

[220] Krasil’nikova I., Surin A., Sorokina E., Fisenko A., Boyarkin D., Balyasin M., Demchenko A., Pomytkin I., Pinelis V. (2019). Insulin protects cortical neurons against glutamate excitotoxicity. Front. Neurosci..

[221] Viejo L., Rubio-Alarcón M., Arribas R.L., Moreno-Castro M., Pérez-Marín R., Braun-Cornejo M., Estrada-Valencia M., de Los Ríos C. (2021). Synthesis and biological assessment of 4, 1-benzothiazepines with neuroprotective activity on the Ca^2+^ overload for the treatment of neurodegenerative diseases and stroke. Molecules.

[222] Wang J., Gan Y., Han P., Yin J., Liu Q., Ghanian S., Gao F., Gong G., Tang Z. (2018). Ischemia-induced neuronal cell death is mediated by chemokine receptor CX3CR1. Sci. Rep..

[223] Tamer C.E., Temel Ş.G., Suna S., Karabacak A.Ö., Özcan T., Ersan L.Y., Kaya B.T., Çopur Ö.U. (2021). Evaluation of bioaccessibility and functional properties of kombucha beverages fortified with different medicinal plant extracts. Turk. J. Agric. For..

[224] Pandey S.N., Rangra N.K., Singh S., Arora S., Gupta V. (2021). Evolving role of natural products from traditional medicinal herbs in the treatment of Alzheimer’s disease. ACS Chem. Neurosci..

[225] Lawate V.N., Kore K.J., Bhagat V., Shete R.V. (2026). The Comprehensive Review of Amlyiod Hypothesis in Alzheimer’s (AD). World.

[226] Howes M.J.R., Perry N.S., Houghton P.J. (2003). Plants with traditional uses and activities, relevant to the management of Alzheimer’s disease and other cognitive disorders. Phytother. Res. Int. J. Devoted Pharmacol. Toxicol. Eval. Nat. Prod. Deriv..

[227] Bayattork M., Rahman M., Hossain M.I., Zhang Y., Haque A.N.M.A., Kim B., Naebe M. (2026). Impact of Textile-Derived Micro-and Nanoplastics on Brain Health: An Emerging Environmental Risk. Environ. Sci. Technol..

[228] Kofoed R.H., Hvingelby V.S., Pineda-Pardo J.A., Blesa J., Paschen S., Tandon A., Joutsa J., Glud A.N. (2026). Focused Ultrasound for the Treatment of Circuit and Molecular Pathology in Parkinson’s Disease. Mov. Disord..

[229] Mythri R.B., Bharath M.M. (2012). Curcumin: A potential neuroprotective agent in Parkinson’s disease. Curr. Pharm. Des..

[230] Zhang X., Cao K., Wan J., Liu Z., Ren Z., Wang W., Wang H. (2026). Neuroprotective effects and mechanism of ginseng aqueous extract against Alzheimer’s disease. Phytomedicine.

[231] Oso T.A., Adeleye S.J., Sikiru B.M., Ganiyu B.W., Owolagba O.A., Okesanya O.J., Ogunwale A., Adeyemi J.D., Akinloye O. (2026). Redox-oxidative stress and micronutrients interplay in the clinical phenotype of schizophrenia in Nigerian subjects. Explor. Neurosci..

[232] Llorca-Bofí V., Parellada E., Morén C., Sellgren C.M., Bioque M. (2026). Neuroinflammation: An unfortunate term to describe schizophrenia. Mol. Psychiatry.

[233] Moosavi F., Hosseini R., Saso L., Firuzi O. (2015). Modulation of neurotrophic signaling pathways by polyphenols. Drug Des. Dev. Ther..

[234] Rs D. (2012). Synaptic dysfunction in depression: Potential therapeutic targets. Science.

[235] Marx W., Penninx B.W., Solmi M., Furukawa T.A., Firth J., Carvalho A.F., Berk M. (2023). Major depressive disorder. Nat. Rev. Dis. Primers.

[236] Lopresti A.L., Maes M., Maker G.L., Hood S.D., Drummond P.D. (2014). Curcumin for the treatment of major depression: A randomised, double-blind, placebo controlled study. J. Affect. Disord..

[237] Sachdev P.S., Bentvelzen A.C., Gustafson D., Hansra G.K., Hosoki S., Jiang J., Lennon M.J., Moro M.A., Saks D.G., Samaras K. (2026). Vascular Cognitive Impairment and Dementia: Clinical Features, Neuropathology, and Biomarkers. J. Am. Coll. Cardiol..

[238] Kim H.J., Kim P., Shin C.Y. (2013). A comprehensive review of the therapeutic and pharmacological effects of ginseng and ginsenosides in central nervous system. J. Ginseng Res..

[239] Spencer J.P. (2009). The impact of flavonoids on memory: Physiological and molecular considerations. Chem. Soc. Rev..

[240] Yurko-Mauro K., McCarthy D., Rom D., Nelson E.B., Ryan A.S., Blackwell A., Salem N., Stedman M., Investigators M. (2010). Beneficial effects of docosahexaenoic acid on cognition in age-related cognitive decline. Alzheimer’s Dement..

[241] Mix J.A., Crews W.D. (2000). An examination of the efficacy of *Ginkgo biloba* extract EGb 761 on the neuropsychologic functioning of cognitively intact older adults. J. Altern. Complement. Med. Paradig. Pract. Policy Adv. Integr. Health.

[242] Dwivedi A., Anjali A., Narzari H., Kumar Y., Sharma H.P., Dubey A., Nilima N., Rajan R., Singh M.B., Vishnu V.Y. (2026). Efficacy of *Bacopa monnieri* (Linn.) on Cognitive Function and Alterations in Blood Metabolites in Patients with Amnestic Mild Cognitive Impairment and Early Alzheimer Disease: Protocol for an Exploratory Double-Blind, Randomized, Placebo-Controlled Trial. JMIR Res. Protoc..

[243] Salari N., Lotfi F., Abdolmaleki A., Heidarian P., Rasoulpoor S., Fazeli J., Najafi H., Mohammadi M. (2025). The global prevalence of mild cognitive impairment in geriatric population with emphasis on influential factors: A systematic review and meta-analysis. BMC Geriatr..

[244] Gregory J., Vengalasetti Y.V., Bredesen D.E., Rao R.V. (2021). Neuroprotective herbs for the management of Alzheimer’s disease. Biomolecules.

[245] Banerjee S., Anand U., Ghosh S., Ray D., Ray P., Nandy S., Deshmukh G.D., Tripathi V., Dey A. (2021). Bacosides from Bacopa monnieri extract: An overview of the effects on neurological disorders. Phytother. Res..

[246] Tandon B., Anand U., Alex B.K., Kaur P., Nandy S., Shekhawat M.S., Sanyal R., Pandey D.K., Koshy E.P., Dey A. (2021). Statistical optimization of in vitro callus induction of wild and cultivated varieties of *Mucuna pruriens* L. (DC.) using response surface methodology and assessment of L-Dopa biosynthesis. Ind. Crops Prod..

[247] Anand U., Jacobo-Herrera N., Altemimi A., Lakhssassi N. (2019). A comprehensive review on medicinal plants as antimicrobial therapeutics: Potential avenues of biocompatible drug discovery. Metabolites.

[248] Schifano F., Catalani V., Sharif S., Napoletano F., Corkery J.M., Arillotta D., Fergus S., Vento A., Guirguis A. (2022). Benefits and harms of ‘smart drugs’ (nootropics) in healthy individuals. Drugs.

[249] Greenfield B. (2020). Boundless: Upgrade Your Brain, Optimize Your Body & Defy Aging.

[250] Suliman N.A., Mat Taib C.N., Mohd Moklas M.A., Adenan M.I., Hidayat Baharuldin M.T., Basir R. (2016). Establishing natural nootropics: Recent molecular enhancement influenced by natural nootropic. Evid.-Based Complement. Altern. Med..

[251] Manetti D., Dei S., Arias H.R., Braconi L., Gabellini A., Teodori E., Romanelli M.N. (2023). Recent advances in the discovery of nicotinic acetylcholine receptor allosteric modulators. Molecules.

[252] Phogat J., Bali A., Kapoor N. (2023). Smart Drugs in Cognitive Disorders. Applications of Synthetic Biology in Health, Energy, and Environment.

[253] Colovic M.B., Krstic D.Z., Lazarevic-Pasti T.D., Bondzic A.M., Vasic V.M. (2013). Acetylcholinesterase inhibitors: Pharmacology and toxicology. Curr. Neuropharmacol..

[254] Fernando W.M.A.D.B., Martins I.J., Goozee K., Brennan C.S., Jayasena V., Martins R.N. (2015). The role of dietary coconut for the prevention and treatment of Alzheimer’s disease: Potential mechanisms of action. Br. J. Nutr..

[255] Molz P., Schröder N. (2017). Potential therapeutic effects of lipoic acid on memory deficits related to aging and neurodegeneration. Front. Pharmacol..

[256] Srivastava A., Srivastava P., Pandey A., Khanna V., Pant A. (2019). Phytomedicine: A potential alternative medicine in controlling neurological disorders. New Look to Phytomedicine.

[257] Kennedy D.O., Scholey A., Wesnes K.A. (2001). Dose dependent changes in cognitive performance and mood following acute administration of Ginseng to healthy young volunteers. Nutr. Neurosci..

[258] Kennedy D.O., Scholey A., Wesnes K.A. (2001). Differential, dose dependent changes in cognitive performance following acute administration of a *Ginkgo biloba*/*Panax ginseng* combination to healthy young volunteers. Nutr. Neurosci..

[259] Dar N.J., Ahmad M. (2020). Neurodegenerative diseases and *Withania somnifera* (L.): An update. J. Ethnopharmacol..

[260] Baddaoui S., Saalaoui E., Khibech O., Salagre D., Fernández-Ochoa Á., Mamri S., Aktary N., Rahman M., Rani A., Asehraou A. (2026). HPLC-ESI-QTOF-MS/MS-Guided Profiling of Bioactive Compounds in Fresh and Stored Saffron Corms Reveals Potent Anticancer Activity Against Colorectal Cancer. Pharmaceuticals.

[261] Guan Y., Tang G., Li L., Shu J., Zhao Y., Huang L., Tang J. (2024). Herbal medicine and gut microbiota: Exploring untapped therapeutic potential in neurodegenerative disease management. Arch. Pharmacal Res..

[262] Zheng S.-Y., Zhou X.-Q. (2025). A perspective on the mechanisms of herbal medicine for cognitive impairment. Front. Neurol..

[263] Abbaszadeh F., Fakhri S., Varnamkhasti B.S., Moradi S.Z., Olfati M.H., Moradi Z., Khirehgesh M.R., Khan H. (2025). Restoring Gut-brain Function by Medicinal Herbs Offering Neuroprotection through Suppressing Inflammatory Pathways: A Systematic Review. Curr. Neuropharmacol..

[264] Ji X., Wang J., Lan T., Zhao D., Xu P. (2025). Gut microbial metabolites and the brain–gut axis in Alzheimer’s disease: A review. Biomol. Biomed..

[265] Koga-Batko J., Antosz-Popiołek K., Suchecki W., Szyller H., Wrześniewska M., Dyda M., Leszek J. (2025). Influence of the Gut Microbiota on the Pathogenesis of Alzheimer’s Disease: A Literature Review. Cells.

[266] Akter K., Hong Y.J., Han I., Choi E.H. (2025). Nonthermal plasma jet mitigates viral replication and inflammation in human coronavirus 229E-infected lung cells by targeting the NF-κB and MAPK pathways. Microb. Pathog..

[267] Tkaczenko H., Buyun L., Kołodziejska R., Kamiński P., Kurhaluk N. (2025). Neuroactive phytochemicals as multi-target modulators of mental health and cognitive function: An integrative review. Int. J. Mol. Sci..

[268] Meng W., Chao W., Kaiwei Z., Sijia M., Jiajia S., Shijie X. (2025). Bioactive compounds from Chinese herbal plants for neurological health: Mechanisms, pathways, and functional food applications. Front. Nutr..

[269] Fu Y., Yang J., Wang X., Yang P., Zhao Y., Li K., Chen Y., Xu Y. (2018). Herbal compounds play a role in neuroprotection through the inhibition of microglial activation. J. Immunol. Res..

[270] Chen C., Wang G.-Q., Li D.-D., Zhang F. (2025). Microbiota–gut–brain axis in neurodegenerative diseases: Molecular mechanisms and therapeutic targets. Mol. Biomed..

[271] Koike A., Takagi T. Gene/protein/family name recognition in biomedical literature. Proceedings of the HLT-NAACL 2004 Workshop: Linking Biological Literature, Ontologies and Databases.

[272] Odia A., Esezobor O.Z. (2017). Therapeutic uses of amino acids. Amino Acid—New Insights and Roles in Plant and Animal.

[273] Bukke V.N., Villani R., Archana M., Wawrzyniak A., Balawender K., Orkisz S., Ferraro L., Serviddio G., Cassano T. (2020). The glucose metabolic pathway as a potential target for therapeutics: Crucial role of glycosylation in Alzheimer’s disease. Int. J. Mol. Sci..

[274] Hatcher H.C., Singh R.N., Torti F.M., Torti S.V. (2009). Synthetic and natural iron chelators: Therapeutic potential and clinical use. Future Med. Chem..

[275] Yan N., Zhang J. (2020). Iron metabolism, ferroptosis, and the links with Alzheimer’s disease. Front. Neurosci..

[276] Ali A.H., Hachem M., Ahmmed M.K. (2024). Docosahexaenoic acid-loaded nanoparticles: A state-of-the-art of preparation methods, characterization, functionality, and therapeutic applications. Heliyon.

[277] Rai S.N., Singh P., Steinbusch H.W., Vamanu E., Ashraf G., Singh M.P. (2021). The role of vitamins in neurodegenerative disease: An update. Biomedicines.

[278] Plantone D., Pardini M., Caneva S., De Stefano N. (2024). Is There a Role of Vitamin D in Alzheimer’s disease?. CNS Neurol. Disord.-Drug Targets.

[279] Mishra S., Grewal J., Wal P., Bhivshet G.U., Tripathi A.K., Walia V. (2024). Therapeutic potential of vasopressin in the treatment of neurological disorders. Peptides.

[280] Szczepanska-Sadowska E. (2024). Neuromodulation of Cardiac Ischemic Pain: Role of the Autonomic Nervous System and Vasopressin. J. Integr. Neurosci..

[281] Harborne J. (1989). Flavonoids. Natural Products of Woody Plants: Chemicals Extraneous to the Lignocellulosic Cell Wall.

[282] Mattioli R., Francioso A., Mosca L., Silva P. (2020). Anthocyanins: A comprehensive review of their chemical properties and health effects on cardiovascular and neurodegenerative diseases. Molecules.

[283] dos Santos N.M., Batista P.B., Batista A.G., Júnior M.R.M. (2019). Current evidence on cognitive improvement and neuroprotection promoted by anthocyanins. Curr. Opin. Food Sci..

[284] Zahi A., Rani A., Aktary N., Rahman M., Mekhfi H., Ziyyat A., Park M.N., Legssyer A., Kim B. (2025). Cardiovascular Effects, Phytochemistry, Drug Interactions, and Safety Profile of *Foeniculum vulgare* Mill. (Fennel): A Comprehensive Review. Pharmaceuticals.

[285] Bindra S., Bose K., Thekkantavida A.C., Alsahli T.G., Pant M., Pappachen L.K., Kim H., Mathew B. (2024). FDA-approved drugs containing dimethylamine pharmacophore: A review of the last 50 years. RSC Adv..

[286] King C., Plakke B. (2024). Maternal choline supplementation modulates cognition and induces anti-inflammatory signaling in the prefrontal cortices of adolescent rats exposed to maternal immune activation. Brain Behav. Immun.-Health.

[287] Cadoná F.C., Weis G.C.C., Assmann C.E., de Oliveira Alves A., Bonadiman B.d.S.R., Machado A.K., Montano M.A.E., da Cruz I.B.M. (2019). Functional and medicinal properties of caffeine-based common beverages. Caffeinated and Cocoa Based Beverages.

[288] Patel J., King A., Malempati M., Patel M. (2024). Understanding nootropics and cognitive enhancement: Mechanism of action and ethical considerations. Health Open Res..

[289] Hachem M., Ahmmed M.K., Nacir-Delord H. (2024). Phospholipidomics in clinical trials for brain disorders: Advancing our understanding and therapeutic potentials. Mol. Neurobiol..

[290] Pethe A., Joshi S., Ali Dar T., Poddar N.K. (2024). Revisiting the role of phospholipases in alzheimer’s: Crosstalk with processed food. Crit. Rev. Food Sci. Nutr..

[291] Mirunalini S., Krishnaveni M. (2010). Therapeutic potential of *Phyllanthus emblica* (amla): The ayurvedic wonder. J. Basic Clin. Physiol. Pharmacol..

[292] Husain I., Zameer S., Madaan T., Minhaj A., Ahmad W., Iqubaal A., Ali A., Najmi A.K. (2019). Exploring the multifaceted neuroprotective actions of *Emblica officinalis* (Amla): A review. Metab. Brain Dis..

[293] Mishra S., Yadav A., Rajan N. (2021). Medicinal uses of Brahmi. Traditional Utilization and Pharmacological Properties of Medicinal Plants.

[294] Dubey T., Chinnathambi S. (2019). Brahmi (*Bacopa monnieri*): An ayurvedic herb against the Alzheimer’s disease. Arch. Biochem. Biophys..

[295] Beevers C.S., Huang S. (2011). Pharmacological and clinical properties of curcumin. Bot. Targets Ther..

[296] Perales-Salinas V., Purushotham S.S., Buskila Y. (2024). Curcumin as a potential therapeutic agent for treating neurodegenerative diseases. Neurochem. Int..

[297] Esmaealzadeh N., Miri M.S., Mavaddat H., Peyrovinasab A., Ghasemi Zargar S., Sirous Kabiri S., Razavi S.M., Abdolghaffari A.H. (2024). The regulating effect of curcumin on NF-κB pathway in neurodegenerative diseases: A review of the underlying mechanisms. Inflammopharmacology.

[298] Rahman M., Akter K., Ahmed K.R., Fahim M.M.H., Aktary N., Park M.N., Shin S.-W., Kim B. (2024). Synergistic Strategies for Castration-Resistant Prostate Cancer: Targeting AR-V7, Exploring Natural Compounds, and Optimizing FDA-Approved Therapies. Cancers.

[299] Akter K., Gul K., Mumtaz S. (2025). Revisiting Curcumin in cancer therapy: Recent insights into molecular Mechanisms, Nanoformulations, and synergistic combinations. Curr. Issues Mol. Biol..

[300] Liu Q., Wang J., Gu Z., Ouyang T., Gao H., Kan H., Yang Y. (2024). Comprehensive exploration of the neuroprotective mechanisms of *Ginkgo biloba* leaves in treating neurological disorders. Am. J. Chin. Med..

[301] Singh B., Kaur P., Singh R., Ahuja P. (2008). Biology and chemistry of *Ginkgo biloba*. Fitoterapia.

[302] Wang Z., Zhang Z., Liu J., Guo M., Li H. (2023). *Panax ginseng* in the treatment of Alzheimer’s disease and vascular dementia. J. Ginseng Res..

[303] Kausar T., Anwar S., Hanan E., Yaseen M., Aboelnaga S.M., Azad Z. (2021). Therapeutic role of ginger (*Zingiber officinale*)—A review. J. Pharm. Res. Int..

[304] Chnadrashekhar C., Sharma S., Mukopadayay S. (2022). A Review on Memory Enhancing Activity of Ginger. Int. J. Health Sci..

[305] Arcusa R., Villaño D., Marhuenda J., Cano M., Cerdà B., Zafrilla P. (2022). Potential role of ginger (*Zingiber officinale* Roscoe) in the prevention of neurodegenerative diseases. Front. Nutr..

[306] Sun B., Wu L., Wu Y., Zhang C., Qin L., Hayashi M., Kudo M., Gao M., Liu T. (2020). Therapeutic potential of *Centella asiatica* and its triterpenes: A review. Front. Pharmacol..

[307] Zweig J.A., Brandes M.S., Brumbach B.H., Caruso M., Wright K.M., Quinn J.F., Soumyanath A., Gray N.E. (2021). Prolonged treatment with *Centella asiatica* improves memory, reduces amyloid-β pathology, and activates NRF2-regulated antioxidant response pathway in 5xFAD Mice. J. Alzheimer’s Dis..

[308] Thakurdesai P.A. (2021). *Centella asiatica* (Gotu kola) leaves: Potential in neuropsychiatric conditions. Nutraceuticals in Brain Health and Beyond.

[309] Azami S., Forouzanfar F. (2024). Therapeutic potentialities of green tea (*Camellia sinensis*) in ischemic stroke: Biochemical and molecular evidence. Metab. Brain Dis..

[310] Pandit N., Kulkarni S., Singhvi G. (2024). Effect of green tea on human brain health. Nutraceutical Fruits and Foods for Neurodegenerative Disorders.

[311] Krishna K., Jigar B., Jagruti P. (2009). Guduchi (*Tinospora cordifolia*): Biological and Medicinal properties, a review. Internet J. Altern. Med..

[312] Singh R., Bhattacharyya C., Prashar V., Arora T., Sharma A., Changotra H., Parkash J. (2023). *Tinospora cordifolia*: A potential neuroprotective agent against various neurodegenerative diseases. J. Herb. Med..

[313] Saha S., Ghosh S. (2012). *Tinospora cordifolia*: One plant, many roles. Anc. Sci. Life.

[314] Al Housseini I., Dakdouk H., El Natour H., Borjac J. (2026). Dual Neuroprotective and Nephroprotective Effects of *Mucuna pruriens*, *Moringa oleifera*, and *Silybum marianum* (Milk Thistle) via Modulation of PI3K/AKT/mTOR and Nrf2/NF-κB Pathways in a Murine Comorbid PD–AKI Model. Int. J. Mol. Sci..

[315] Fakurazi S., Hairuszah I., Nanthini U. (2008). *Moringa oleifera* Lam prevents acetaminophen induced liver injury through restoration of glutathione level. Food Chem. Toxicol..

[316] Aljadaan A.M., AlSaadi A.M., Shaikh I.A., Whitby A., Ray A., Kim D.-H., Carter W.G. (2025). Characterization of the Anticholinesterase and Antioxidant Properties of Phytochemicals from Moringa oleifera as a Potential Treatment for Alzheimer’s Disease. Biomedicines.

[317] Lee Y.-J., Lee Y.M., Lee C.-K., Jung J.K., Han S.B., Hong J.T. (2011). Therapeutic applications of compounds in the Magnolia family. Pharmacol. Ther..

[318] Zhu S., Liu F., Zhang R., Xiong Z., Zhang Q., Hao L., Chen S. (2022). Neuroprotective potency of neolignans in *Magnolia officinalis* cortex against brain disorders. Front. Pharmacol..

[319] Lopresti A.L., Smith S.J., Malvi H., Kodgule R. (2019). An investigation into the stress-relieving and pharmacological actions of an ashwagandha (*Withania somnifera*) extract: A randomized, double-blind, placebo-controlled study. Medicine.

[320] Kumar P. (2026). Neurotherapeutic Potential of *Withania somnifera*: Insights from Ayurvedic Medicine. Ashwagandha.

[321] Lopresti A.L., Smith S.J. (2021). Ashwagandha (*Withania somnifera*) for the treatment and enhancement of mental and physical conditions: A systematic review of human trials. J. Herb. Med..

[322] Pérez-Gómez J., Villafaina S., Adsuar J.C., Merellano-Navarro E., Collado-Mateo D. (2020). Effects of Ashwagandha (*Withania somnifera*) on VO_2max_: A systematic review and meta-analysis. Nutrients.

[323] Pradeep H.L.N.R., Perera P.K., Waratenne P.R., Samaranayake N., Dissanayake W.D.N. (2026). Effects of *Bacopa monnieri* herbal supplement on aging and neurocognitive functions, including neurophysiological assessments, in relation to constitution (*Prakriti*) in healthy adults: Clinical trial protocol. Front. Med..

[324] Feng J.-X., Zheng M.-Q., Tian X., Zimmermann A., Wang A.-X., Meng X. (2025). *Ginkgo biloba* extract EGb 761 in patients with dementia and a history of cerebral infarction—Meta-analysis of pooled data from randomised clinical trials. Front. Neurol..

[325] Riepe M., Hoerr R., Schlaefke S. (2025). *Ginkgo biloba* extract EGb 761 is safe and effective in the treatment of mild dementia–a meta-analysis of patient subgroups in randomised controlled trials. World J. Biol. Psychiatry.

[326] Herrlinger K.A., Nieman K.M., Sanoshy K.D., Fonseca B.A., Lasrado J.A., Schild A.L., Maki K.C., Wesnes K.A., Ceddia M.A. (2018). Spearmint extract improves working memory in men and women with age-associated memory impairment. J. Altern. Complement. Med..

[327] Howes M.J.R., Perry N.S., Vásquez-Londoño C., Perry E.K. (2020). Role of phytochemicals as nutraceuticals for cognitive functions affected in ageing. Br. J. Pharmacol..

[328] Yang X., Nomoto K., Tohda C. (2021). Diosgenin content is a novel criterion to assess memory enhancement effect of yam extracts. J. Nat. Med..

[329] Inada Y., Tohda C., Sasabayashi D., Suzuki M. (2024). Diosgenin-rich Yam (rhizome of *Dioscorea batatas*) extract ameliorates cognitive functions and plasma biomarkers for mild cognitive impairment and mild Alzheimer’s disease: A randomized controlled trial. Phytomed. Plus.

[330] Nowell J., Crook H., de Leon M.J., Edison P. (2026). Advances in the drug treatment of Alzheimer’s disease: Pathophysiology and mechanisms of action. BMJ.

[331] Reiss A.B., Gulkarov S., Jacob B., Srivastava A., Pinkhasov A., Gomolin I.H., Stecker M.M., Wisniewski T., De Leon J. (2024). Mitochondria in Alzheimer’s disease pathogenesis. Life.

[332] Lanzillotta S., Rolfi L.R., Zulli B., Barone E. (2026). Metabolic breakdown: Linking insulin resistance and mitochondrial dysfunction to neurodegeneration in Alzheimer’s disease. Neural Regen. Res..

[333] Chen J., Liu B., Yao X., Yang X., Sun J., Yi J., Xue F., Zhang J., Shen Y., Chen B. (2025). AMPK/SIRT1/PGC-1α Signaling Pathway: Molecular Mechanisms and Targeted Strategies From Energy Homeostasis Regulation to Disease Therapy. CNS Neurosci. Ther..

[334] Pickrell A.M., Youle R.J. (2015). The roles of PINK1, parkin, and mitochondrial fidelity in Parkinson’s disease. Neuron.

[335] Palikaras K., Lionaki E., Tavernarakis N. (2016). Mitophagy: In sickness and in health. Mol. Cell. Oncol..

[336] Price N.L., Gomes A.P., Ling A.J., Duarte F.V., Martin-Montalvo A., North B.J., Agarwal B., Ye L., Ramadori G., Teodoro J.S. (2012). SIRT1 is required for AMPK activation and the beneficial effects of resveratrol on mitochondrial function. Cell Metab..

[337] Islam M.R., Rauf A., Akter S., Akter H., Al-Imran M.I.K., Fakir M.N.H., Thufa G.K., Islam M.T., Hemeg H.A., Abdulmonem W.A. (2025). Neuroprotective potential of curcumin in neurodegenerative diseases: Clinical insights into cellular and molecular signaling pathways. J. Biochem. Mol. Toxicol..

[338] Akter K., Lim J.S., Choi E.H. (2026). Investigating the Synergistic Effect of Nitric Oxide Plasma-treated Water and Curcumin on Apoptosis in Lung Cancer Cells via intrinsic and ATR/ATM/p53-Dependent Pathways: An In Vitro Study. Plasma Chem. Plasma Process..

[339] Ahn C.-H., Myong J.S., Ahmed K.R., Rahman M.A., Fahim M.M.H., Choi M., Rahman M., Choi J., Kim K., Moon S. (2025). A pharmacoinformatic approach for studying Atractylodes Lancea DC’s anticancer potential and control ROS-mediated apoptosis against prostate cancer cells. Front. Oncol..

[340] Estremor-Rodríguez R., Redondo-Barrera A., Gil-Cure S., Garcés-Barraza J., Contreras-Puentes N. (2026). Use of curcumin as a potential therapeutic agent in Alzheimer’s disease: A systematic review. J. Pharm. Pharmacogn. Res..

[341] Huang Z., Zheng Y., Fan Z., Wei Z., Cai J. (2026). Effects and mechanisms of ginsenoside Rg1 in alleviating hypothalamic oxidative stress and metabolic dysfunction via Nrf2-Tyrosine metabolism axis. Mol. Cell. Endocrinol..

[342] Oriquat G., Abdulsahib W.K., Jyothi S.R., Nayak P.P., Chauhan A.S., Singla S., Sead F.F., Polatova D. (2026). miR-132 and Its Exosomal Form in Alzheimer’s Disease: Linking Epigenetic Regulation to Neurodegeneration. Mol. Neurobiol..

[343] Wu Y., Dou J., Wan X., Leng Y., Liu X., Chen L., Shen Q., Zhao B., Meng Q., Hou J. (2019). Histone deacetylase inhibitor MS-275 alleviates postoperative cognitive dysfunction in rats by inhibiting hippocampal neuroinflammation. Neuroscience.

[344] Ma Y., Wang W., Liu S., Qiao X., Xing Y., Zhou Q., Zhang Z. (2023). Epigenetic regulation of neuroinflammation in Alzheimer’s disease. Cells.

[345] Qazi T.J., Quan Z., Mir A., Qing H. (2018). Epigenetics in Alzheimer’s disease: Perspective of DNA methylation. Mol. Neurobiol..

[346] Zingale V.D., Gugliandolo A., Mazzon E. (2021). MiR-155: An important regulator of neuroinflammation. Int. J. Mol. Sci..

[347] Çelik H., Dalkılınç E., Aydın Ş., Çelik O., Küçükler S., Topal A., Akay R., Gönüllü S., Yıldız M.O., Alım B. (2026). Delivery of miR-25802 via Small Vesicles Protects Against Mitochondrial Injury, Oxidative Stress, and Neuroinflammation in Alzheimer’s Disease. Mol. Neurobiol..

[348] Park J.-Y., Sohn H.-Y., Koh Y.H., Jo C. (2021). Curcumin activates Nrf2 through PKCδ-mediated p62 phosphorylation at Ser351. Sci. Rep..

[349] Islam F., Nafady M.H., Islam M.R., Saha S., Rashid S., Akter A., Or-Rashid M.H., Akhtar M.F., Perveen A., Md Ashraf G. (2022). Resveratrol and neuroprotection: An insight into prospective therapeutic approaches against Alzheimer’s disease from bench to bedside. Mol. Neurobiol..

[350] Merlo S., Lipari C.L.R., Patti A., Sortino M.A. (2026). Microglial Activation Under Hypoxic Conditions in Early Alzheimer’s Disease: Can Natural SIRT1 Activators Be Therapeutic Allies in the Inflammation–Energy Axis?. Phytother. Res..

[351] Yang X., Zhou P., Zhao Z., Li J., Fan Z., Li X., Cui Z., Fu A. (2023). Improvement effect of mitotherapy on the cognitive ability of Alzheimer’s disease through NAD^+^/SIRT1-mediated autophagy. Antioxidants.

[352] Wang R., Wu Y., Liu R., Liu M., Li Q., Ba Y., Huang H. (2022). Deciphering therapeutic options for neurodegenerative diseases: Insights from SIRT1. J. Mol. Med..

[353] Wu W.-F., Chen C., Lin J.-T., Jiao X.-H., Dong W., Wan J., Liu Q., Qiu Y.-K., Sun A., Liu Y.-Q. (2024). Impaired synaptic plasticity and decreased glutamatergic neuron excitability induced by SIRT1/BDNF downregulation in the hippocampal CA1 region are involved in postoperative cognitive dysfunction. Cell. Mol. Biol. Lett..

[354] Qian X.-H., Xie R.-Y., Liu X.-L., Chen S.-D., Tang H.-D. (2022). Mechanisms of short-chain fatty acids derived from gut microbiota in Alzheimer’s disease. Aging Dis..

[355] Haq S., Grondin J.A., Khan W.I. (2021). Tryptophan-derived serotonin-kynurenine balance in immune activation and intestinal inflammation. FASEB J..

[356] Ghosh S.S., Wang J., Yannie P.J., Ghosh S. (2020). Intestinal barrier dysfunction, LPS translocation, and disease development. J. Endocr. Soc..

[357] Koemel N.A., Senior A.M., Celermajer D.S., Grech A., Gill T.P., Simpson S.J., Raubenheimer D., Skilton M.R. (2023). Multi-nutrient analysis of dietary macronutrients with all-cause, cardiovascular, and cancer mortality: Data from NHANES 1999–2014. Nutrients.

[358] Iqbal H., Kim Y., Jin M., Rhee D.-K. (2025). Ginseng as a therapeutic target to alleviate gut and brain diseases via microbiome regulation. J. Ginseng Res..

[359] Scazzocchio B., Minghetti L., D’Archivio M. (2020). Interaction between gut microbiota and curcumin: A new key of understanding for the health effects of curcumin. Nutrients.

[360] Morato-Martínez M., López-Plaza B., Santurino C., Palma-Milla S., Gómez-Candela C. (2020). A dairy product to reconstitute enriched with bioactive nutrients stops bone loss in high-risk menopausal women without pharmacological treatment. Nutrients.

[361] Mouchtoglou C., Cherlet M., Dehau T., Aluwe M., Ducatelle R., Goossens E., Croubels S., Van Immerseel F. (2025). A Low Dose of Berberine Is Metabolized in Weaned Piglets Without Major Changes to Gut Morphology or Gut Microbiota. Animals.

[362] Chen B., Yu X., Zhang L., Huang W., Lyu H., Xu Y., Shen J., Yuan W., Fang M., Li M. (2023). Clinical efficacy of Jingyin granules, a Chinese patent medicine, in treating patients infected with coronavirus disease 2019. Phytomedicine.

[363] Wang H., Zhao T., Liu Z., Danzengquzhen, Cisangzhuoma, Ma J., Li X., Huang X., Li B. (2023). The neuromodulatory effects of flavonoids and gut Microbiota through the gut-brain axis. Front. Cell. Infect. Microbiol..

[364] Shen C.-L., Santos J.M., Elmassry M.M., Chen F., Ji G., Presto P., Kiritoshi T., Liu X., Neugebauer V. (2025). Crosstalk Among Gut Microbiota, Fecal Metabolites, and Amygdala Neuropathology Genes After Ginger Polyphenol Administration in Female Rats with Neuropathic Pain: Evidence for Microbiota–Gut–Brain Connection. Nutrients.

[365] Shang A., Cao S.-Y., Xu X.-Y., Gan R.-Y., Tang G.-Y., Corke H., Mavumengwana V., Li H.-B. (2019). Bioactive compounds and biological functions of garlic (*Allium sativum* L.). Foods.

[366] Cryan J.F., Dinan T.G. (2012). Mind-altering microorganisms: The impact of the gut microbiota on brain and behaviour. Nat. Rev. Neurosci..

[367] Panwar S., Uniyal P., Kukreti N., Hashmi A., Verma S., Arya A., Joshi G. (2024). Role of autophagy and proteostasis in neurodegenerative diseases: Exploring the therapeutic interventions. Chem. Biol. Drug Des..

[368] Le Guerroué F., Youle R.J. (2021). Ubiquitin signaling in neurodegenerative diseases: An autophagy and proteasome perspective. Cell Death Differ..

[369] Yu X., Ni Q., Han L., Zhang S., Xu H., Xie J., Liu Y. (2026). Decode the Ubiquitinome in Parkinson’s Disease: From Pathological Aggregates to Targeted DUB Therapeutics. Neurosci. Bull..

[370] Pohl C., Dikic I. (2019). Cellular quality control by the ubiquitin-proteasome system and autophagy. Science.

[371] Mueed Z., Tandon P., Maurya S.K., Deval R., Kamal M.A., Poddar N.K. (2019). Tau and mTOR: The hotspots for multifarious diseases in Alzheimer’s development. Front. Neurosci..

[372] Nourbakhsh F., Read M.I., Barreto G.E., Sahebkar A. (2020). Boosting the autophagy-lysosomal pathway by phytochemicals: A potential therapeutic strategy against Alzheimer’s disease. IUBMB Life.

[373] Cuanalo-Contreras K., Moreno-Gonzalez I. (2019). Natural products as modulators of the proteostasis machinery: Implications in neurodegenerative diseases. Int. J. Mol. Sci..

[374] Wang C., Yu J.-T., Miao D., Wu Z.-C., Tan M.-S., Tan L. (2014). Targeting the mTOR signaling network for Alzheimer’s disease therapy. Mol. Neurobiol..

[375] Selkoe D.J. (2002). Alzheimer’s disease is a synaptic failure. Science.

[376] Abuelezz S.A., Hendawy N. (2023). Spotlight on Coenzyme Q10 in scopolamine-induced Alzheimer’s disease: Oxidative stress/PI3K/AKT/GSK 3ß/CREB/BDNF/TrKB. J. Pharm. Pharmacol..

[377] Yoshii A., Constantine-Paton M. (2010). Postsynaptic BDNF-TrkB signaling in synapse maturation, plasticity, and disease. Dev. Neurobiol..

[378] Bortolotto V.C., Dahleh M.M.M., Marques L.S., Borstmann S.M.A., Viana C.E., Pinheiro F.C., Balok F.R.M., Meichtry L.B., Boeira S.P., Guerra G.P. (2025). Chrysin modulates the BDNF/TrkB/AKT/Creb neuroplasticity signaling pathway: Acting in the improvement of cognitive flexibility and declarative, working and aversive memory deficits caused by hypothyroidism in C57BL/6 female mice. Neuroscience.

[379] Adeeba, Razi U., Rahman A. (2026). Phytochemicals in Neurodegenerative Diseases. Nourishing the Brain: Diet and Nutrition Strategies in Managing Neurological Disorders.

[380] Cipriano G.L., Raffaele I., Floramo A., Argento V., Astorino M.F., Lui M., Calabrò M., Anchesi I. (2026). Phytochemical and Fungal Bioactive Compounds in the “Brain Health Triad”: A Narrative Review on Neurostimulating, Neurotrophic, and Neuroprotective Synergy. Int. J. Mol. Sci..

[381] Akter K., Kim Y., Choi E.H., Han I. (2024). Nonthermal biocompatible plasma in stimulating osteogenic differentiation by targeting p38/FOXO1 and PI3K/AKT pathways in hBMSCs. J. Biol. Eng..

[382] Karad V., Gupta G.L. (2025). Phytochemicals encouraging neurotrophic pathways: Brain-derived neurotrophic factors as molecular targets in depression. Naunyn-Schmiedeberg’s Arch. Pharmacol..

[383] Xiao X., Yan X., Chunhua L., Yang Y. (2026). Metabolic dysfunction and mitochondrial failure in Alzheimer’s disease: Integrating pathophysiology, clinical evidence and emerging interventions. Front. Neurol..

[384] Xiang Y., Gu Q., Liu D. (2025). Brain endothelial cells in blood–brain barrier regulation and neurological therapy. Int. J. Mol. Sci..

[385] Alkhalifa A.E., Al-Ghraiybah N.F., Odum J., Shunnarah J.G., Austin N., Kaddoumi A. (2023). Blood–brain barrier breakdown in Alzheimer’s disease: Mechanisms and targeted strategies. Int. J. Mol. Sci..

[386] Liu Y., Chen Z., Li A., Liu R., Yang H., Xia X. (2022). The phytochemical potential for brain disease therapy and the possible nanodelivery solutions for brain access. Front. Oncol..

[387] Tsai M.-M., Chen J.-L., Lee T.-H., Liu H., Shanmugam V., Hsieh H.-L. (2022). Brain protective effect of resveratrol via ameliorating interleukin-1β-induced MMP-9-mediated disruption of ZO-1 arranged integrity. Biomedicines.

[388] Gowrikumar S., Tarudji A., McDonald B.Z., Balusa S.S., Kievit F.M., Dhawan P. (2025). Claudin-1 impairs blood–brain barrier by downregulating endothelial junctional proteins in traumatic brain injury. Tissue Barriers.

[389] Godos J., Carota G., Caruso G., Micek A., Frias-Toral E., Giampieri F., Brito-Ballester J., Velasco C.L.R., Quiles J.L., Battino M. (2025). Molecular mechanisms underlying the neuroprotective effects of polyphenols: Implications for cognitive function. EXCLI J..

[390] Naim A., Farooqui A.M., Badruddeen, Khan M.I., Akhtar J., Ahmad A., Ashique S., Islam A. (2026). Nanoengineered phytochemicals overcome blood–brain barrier constraints in neurodegenerative disorders. Front. Neurol..

[391] Naqvi S., Panghal A., Flora S. (2020). Nanotechnology: A promising approach for delivery of neuroprotective drugs. Front. Neurosci..

[392] Batool M., Ain Q.U., Rauf S., David M., Jalil S., Afzal M., Zulfiqar S., Bibi M. (2025). Nanotechnology-Based Delivery of Natural Products. Natural Products in Biomedical Research.

[393] Chang C.-W., Shao E., Mucke L. (2021). Tau: Enabler of diverse brain disorders and target of rapidly evolving therapeutic strategies. Science.

[394] Calabrò M., Rinaldi C., Santoro G., Crisafulli C. (2021). The biological pathways of Alzheimer disease: A review. AIMS Neurosci..

[395] Aktary N., Jeong Y., Oh S., Shin Y., Sung Y., Rahman M., Ramos Santiago L., Choi J., Song H.G., Nurkolis F. (2025). Unveiling the therapeutic potential of natural products in Alzheimer’s disease: Insights from in vitro, in vivo, and clinical studies. Front. Pharmacol..

[396] Barkat M.A., Goyal A., Barkat H.A., Salauddin M., Pottoo F.H., Anwer E.T. (2021). Herbal medicine: Clinical perspective and regulatory status. Comb. Chem. High Throughput Screen..

[397] Singh A.K., Rai S.N., Maurya A., Mishra G., Awasthi R., Shakya A., Chellappan D.K., Dua K., Vamanu E., Chaudhary S.K. (2021). Therapeutic potential of phytoconstituents in management of Alzheimer’s disease. Evid.-Based Complement. Altern. Med..

[398] Alzobaidi N., Quasimi H., Emad N.A., Alhalmi A., Naqvi M. (2021). Bioactive compounds and traditional herbal medicine: Promising approaches for the treatment of dementia. Degener. Neurol. Neuromuscul. Dis..

[399] Bachurin S.O., Bovina E.V., Ustyugov A.A. (2017). Drugs in clinical trials for Alzheimer’s disease: The major trends. Med. Res. Rev..

[400] Tohda C., Yang X., Matsui M., Inada Y., Kadomoto E., Nakada S., Watari H., Shibahara N. (2017). Diosgenin-rich yam extract enhances cognitive function: A placebo-controlled, randomized, double-blind, crossover study of healthy adults. Nutrients.

[401] Lee W.-J., Shin Y.-W., Kim D.-E., Kweon M.-H., Kim M. (2020). Effect of desalted Salicornia europaea L. ethanol extract (PM-EE) on the subjects complaining memory dysfunction without dementia: A 12 week, randomized, double-blind, placebo-controlled clinical trial. Sci. Rep..

[402] Choudhary D., Bhattacharyya S., Bose S. (2017). Efficacy and safety of Ashwagandha (*Withania somnifera* (L.) Dunal) root extract in improving memory and cognitive functions. J. Diet. Suppl..

[403] Kennedy D., Wightman E., Khan J., Grothe T., Jackson P. (2019). The acute and chronic cognitive and cerebral blood-flow effects of nepalese pepper (*Zanthoxylum armatum* dc.) extract—A randomized, double-blind, placebo-controlled study in healthy humans. Nutrients.

[404] Ali D., Verma S., Malviya R., Mishra S., Sundram S. (2024). Implications of herbal components in the treatment of neurological disorders. Curr. Nutr. Food Sci..

[405] Zhang W., Xiao D., Mao Q., Xia H. (2023). Role of neuroinflammation in neurodegeneration development. Signal Transduct. Target. Ther..

[406] Chen G., Su Y., Chen S., Lin T., Lin X. (2025). Polyphenols and Alzheimer’s Disease: A Review on Molecular and Therapeutic Insights with In Silico Support. Food Sci. Nutr..

[407] Lehoczki A., Fekete M., Jarecsny T., Zábó V., Szappanos Á., Csípő T., Lipécz Á., Major D., Fazekas-Pongor V., Varga P. (2025). The Neuroprotective Role of Curcumin: From Molecular Pathways to Clinical Translation—A Narrative Review. Nutrients.

[408] Bučević Popović V., Karahmet Farhat E., Banjari I., Jeličić Kadić A., Puljak L. (2024). Bioavailability of oral curcumin in systematic reviews: A methodological study. Pharmaceuticals.

[409] Wang J., Liu T., Chen P., Yin D., Zhang H., Qiu X., Zou S., Li W. (2025). Pharmacokinetic evaluation of two oral Resveratrol formulations in a randomized, open-label, crossover study in healthy fasting subjects. Sci. Rep..

[410] Al Mamun A., Shao C., Geng P., Wang S., Xiao J. (2024). Polyphenols targeting NF-κB pathway in neurological disorders: What we know so far?. Int. J. Biol. Sci..

[411] Aldekhail N.M., Khojah H., Alsaadoun N.H., Al-Sanea M.M., Alshammari S.B., Alhazeemi A.H., Aldekhail A.M., Aldekhail K.M., Alazmi B.H., Alrayes R.A. (2025). Herbal Medicines in Autism Spectrum Disorder: Therapeutic Potential, Plant Components, and Dosage Guidelines. Altern. Ther. Health Med..

[412] Monagas M., Brendler T., Brinckmann J., Dentali S., Gafner S., Giancaspro G., Johnson H., Kababick J., Ma C., Oketch-Rabah H. (2022). Understanding plant to extract ratios in botanical extracts. Front. Pharmacol..

[413] Liang N., Chen Y., Yang S., Liang C., Gao L., Wang S., Wang Y., Zhang Z., Shi N. (2022). Chinese herbal medicine for mild cognitive impairment: A systematic review of randomized controlled trials. Front. Neurol..

[414] Cottart C.H., Nivet-Antoine V., Laguillier-Morizot C., Beaudeux J.L. (2010). Resveratrol bioavailability and toxicity in humans. Mol. Nutr. Food Res..

[415] Paliwal H., Prajapati B.G., Srichana T. (2025). Nanotechnology-Based Drug Delivery Systems for the Treatment of Neurodegenerative Disorders. Nanomedicine for Neurodegenerative Disorders.

[416] Izzo A.A., Ernst E. (2009). Interactions between herbal medicines and prescribed drugs: An updated systematic review. Drugs.

[417] Tan M.-S., Yu J.-T., Tan C.-C., Wang H.-F., Meng X.-F., Wang C., Jiang T., Zhu X.-C., Tan L. (2014). Efficacy and adverse effects of ginkgo biloba for cognitive impairment and dementia: A systematic review and meta-analysis. J. Alzheimer’s Dis..

[418] Shanaida M., Oleshchuk O., Shevchuk O., Posokhova K., Ivankiv Y., Mocherniuk K., Klantsa M., Korda M. (2026). Herbal Medicines and Drugs Interactions: Cytochrome P450 Responsibility. Curr. Med. Chem..

[419] Sidahmed T.S.M., Hassan A.A.E., El-Haj A.-R.M.O.K., Almosilhy N.A., Mahmoud S.A.S., Mohammed M.O.O., Ali A.E.O., Hassan F.A.O., Ibrahim W.S.M., Abedalla M.M.H. (2026). Patterns of herbal medicine utilization for hypertension during the Sudanese crisis of 2025. Sci. Rep..

[420] Bakare O.A., Okonkwo C.N., Ibrahim A.Y., Adesanya F.O. (2025). Liver failure induced by hepatotoxic drugs: Prevention and treatment strategies. Int. J. Hepatol. Sci..

[421] Dubey A., Ghosh N.S., Agnihotri N., Kumar A., Pandey M., Nishad S. (2022). Herbs Derived Bioactive Compounds and their Potential for the Treatment of Neurological Disorders. Clin. Schizophr. Relat. Psychoses.

[422] Mohd Sairazi N.S., Sirajudeen K. (2020). Natural products and their bioactive compounds: Neuroprotective potentials against neurodegenerative diseases. Evid.-Based Complement. Altern. Med..

[423] Camilo C.J., Leite D.O., da S. Mendes J.W., Dantas A.R., de Carvalho N.K., Castro J.W., Salazar G.J., Ferreira M.K.A., de Meneses J.E.A., da Silva A.W. (2022). Analysis toxicity by different methods and anxiolytic effect of the aqueous extract Lippia sidoides Cham. Sci. Rep..

[424] Elshafie H.S., Camele I., Mohamed A.A. (2023). A comprehensive review on the biological, agricultural and pharmaceutical properties of secondary metabolites based-plant origin. Int. J. Mol. Sci..

[425] Paul S., Chakraborty S., Anand U., Dey S., Nandy S., Ghorai M., Saha S.C., Patil M.T., Kandimalla R., Proćków J. (2021). *Withania somnifera* (L.) Dunal (Ashwagandha): A comprehensive review on ethnopharmacology, pharmacotherapeutics, biomedicinal and toxicological aspects. Biomed. Pharmacother..

[426] Zahiruddin S., Basist P., Parveen A., Parveen R., Khan W., Ahmad S. (2020). Ashwagandha in brain disorders: A review of recent developments. J. Ethnopharmacol..

[427] Mikulska P., Malinowska M., Ignacyk M., Szustowski P., Nowak J., Pesta K., Szeląg M., Szklanny D., Judasz E., Kaczmarek G. (2023). Ashwagandha (*Withania somnifera*)—Current research on the health-promoting activities: A narrative review. Pharmaceutics.

[428] Srikantha S., Jain A. (2024). Investigation of water-soluble coenzyme-Q10 combined with root extract of ashwagandha as a potential therapy for Alzheimer’s Disease. UWill Discover Student Research Conference.

[429] Lerose V., Ponticelli M., Benedetto N., Carlucci V., Lela L., Tzvetkov N.T., Milella L. (2024). *Withania somnifera* (L.) Dunal, a Potential Source of Phytochemicals for Treating Neurodegenerative Diseases: A Systematic Review. Plants.

[430] Singh H., Dhawan B. (1997). Neuropsychopharmacological effects of the ayurvedic nootroplc *Bacopa monnlera linn*. (Brahmi). Indian J. Pharmacol..

[431] Valotto Neto L.J., Reverete de Araujo M., Moretti Junior R.C., Mendes Machado N., Joshi R.K., dos Santos Buglio D., Barbalho Lamas C., Direito R., Fornari Laurindo L., Tanaka M. (2024). Investigating the neuroprotective and cognitive-enhancing effects of Bacopa monnieri: A systematic review focused on inflammation, oxidative stress, mitochondrial dysfunction, and apoptosis. Antioxidants.

[432] Chaudhari K.S., Tiwari N.R., Tiwari R.R., Sharma R.S. (2017). Neurocognitive effect of nootropic drug Brahmi (Bacopa monnieri) in Alzheimer’s disease. Ann. Neurosci..

[433] Delfan M., Kordestani-Moghaddam P., Gholami M., Kazemi K., Mohammadi R. (2024). Evaluating the effects of Bacopa monnieri on cognitive performance and sleep quality of patients with mild cognitive impairment: A triple-blinded, randomized, placebo-controlled trial. Explore.

[434] Shalini V.T., Neelakanta S.J., Sriranjini J.S. (2021). Neuroprotection with Bacopa monnieri—A review of experimental evidence. Mol. Biol. Rep..

[435] Batiha G.E.-S., Magdy Beshbishy A., Wasef L., Elewa Y.H., Abd El-Hack M.E., Taha A.E., Al-Sagheer A.A., Devkota H.P., Tufarelli V. (2020). *Uncaria tomentosa* (Willd. ex Schult.) DC.: A review on chemical constituents and biological activities. Appl. Sci..

[436] Xu Q.-Q., Shaw P.C., Hu Z., Yang W., Ip S.-P., Xian Y.-F., Lin Z.-X. (2021). Comparison of the chemical constituents and anti-Alzheimer’s disease effects of Uncaria rhynchophylla and Uncaria tomentosa. Chin. Med..

[437] Baker K., Marcus C.B., Huffman K., Kruk H., Malfroy B., Doctrow S.R. (1998). Synthetic combined superoxide dismutase/catalase mimetics are protective as a delayed treatment in a rat stroke model: A key role for reactive oxygen species in ischemic brain injury. J. Pharmacol. Exp. Ther..

[438] Kirisattayakul W., Wattanathorn J., Tong-Un T., Muchimapura S., Wannanon P., Jittiwat J. (2013). Cerebroprotective effect of Moringa oleifera against focal ischemic stroke induced by middle cerebral artery occlusion. Oxidative Med. Cell. Longev..

[439] Gopalakrishnan L., Doriya K., Kumar D.S. (2016). Moringa oleifera: A review on nutritive importance and its medicinal application. Food Sci. Hum. Wellness.

[440] Ghimire S., Subedi L., Acharya N., Gaire B.P. (2021). Moringa oleifera: A tree of life as a promising medicinal plant for neurodegenerative diseases. J. Agric. Food Chem..

[441] Manogna C., Margesan T. (2024). In silico and pharmacokinetic studies of glucomoringin from Moringa oleifera root for Alzheimer’s disease like pathology. Future Sci. OA.

[442] Lee R., Kim J.-H., Kim W.-W., Hwang S.-H., Choi S.-H., Kim J.-H., Cho I.-H., Kim M., Nah S.-Y. (2024). Emerging evidence that ginseng components improve cognition in subjective memory impairment, mild cognitive impairment, and early Alzheimer’s disease dementia. J. Ginseng Res..

[443] Ha Y., Lee R., Jeon S.H., Kim J.-H., Jo H.-S., Kwon T.W., Hwang S.-H., Lee J.K., Nah S.-Y., Cho I.-H. (2025). Korean Red Ginseng Marc-Derived Gintonin Improves Alzheimer’s Cognitive Dysfunction by Upregulating LPAR1. Am. J. Chin. Med..

[444] Lee M.Y., Kim M. (2024). Effects of Red ginseng on neuroinflammation in neurodegenerative diseases. J. Ginseng Res..

[445] Parmar S.A., Mayasa V., Nelson V.K., Divecha J. (2024). A Systemic Review of Ginseng and Its Activity on Coronary Heart Disease. Pharmacol. Res.-Mod. Chin. Med..

[446] Zhang L., Li D., Cao F., Xiao W., Zhao L., Ding G. (2018). Identification of human acetylcholinesterase inhibitors from the constituents of EGb761 by modeling docking and molecular dynamics simulations. Comb. Chem. High Throughput Screen..

[447] Song W., Zhao J., Yan X.-S., Fang X., Huo D.-S., Wang H., Jia J.-X., Yang Z.-J. (2019). Mechanisms associated with protective effects of ginkgo biloba leaf extracton in rat cerebral ischemia reperfusion injury. J. Toxicol. Environ. Health Part A.

[448] Nowak A., Kojder K., Zielonka-Brzezicka J., Wróbel J., Bosiacki M., Fabiańska M., Wróbel M., Sołek-Pastuszka J., Klimowicz A. (2021). The use of *Ginkgo biloba* L. as a neuroprotective agent in the Alzheimer’s disease. Front. Pharmacol..

[449] Peng Y., Chen Q., Xue Y.-H., Jin H., Liu S., Du M.-Q., Yao S.-Y. (2024). *Ginkgo biloba* and Its Chemical Components in the Management of Alzheimer’s Disease. Am. J. Chin. Med..

[450] Pagotto G.L.d.O., Santos L.M.O.d., Osman N., Lamas C.B., Laurindo L.F., Pomini K.T., Guissoni L.M., Lima E.P.d., Goulart R.d.A., Catharin V.M.S. (2024). *Ginkgo biloba*: A Leaf of Hope in the Fight against Alzheimer’s Dementia: Clinical Trial Systematic Review. Antioxidants.

[451] Pastorino G., Cornara L., Soares S., Rodrigues F., Oliveira M.B.P. (2018). Liquorice (*Glycyrrhiza glabra*): A phytochemical and pharmacological review. Phytother. Res..

[452] Damle M. (2014). *Glycyrrhiza glabra* (Liquorice)-a potent medicinal herb. Int. J. Herb. Med..

[453] Tripathi P.N., Lodhi A., Rai S.N., Nandi N.K., Dumoga S., Yadav P., Tiwari A.K., Singh S.K., El-Shorbagi A.-N.A., Chaudhary S. (2024). Review of Pharmacotherapeutic Targets in Alzheimer’s Disease and Its Management Using Traditional Medicinal Plants. Degener. Neurol. Neuromuscul. Dis..

[454] John O.O., Amarachi I.S., Chinazom A.P., Adaeze E., Kale M.B., Umare M.D., Upaganlawar A.B. (2022). Phytotherapy: A promising approach for the treatment of Alzheimer’s disease. Pharmacol. Res.-Mod. Chin. Med..

[455] Cole G.M., Teter B., Frautschy S.A. (2007). Neuroprotective effects of curcumin. The Molecular Targets and Therapeutic Uses of Curcumin in Health and Disease.

[456] Issuriya A., Kumarnsit E., Wattanapiromsakul C., Vongvatcharanon U. (2014). Histological studies of neuroprotective effects of *Curcuma longa* Linn. on neuronal loss induced by dexamethasone treatment in the rat hippocampus. Acta Histochem..

[457] Manhas A., Khanna V., Prakash P., Goyal D., Malasoni R., Naqvi A., Dwivedi A.K., Dikshit M., Jagavelu K. (2014). Curcuma oil reduces endothelial cell-mediated inflammation in postmyocardial ischemia/reperfusion in rats. J. Cardiovasc. Pharmacol..

[458] Meesarapee B., Thampithak A., Jaisin Y., Sanvarinda P., Suksamrarn A., Tuchinda P., Morales N.P., Sanvarinda Y. (2014). Curcumin I mediates neuroprotective effect through attenuation of quinoprotein formation, p-p38 MAPK expression, and caspase-3 activation in 6-hydroxydopamine treated SH-SY5Y cells. Phytother. Res..

[459] Omosa L., Midiwo J., Kuete V. (2017). *Curcuma longa*. Medicinal Spices and Vegetables from Africa.

[460] Meneses A.K.S., Salazar G.J.T., de Freitas M.M., de Lima S.G. (2024). *Curcuma longa*: A Natural Ally in Alzheimer’s Disease Management. Curcumin and Neurodegenerative Diseases: From Traditional to Translational Medicines.

[461] Seeram N.P., Zhang Y., Henning S.M., Lee R., Niu Y., Lin G., Heber D. (2006). Pistachio skin phenolics are destroyed by bleaching resulting in reduced antioxidative capacities. J. Agric. Food Chem..

[462] Bozorgi M., Memariani Z., Mobli M., Surmaghi M.S., Shams-Ardekani M., Rahimi R.F. (2013). A review of their traditional uses, phytochemistry, and pharmacology. Sci. World J..

[463] Polo-Hernandez E., Tello V., Arroyo A.A., Domínguez-Prieto M., de Castro F., Tabernero A., Medina J.M. (2014). Oleic acid synthesized by stearoyl-CoA desaturase (SCD-1) in the lateral periventricular zone of the developing rat brain mediates neuronal growth, migration and the arrangement of prospective synapses. Brain Res..

[464] Gao H., Yan P., Zhang S., Nie S., Huang F., Han H., Deng Q., Huang Q., Yang W., Wu H. (2016). Chronic alpha-linolenic acid treatment alleviates age-associated neuropathology: Roles of PERK/eIF2α signaling pathway. Brain Behav. Immun..

[465] Golchin L., Shabani M., Harandi S., Razavinasab M. (2015). Pistachio supplementation attenuates motor and cognition impairments induced by cisplatin or vincristine in rats. Adv. Biomed. Res..

[466] Hossain M.M.A.S., Sarker J., Rahman S.A., Rahman M.R.M. (2015). In vitro antioxidant and cholinesterase inhibitory activities of methanolic fruit extract of *Phyllanthus acidus*. BMC Complement. Med. Ther..

[467] Uddin M.S., Mamun A.A., Hossain M.S., Akter F., Iqbal M.A., Asaduzzaman M. (2016). Exploring the effect of Phyllanthus emblica L. on cognitive performance, brain antioxidant markers and acetylcholinesterase activity in rats: Promising natural gift for the mitigation of Alzheimer’s disease. Ann. Neurosci..

[468] Kennedy D., Scholey A.B., Tildesley N., Perry E., Wesnes K. (2002). Modulation of mood and cognitive performance following acute administration of *Melissa officinalis* (lemon balm). Pharmacol. Biochem. Behav..

[469] Kaczmarek-Kryszak K.A., Dobrzyńska M., Banaszak M., Drzymała-Czyż S. (2026). A comprehensive systematic review of human trials investigating herbal treatments for Alzheimer’s disease and dementia. Acta Neuropsychiatr..

[470] Balkrishna A., Joshi S., Shankar R., Prajapati U.B., Joshi R.A. (2026). Edible plants: Promising source for prevention and management of Parkinson’s and Alzheimer’s disease. Arch. Alzheimer’s Park. Dis..

[471] Mori K., Inatomi S., Ouchi K., Azumi Y., Tuchida T. (2009). Improving effects of the mushroom Yamabushitake (*Hericium erinaceus*) on mild cognitive impairment: A double-blind placebo-controlled clinical trial. Phytother. Res. Int. J. Devoted Pharmacol. Toxicol. Eval. Nat. Prod. Deriv..

[472] Daoust J., Farrar S., Grant A., Erfe M., Oliver P., Luna V., Moos J., Craft N. (2026). A randomized, double blind, placebo controlled study evaluating the impact of Hericium erinaceus (Lion’s Mane) on cognitive performance and subjective wellbeing. medRxiv.

[473] Tayarani-Najaran Z., Hadipour E., Ramazani S., Taghizadeh L., Ramazani E. (2026). Antioxidant, anti-inflammatory and cytoprotective effects of crocin, a bioactive constituent of saffron, in Alzheimer’s and Parkinson’s diseases with a focus on molecular mechanisms: A systematic review. Avicenna J. Phytomed..

[474] Maggi M.A., Mastromartino R., Piccardi M., Minnella A.M., Marangoni D., Di Marco S., Falsini B., Bisti S. (2026). Saffron as a Retinal Neuroprotectant: A Narrative Review of Preclinical Studies and Clinical Results. Antioxidants.

[475] Scholey A., Cox K., Pipingas A., White D. (2020). Curcumin improves hippocampal function in healthy older adults: A three month randomised controlled trial. Proc. Nutr. Soc..

[476] Zeppa L., Aguzzi C., Morelli M.B. (2025). Exploring the Therapeutic Potential of Natural Compounds and Plant Extracts in Human Health. Biomolecules.

[477] Deding U., Baatrup G., Kaalby L., Kobaek-Larsen M. (2023). Carrot intake and risk of developing cancer: A prospective cohort study. Nutrients.

[478] Du M., Liu X., Ji X., Wang Y., Liu X., Zhao C., Jin E., Gu Y., Wang H., Zhang F. (2025). Berberine alleviates enterotoxigenic Escherichia coli-induced intestinal mucosal barrier function damage in a piglet model by modulation of the intestinal microbiome. Front. Nutr..

[479] Ozorowski M., Wiciński M., Kuźmiński O., Wojciechowski P., Siedlecki Z., Śniegocki M., Włodarczyk E. (2025). The Effects of Quercetin on Vascular Endothelium, Inflammation, Cardiovascular Disease and Lipid Metabolism—A Review. Nutrients.

[480] Li F., Huang W., Yang C., Yu B., Wu Q., Du Z. (2025). Epigallocatechin gallate induces an up-regulation of LDLR accompanied by a reduction of idol in Hepg2 cells. Nuclleus.

[481] Wang Z.-y., Deng Y.-l., Zhou T.-y., Liu Y., Cao Y. (2025). Effects of natural extracts in cognitive function of healthy adults: A systematic review and network meta-analysis. Front. Pharmacol..

[482] Dolan E.W. Study Identifies Top-Performing Natural Extracts for Improving Cognitive Function. https://www.psypost.org/study-identifies-top-performing-natural-extracts-for-improving-cognitive-function/.

[483] The Good Trade (2025). 10 Natural Supplements For Better Brain Health. https://www.thegoodtrade.com/features/brain-focus-supplements/.

[484] Lee J., Kwon S., Jin C., Cho S.-Y., Park S.-U., Jung W.-S., Moon S.-K., Park J.-M., Ko C.-N., Cho K.-H. (2022). Traditional east asian herbal medicine treatment for Alzheimer’s Disease: A systematic review and meta-analysis. Pharmaceuticals.

[485] Dong L., May B.H., Feng M., Hyde A.J., Tan H.Y., Guo X., Zhang A.L., Lu C., Xue C.C. (2016). Chinese herbal medicine for mild cognitive impairment: A systematic review and meta-analysis of cognitive outcomes. Phytother. Res..

[486] Tian J.-Z., Shi J., Ni J.-N., Wei M.-Q., Zhang X.-K., Chen K.-J., Wang Y.-Y. (2019). Sequential therapy based on evolvement of patterns: A new model for treatment of Alzheimer’s disease. Chin. J. Integr. Med..

[487] Lin Z., Huang T., Zheng G., Chen R., Yao M., Liu W., Li S. (2022). Study on the correlation between Chinese medicine syndrome and cognitive dysfunction in mild cognitive impairment. Evid.-Based Complement. Altern. Med..

[488] Chen K.-D., Chang P.-T., Ping Y.-H., Lee H.-C., Yeh C.-W., Wang P.-N. (2011). Gene expression profiling of peripheral blood leukocytes identifies and validates ABCB1 as a novel biomarker for Alzheimer’s disease. Neurobiol. Dis..

[489] Yang Z., Xie D., Chen S., Ou A., Lao Y. (2007). Initial study on disposition of chinese medical symptoms and signs of mild cognitive impairment for Elder People. World J. Integr. Tradit. West. Med..

[490] Yeh C.-W., Liu H.-K., Lin L.-C., Liou K.-T., Huang Y.-C., Lin C.-H., Tzeng T.-T., Shie F.-S., Tsay H.-J., Shiao Y.-J. (2017). Xuefu Zhuyu decoction ameliorates obesity, hepatic steatosis, neuroinflammation, amyloid deposition and cognition impairment in metabolically stressed APPswe/PS1dE9 mice. J. Ethnopharmacol..

[491] Naughton B.J., Duncan F.J., Murrey D.A., Meadows A.S., Newsom D.E., Stoicea N., White P., Scharre D.W., Mccarty D.M., Fu H. (2014). Blood genome-wide transcriptional profiles reflect broad molecular impairments and strong blood-brain links in Alzheimer’s disease. J. Alzheimer’s Dis..

[492] Han G., Wang J., Zeng F., Feng X., Yu J., Cao H.-Y., Yi X., Zhou H., Jin L.-W., Duan Y. (2013). Characteristic transformation of blood transcriptome in Alzheimer’s disease. J. Alzheimer’s Dis..

[493] Bottero V., Potashkin J.A. (2019). Meta-analysis of gene expression changes in the blood of patients with mild cognitive impairment and Alzheimer’s disease dementia. Int. J. Mol. Sci..

[494] Leandro G.S., Evangelista A.F., Lobo R.R., Xavier D.J., Moriguti J.C., Sakamoto-Hojo E.T. (2018). Changes in expression profiles revealed by transcriptomic analysis in peripheral blood mononuclear cells of Alzheimer’s disease patients. J. Alzheimer’s Dis..

[495] Moradi E., Marttinen M., Häkkinen T., Hiltunen M., Nykter M. (2019). Supervised pathway analysis of blood gene expression profiles in Alzheimer’s disease. Neurobiol. Aging.

[496] Song L., Chen J., Lo C.-Y.Z., Guo Q., Feng J., Zhao X.-M. (2022). Impaired type I interferon signaling activity implicated in the peripheral blood transcriptome of preclinical Alzheimer’s disease. eBioMedicine.

[497] Proitsi P., Lee S.H., Lunnon K., Keohane A., Powell J., Troakes C., Al-Sarraj S., Furney S., Soininen H., Kłoszewska I. (2014). Alzheimer’s disease susceptibility variants in the MS4A6A gene are associated with altered levels of MS4A6A expression in blood. Neurobiol. Aging.

[498] Shigemizu D., Mori T., Akiyama S., Higaki S., Watanabe H., Sakurai T., Niida S., Ozaki K. (2020). Identification of potential blood biomarkers for early diagnosis of Alzheimer’s disease through RNA sequencing analysis. Alzheimer’s Res. Ther..

[499] Roed L., Grave G., Lindahl T., Rian E., Horndalsveen P.O., Lannfelt L., Nilsson C., Swenson F., Lönneborg A., Sharma P. (2013). Prediction of mild cognitive impairment that evolves into Alzheimer’s disease dementia within two years using a gene expression signature in blood: A pilot study. J. Alzheimer’s Dis..

[500] Lunnon K., Sattlecker M., Furney S.J., Coppola G., Simmons A., Proitsi P., Lupton M.K., Lourdusamy A., Johnston C., Soininen H. (2013). A blood gene expression marker of early Alzheimer’s disease. J. Alzheimer’s Dis..

[501] Pievani M., de Haan W., Wu T., Seeley W.W., Frisoni G.B. (2011). Functional network disruption in the degenerative dementias. Lancet Neurol..

[502] Balthazar M.L.F., de Campos B.M., Franco A.R., Damasceno B.P., Cendes F. (2014). Whole cortical and default mode network mean functional connectivity as potential biomarkers for mild Alzheimer’s disease. Psychiatry Res. Neuroimaging.

[503] Zhang J., Liu Z., Zhang H., Yang C., Li H., Li X., Chen K., Zhang Z. (2016). A two-year treatment of amnestic mild cognitive impairment using a compound Chinese medicine: A placebo controlled randomized trial. Sci. Rep..

[504] Szelies B., Mielke R., Herholz K., Heiss W.-D. (1994). Quantitative topographical EEG compared to FDG PET for classification of vascular and degenerative dementia. Electroencephalogr. Clin. Neurophysiol..

[505] Smailovic U., Koenig T., Kåreholt I., Andersson T., Kramberger M.G., Winblad B., Jelic V. (2018). Quantitative EEG power and synchronization correlate with Alzheimer’s disease CSF biomarkers. Neurobiol. Aging.

[506] Matsuoka T., Narumoto J., Shibata K., Okamura A., Taniguchi S., Kitabayashi Y., Fukui K. (2012). Effect of Toki-Shakuyaku-San on Regional Cerebral Blood Flow in Patients with Mild Cognitive Impairment and Alzheimer′ s Disease. Evid.-Based Complement. Altern. Med..

[507] Yamaguchi S., Matsubara M., Kobayashi S. (2004). Event-related brain potential changes after Choto-san administration in stroke patients with mild cognitive impairments. Psychopharmacology.

[508] Oishi M., Mochizuki Y., Takasu T., Chao E., Nakamura S. (1998). Effectiveness of traditional Chinese medicine in Alzheimer disease. Alzheimer Dis. Assoc. Disord..

[509] Heo J.-H., Park M.-H., Lee J.-H. (2016). Effect of Korean red ginseng on cognitive function and quantitative EEG in patients with Alzheimer’s disease: A preliminary study. J. Altern. Complement. Med..

[510] Zhang J., Wang Z., Xu S., Chen Y., Chen K., Liu L., Wang Y., Guo R., Zhang Z. (2014). The effects of CCRC on cognition and brain activity in aMCI patients: A pilot placebo controlled BOLD fMRI study. Curr. Alzheimer Res..

[511] Warren A., Wynia Z., Corr P.G., Devin M.F., Celikkol Z., Gordon L., Farah M., Karam M., Villarreal D., Jackson S.A. (2026). The microbiota–gut–brain axis in mild cognitive impairment and Alzheimer’s disease: A scoping review of human studies. Alzheimer’s Dement..

[512] Wu W., Meng T., Han L., Jin F., Han P., Zhou Y. (2025). Bridging traditional Chinese medicine and Alzheimer’s disease: The pivotal role of gut microbiota in multitarget therapeutic mechanisms. Front. Pharmacol..

[513] Rajini P., Muralidhara M. (2023). Therapeutic efficacy of ayurvedic polyherbal formulations (PHF): Interactive mechanisms and broad-spectrum activities against neurological disorders. Ayurvedic Herbal Preparations in Neurological Disorders.

[514] Bhajan S.K., Bishwas A.K., Dutta B., Bala A., Aktary N., Park S., Rahman M., Choi M., Choi J., Akter S. (2026). Recent Advancements in Bioactive Natural Products and Nanoparticle-Mediated Drug Delivery in Cancer Therapy. Int. J. Mol. Sci..

[515] Anand A., Gautam P., Ojha S. (2024). Application of nanotechnology for herbal medicine development: A review. Lett. Drug Des. Discov..

[516] Halim P., Tan M.L., Yumiko, Lu F.C., Dalimunthe A., Tallei T.E., Rahman M., Rani A., Kim B., de Azambuja Ribeiro R.I.M. (2026). Nanotechnology in Lung Cancer: Enhancing Targeted Drug Delivery and Diagnostic Precision. Results Surf. Interfaces.

[517] Chakraborty K., Shivakumar A., Ramachandran S. (2016). Nano-technology in herbal medicines: A review. Int. J. Herb. Med..

[518] Pan R., Liu G., Zeng Y., He X., Ma Z., Wei Y., Chen S., Yang L., Tao L. (2021). A multi-responsive self-healing hydrogel for controlled release of curcumin. Polym. Chem..

[519] Kumar M., Keshwania P., Chopra S., Mahmood S., Bhatia A. (2023). Therapeutic potential of nanocarrier-mediated delivery of phytoconstituents for wound healing: Their current status and future perspective. Aaps Pharmscitech.

[520] Zhou G., Xu R., Groth T., Wang Y., Yuan X., Ye H., Dou X. (2024). The combination of bioactive herbal compounds with biomaterials for regenerative medicine. Tissue Eng. Part B Rev..

[521] Shishir M.R.I., Gowd V., Suo H., Wang M., Wang Q., Chen F., Cheng K.W. (2021). Advances in smart delivery of food bioactive compounds using stimuli-responsive carriers: Responsive mechanism, contemporary challenges, and prospects. Compr. Rev. Food Sci. Food Saf..

[522] Patel P., Garala K., Singh S., Prajapati B.G., Chittasupho C. (2024). Lipid-based nanoparticles in delivering bioactive compounds for improving therapeutic efficacy. Pharmaceuticals.

[523] Priya V.M.H., Kumaran A. (2023). Recent trends in phytosome nanocarriers for improved bioavailability and uptake of herbal drugs. Pharm. Sci..

[524] Jain S.D., Shrivastava S.K., Agrawal A., Gupta A.K. (2024). WHO guidelines for quality control of herbal medicines: From cultivation to consumption. Int. J. Pharm. Chem. Anal..

[525] Dawoud A.D.H. (2025). Standardization of Medicinal Plants: Ensuring Quality, Safety, and Global Regulatory Compliance in Herbal Drug Development. Plant Biotechnol. Persa.

[526] Senapati A., Basak S., Rangan L. (2022). A review on application of DNA barcoding technology for rapid molecular diagnostics of adulterants in herbal medicine. Drug Saf..

[527] Khan A., Abdulaziz Al-Hamoud G., Amina M., Alam P., Hawwal M.F., Fantoukh O.I. (2026). Role of herbal extracts in modulating drug metabolism: Implications for pharmacokinetics, enzyme regulation, and therapeutic outcome. Drug Metab. Rev..

[528] Soni S., Rathee S., Tekade M., Bharti A., Gupta R., Tekade R.K. (2026). Translational Case Studies on Marketed Phytomedicines: From Traditional Knowledge to Global Therapeutics. Biomolecular and Safety Considerations of Phytopharmaceuticals.

[529] Badria F.A., Elgazar A.A. (2026). The Burden of Inappropriate Polypharmacy: Approaches to Improve Medication Safety. Polypharmacy and Inappropriate Medication Use.

[530] Kumar A. (2026). Potential Herb-Drug Interaction and Their Clinical Implications: Pharmacokoinetics and Pharmacodynamic Considerations. Int. J. Multidiscip. Res..

[531] Mai N.T.Q., Hieu N.V., Ngan T.T., Van Anh T., Van Linh P., Thu Phuong N.T. (2025). Impact of *Ginkgo biloba* drug interactions on bleeding risk and coagulation profiles: A comprehensive analysis. PLoS ONE.

[532] Sefidmooye Azar P., Akhlaghi S., Shariat-Madar Z., Mahdi F. (2026). Cognitive-Enhancing Effects of Bioactive Compounds and Traditional Herbal Medicines in Elderly Patients with Metabolic Syndrome. Biomolecules.

[533] Stough C., Lloyd J., Clarke J., Downey L., Hutchison C., Rodgers T., Nathan P. (2001). The chronic effects of an extract of *Bacopa monniera* (Brahmi) on cognitive function in healthy human subjects. Psychopharmacology.

[534] Birks J., Evans J.G. (2009). *Ginkgo biloba* for cognitive impairment and dementia. Cochrane Database Syst. Rev..

[535] Halegoua-DeMarzio D., Navarro V. (2025). Challenges in herbal-induced liver injury identification and prevention. Liver Int..

[536] Remenapp A., Coyle K., Orange T., Lynch T., Hooper D., Hooper S., Conway K., Hausenblas H. (2022). Efficacy of *Withania somnifera* supplementation on adult’s cognition and mood. J. Ayurveda Integr. Med..

[537] Scholey A., De Longis E., Hudry J., Owen L. (2026). Effects of *Panax ginseng* and *Panax quinquefolius* on Cognitive Function: A Systematic Review. J. Cogn. Enhanc..

[538] Lee S.-T., Chu K., Sim J.-Y., Heo J.-H., Kim M. (2008). *Panax ginseng* enhances cognitive performance in Alzheimer disease. Alzheimer Dis. Assoc. Disord..

[539] Yang J., Zhu H., Zhang Q., Dai Y., Yao Q., Nie X. (2026). Charting the Phytopharmacological Frontier of *Centella asiatica*: A Quarter-Century Bibliometric Atlas Unlocking Translational Potential. Rev. Bras. De Farmacogn..

[540] Yu L., Li N., Li B., Ye K.X., Guo J., Shan J., Cao L., Song M., Wang Y., Lee T.-S. (2025). Targeting cognitive aging with curcumin supplementation: A systematic review and meta-analysis. J. Prev. Alzheimer’s Dis..

[541] Rashid A., Umer A., Bashir M., Awan A., Maqbool W., Khalid N., Altaf M., Kharl H.A.A. (2026). Multi-Target Hepatoprotective Mechanisms of *Glycyrrhiza glabra*: Molecular Pathways, Experimental Evidence, and Clinical Translation. J. Health Wellness Community Res..

[542] Hatayama K., Kono K., Okuma K., Masuyama H. (2025). Effect of a specific food intervention with Tamogitake mushroom, Moringa leaves, or rice bran on intestinal microbiota and cognitive function in elderly Japanese. Front. Nutr..

[543] González-Sánchez M., Ramírez-Expósito M.J., Martínez-Martos J.M. (2025). Pathophysiology, clinical heterogeneity, and therapeutic advances in amyotrophic lateral sclerosis: A comprehensive review of molecular mechanisms, diagnostic challenges, and multidisciplinary management strategies. Life.

[544] Dong J.M., Zhong H. (2025). Systematic Review: Proteomics-Driven Multi-Omics Integration for Alzheimer’s Disease Pathology and Precision Medicine. Neurol. Int..

[545] Sheng C., Du W., Liang Y., Xu P., Ding Q., Chen X., Jia S., Wang X. (2023). An integrated neuroimaging-omics approach for the gut-brain communication pathways in Alzheimer’s disease. Front. Aging Neurosci..

[546] Loh J.S., Mak W.Q., Tan L.K.S., Ng C.X., Chan H.H., Yeow S.H., Foo J.B., Ong Y.S., How C.W., Khaw K.Y. (2024). Microbiota–gut–brain axis and its therapeutic applications in neurodegenerative diseases. Signal Transduct. Target. Ther..

[547] Wu D., Wang H., Ni Y., Lu Y., Wang Y., Li H., Chen L. (2025). Decoding the gut–brain axis: Toward AI-driven integration of neuroimaging and gut microbiota in human health. Vis. Comput..

[548] Kumar R., Nagraik R., Lakhanpal S., Abomughaid M.M., Jha N.K., Gupta R. (2025). Artificial intelligence in gut microbiome research: Toward predictive diagnostics for neurodegenerative disorders. Acta Microbiol. Immunol. Hung..

[549] Ness S., Adom S., Shepherd N.J. (2026). AI-Driven Detection of Inherited Neurological Disorders Using Genomic and Multi-Omics Data. Int. Neuropsychiatr. Dis. J..

[550] Balkrishna A., Sharma N., Srivastava D., Kukreti A., Srivastava S., Arya V. (2024). Exploring the Safety, Efficacy, and Bioactivity of Herbal Medicines: Bridging Traditional Wisdom and Modern Science in Healthcare. Future Integr. Med..

[551] Rudrapal M., Chetia D. (2021). Herbal Drugs: Efficacy, Toxicity, and Safety Issues. Evidence Based Validation of Traditional Medicines.

[552] Koonrungsesomboon N., Sakuludomkan C., Na Takuathung M., Klinjan P., Sawong S., Perera P.K. (2024). Study design of herbal medicine clinical trials: A descriptive analysis of published studies investigating the effects of herbal medicinal products on human participants. BMC Complement. Med. Ther..

[553] Birks J.S. (2006). Cholinesterase inhibitors for Alzheimer’s disease. Cochrane Database Syst. Rev..

[554] Gagnier J.J., DeMelo J., Boon H., Rochon P., Bombardier C. (2006). Quality of reporting of randomized controlled trials of herbal medicine interventions. Am. J. Med..

[555] Wilson V., Maulik S.K. (2018). Herb-drug interactions in neurological disorders: A critical appraisal. Curr. Drug Metab..

[556] Bhattacharya T., Soares G.A.B.E., Chopra H., Rahman M.M., Hasan Z., Swain S.S., Cavalu S. (2022). Applications of phyto-nanotechnology for the treatment of neurodegenerative disorders. Materials.

[557] Gagnier J.J., Boon H., Rochon P., Moher D., Barnes J., Bombardier C., CONSORT Group (2006). Reporting randomized, controlled trials of herbal interventions: An elaborated CONSORT statement. Ann. Intern. Med..

[558] Ballotin V.R., Bigarella L.G., de Mello Brandão A.B., Balbinot R.A., Balbinot S.S., Soldera J. (2021). Herb-induced liver injury: Systematic review and meta-analysis. World J. Clin. Cases.

[559] Ma Z.-T., Shi Z., Xiao X.-H., Wang J.-B. (2023). New insights into herb-induced liver injury. Antioxid. Redox Signal..

[560] Shen C., Ren Z.Y., Lan H.D., Kong L.Y., Yang M., Su Y.Z., Yue X.L., Wan Z.L., Xiao L.X., Chen P.P. (2025). Perspectives, experiences, and practices of healthcare professionals and patients towards herb–drug interaction: A systematic review of qualitative studies. Phytother. Res..

[561] Puthiyedath R., Pillai Z.S. (2025). Drug–herb interactions: A challenge and clinical concern in primary healthcare. Front. Med..

[562] Park J.-H. (2008). Evidence-based herbal medicine in efficacy and safety assessments. Adv. Tradit. Med..

[563] Soldera J. (2024). Insights into skullcap herb-induced liver injury. World J. Hepatol..

[564] Kunle O.F., Egharevba H.O., Ahmadu P.O. (2012). Standardization of herbal medicines-A review. Int. J. Biodivers. Conserv..

[565] Cave A.E., Chang D.H., Münch G.W., Steiner-Lim G.Z. (2023). A systematic review of the safety and efficacy on cognitive function of herbal and nutritional medicines in older adults with and without subjective cognitive impairment. Syst. Rev..

[566] Moradi S.Z., Momtaz S., Bayrami Z., Farzaei M.H., Abdollahi M. (2020). Nanoformulations of herbal extracts in treatment of neurodegenerative disorders. Front. Bioeng. Biotechnol..

[567] Yadav V., Guin S., Nayak S., Mishra A. (2024). Herbal Approaches for the Management of Neurological Disorders. Drug Delivery Strategies in Neurological Disorders: Challenges and Opportunities.

